# Design of the elusive proteinaceous oxygen donor copper site suggests a promising future for copper for MRI contrast agents

**DOI:** 10.1073/pnas.2219036120

**Published:** 2023-06-26

**Authors:** Anokhi Shah, Michael J. Taylor, Giulia Molinaro, Sellamuthu Anbu, Margaux Verdu, Lucy Jennings, Iuliia Mikulska, Sofia Diaz-Moreno, Hassane EL Mkami, Graham M. Smith, Melanie M. Britton, Janet E. Lovett, Anna F. A. Peacock

**Affiliations:** ^a^Scottish Universities Physics Alliance School of Physics and Astronomy, University of St Andrews, St Andrews KY16 9SS, United Kingdom; ^b^Biomedical Sciences Research Complex, University of St Andrews, St Andrews KY16 9ST, United Kingdom; ^c^School of Chemistry, University of Birmingham, Edgbaston B15 2TT, United Kingdom; ^d^Diamond Light Source, Harwell Science and Innovation Campus, OX11 0DE, Didcot, United Kingdom

**Keywords:** bioinorganic chemistry, coiled coil, contrast agents, MRI, protein design

## Abstract

We report the preparation and spectroscopic characterization of a highly elusive copper site bound exclusively to oxygen donor atoms within a protein scaffold. Despite copper generally being considered unsuitable for use in MRI contrast agents, which in the clinic are largely Gd(III) based, the designed copper coiled coil displays relaxivity values equal to, or superior than, those of the Gd(III) analog at clinical field strengths. The creation of this new-to-biology proteinaceous CuO_x_-binding site demonstrates the power of the de novo peptide design approach to access chemistry for abiological applications, such as for the development of MRI contrast agents.

## Significance

Metal ions perform many essential roles in biology, with copper being a metal that nature has chosen to use extensively. Using a miniature artificial protein design strategy, it has been possible to prepare a new-to-biology copper–binding site, one which we were surprised to find is absent in nature. The resulting copper site has been shown to display real potential for use in MRI contrast agents, thereby challenging the existing dogma that copper is unsuitable for use in MRI. This example showcases that designing abiological metal ion sites is a powerful approach for accessing new tools or agents for applications beyond the repertoire of biology.

New-to-biology metal sites could lead to new function beyond what biology is currently capable of.

De novo designed miniature protein scaffolds have been used as ligands for metal ions, primarily in an effort to structurally and functionally mimic biological sites. These include numerous mimics of biologically relevant metal ion sites engineered within the hydrophobic core of coiled coils or helical bundles (1, 2).

Copper sites are ubiquitous in nature, with diverse roles ranging from oxygen binding, catalysis, and electron transfer. The copper coordination chemistry is likewise diverse, with examples of mononuclear, dinuclear, and tetranuclear copper sites, which can be distorted with mixtures of strongly and weakly coordinated ligands. A structural genomics survey reports that almost all Cu(I) is found bound to sulfur donors, while almost all of the harder Cu(II) sites are coordinated by at least one nitrogen donor (3). Biological copper sites exist with exclusive nitrogen coordination [Type 3 (Cu_T3_) and Type B (Cu_B_)] and exclusive sulfur coordination (Cu-S_Cys_). Still, the large majority are mixed donor sites, CuO_x_N_y_S_z_ [including Type 1 (Cu_T1_), Type 2 (Cu_T2_), Type 1.5 (Cu_1.5_), Type A (Cu_A_), Type B (Cu_B_), Type Z (Cu_Z_), and Type 0 (Cu_T0_)] (4, 5). A number of reports exist of Cu_T1_ (6), Cu_T1.5_ (7), Cu_T2_ (8–10), Cu_T3_ (11), Cu_A_ (12), and Cu-S_Cys_ (13–15) biological and mixed donor copper sites reproduced within coiled coils and helical bundles (5–20).

Given the diversity of copper sites in nature, it is surprising that copper bound to an exclusive oxygen donor set within a protein scaffold is either incredibly rare or does not exist. A search of the protein databank reveals a large number of copper ions bound to the surface of a protein through one or two amino acid O donor ligands, which are likely to be nonspecifically bound copper or incorrectly assigned electron density (21). To the best of our knowledge, there are only two potential examples of crystal structures featuring copper coordinated exclusively to oxygen donor atoms bound within a protein scaffold. For one of these examples, PDB 4B61 (22), we believe that the electron density may have been incorrectly assigned, given the assignment as Ca/Na in three closely related crystal structures: PDB 3RBH (23), 4AZL, and 4AFK (22). The second example features a copper bound in a distorted tetrahedron geometry to a bacterial iron import protein EfeO, PDB 5Y4C. However, the identity of the metal in this site was not unambiguously established as Cu(II) rather than Fe(III) or Zn(II), especially given that a mutant which lacks two of the Glu ligands binds copper with the same affinity (24).

Copper and complexes thereof are also widely used in chemistry and materials science, and a range of small molecules (25), metallopolymers (26), and solid-state materials (27, 28) feature exclusively oxygen coordination. Though the therapeutic potential of copper complexes has been investigated (29), Cu(II) complexes have been largely ignored for use in MRI contrast agents (CAs), due to copper’s perceived poor relaxivity (30, 31). This is despite concerns about the long-term safety of Gd-based CAs [which currently dominate the MRI CA market], which has increased interest in developing Gd-free CAs, driving the development of Mn-based MRI CAs, among others (32).

Here, we report the proteinaceous CuO_x_ site and crucially demonstrate that this new-to-biology copper site is capable of high MRI relaxivity, thereby challenging the established view that Cu(II) is unsuitable for use in MRI CAs.

Our findings demonstrate the power of the miniature artificial metalloprotein design approach, to access abiological sites with new function for abiological applications. Expanding and evolving the range of proteinaceous metal ion sites beyond the relatively restrictive toolbox of biology, will provide access to currently untapped inorganic chemistry, for use in synthetic biology.

## Results and Discussion

### Establishing Copper Binding.

**Fig. 1.**
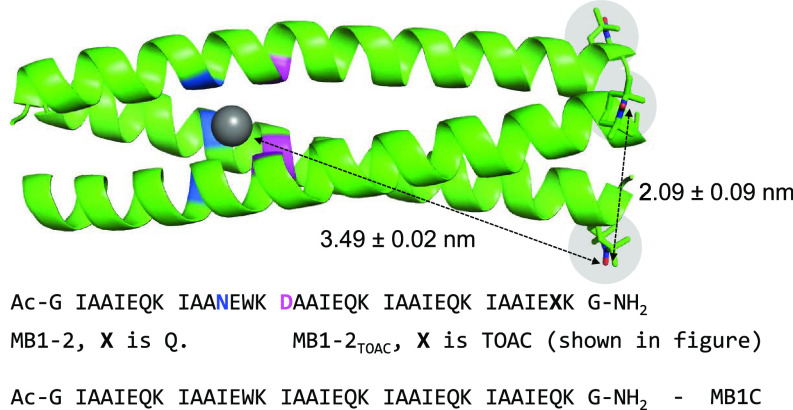
Sequence information for the peptides used in this work: MB1-2 (X = Q), MB1-2_TOAC_ (X = TOAC), and MB1C, and cartoon representation, rendered in PyMOL (v. 1.4 Schrödinger, LLC), of a model of the metal-bound MB1-2_TOAC_ trimer. The TOAC spin-label was added using ALLNOX (36, 37). The main-chain atoms are shown as helical ribbons with the backbone location of the Asn (blue) and Asp (pink) highlighted, the bound metal as a gray sphere, and the three TOAC spin labels in stick form, highlighted in gray circles, toward the C terminus on the right.

Copper has a high affinity for the nitrogen donor atoms of His and the sulfur of Cys/Met, as well as the oxygen donors of the carboxylic acid side chains of Glu/Asp. A miniature protein scaffold with: 1) a restricted amino acid set, which specifically lacks His, Cys, and Met residues; 2) a flexible oxygen-rich binding site; 3) buried in a protein-like hydrophobic core; and 4) which lacks other designed metal binding sites, should provide only one viable binding site for copper. Our design is based on a parallel three-stranded coiled coil constructed using the heptad repeat approach, I*_a_*A*_b_*A*_c_*I*_d_*E*_e_*Q*_f_*K*_g_*, which features adjacent Asn and Asp layers within the hydrophobic core, and that we have previously reported binds to lanthanide ions (La, Ce, Pr, Nd, Sm, Eu, Gd, Tb, Dy, Er, Yb, Lu), see Fig. 1 (33–35). The proposed binding site contains a layer of negatively charged carboxylates (Asp) adjacent to a neutral layer (an additional negatively charged layer would prevent peptide folding) of Asn residues, thereby generating a three-dimensional oxygen-rich environment (note Asn contains both O and N donors) from which the Cu(II) can “pull out” the appropriately positioned donor atoms to satisfy its coordination preferences.

A computational model of the copper-bound MB1-2 peptide trimer, generated using ColabFold (38) and Metal3D (39), is in good agreement with our proposed design (Fig. 1 and *SI Appendix*, Fig. S1).

**Fig. 2.**
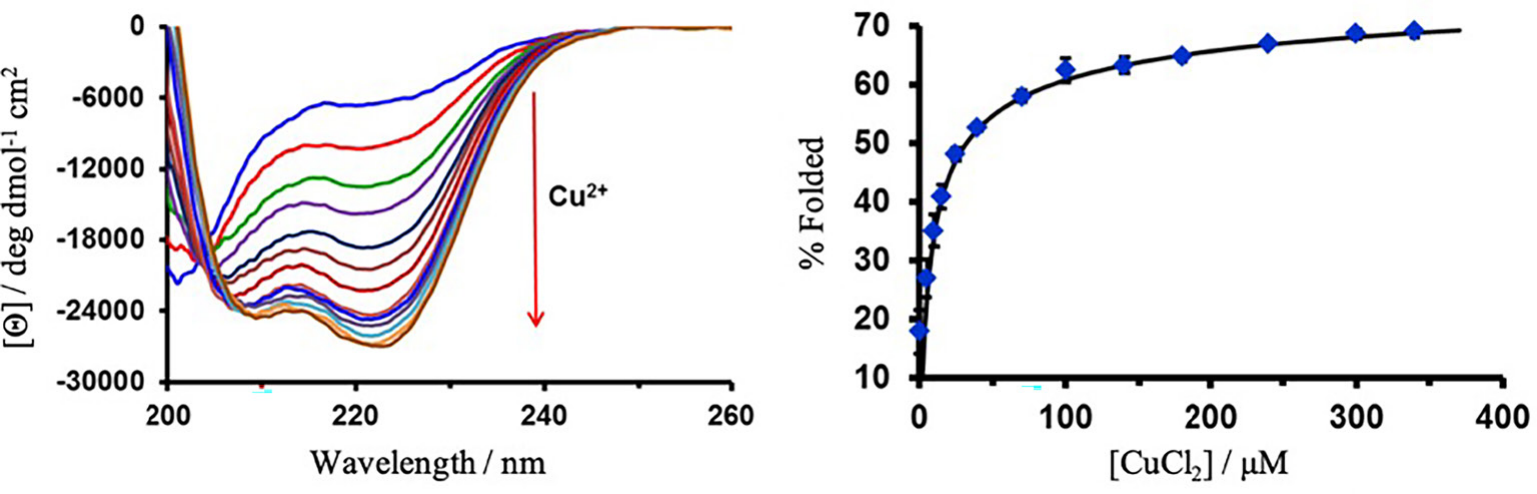
CuCl_2_ (0 to 340 µM) titration into 30 μM MB1-2 peptide monomer in 10 mM HEPES buffer pH 7.0, monitored by CD. The plot of % folded, based on the molar ellipticity at 222 nm, as a function of CuCl_2_ concentration, is shown fit to a nonlinear least-squares fitting based on M + 3L ↔ ML_3_ binding model using DynaFit (40). Error bars are shown for the SD error of three independent repeats.

The peptide, MB1-2, is poorly folded in the absence of a metal ion, but lanthanide binding was previously found to induce coiled coil folding (33, 34). To establish experimentally whether copper binds to the peptide, a copper-binding titration, monitored by circular dichroism (CD) spectroscopy, was performed. The addition of increasing aliquots of CuCl_2_ is accompanied by an increase in the negative molar ellipticity, to yield a well-folded α-helical peptide (c.a. 69 ± 1% for 30 µM MB1-2 monomer) and the binding curve is shown in Fig. 2. The data are consistent with Cu(II) binding to the poorly structured peptide and inducing folding. A plot of percentage folding as a function of Cu(II) concentration (into 30 µM MB1-2 monomer) can be fit to a 3:1 MB1-2:Cu(II) binding model, but with a weaker apparent affinity, log *K* 4.7 ± 0.1, compared to the apparent log *K* 5.5 ± 0.2 for Tb(III) (34). An analogous experiment monitoring the quenching of the Trp fluorescence on addition of increasing aliquots of CuCl_2_ could be fit to the same binding model, yielding a similar apparent log *K* 4.6 ± 0.2, see *SI Appendix*, Fig. S2. Given that Cu(II) binding, coiled coil assembly, and folding are coupled processes, the apparent binding affinities are reported. *SI Appendix*, Fig. S3 shows that the extent of peptide folding and the apparent binding affinity appears tighter as the total peptide concentration increases (data shown for 17, 30, 90, 120, and 150 µM MB1-2 monomer solutions). Though not conclusive proof given the limitations of such analysis, due to the weak binding and complex nature of potential binding equilibria, a CD Job’s plot analysis is consistent with this same 3:1 binding model, see *SI Appendix*, Fig. S4. A CD kinetic study and thermal denaturation experiment provides evidence that the kinetics associated with Cu(II) binding are fast, see *SI Appendix*, Fig. S5, and that the coiled coil is stabilized on binding Cu(II), see *SI Appendix*, Fig. S6.

### XAS Analysis.

**Fig. 3.**
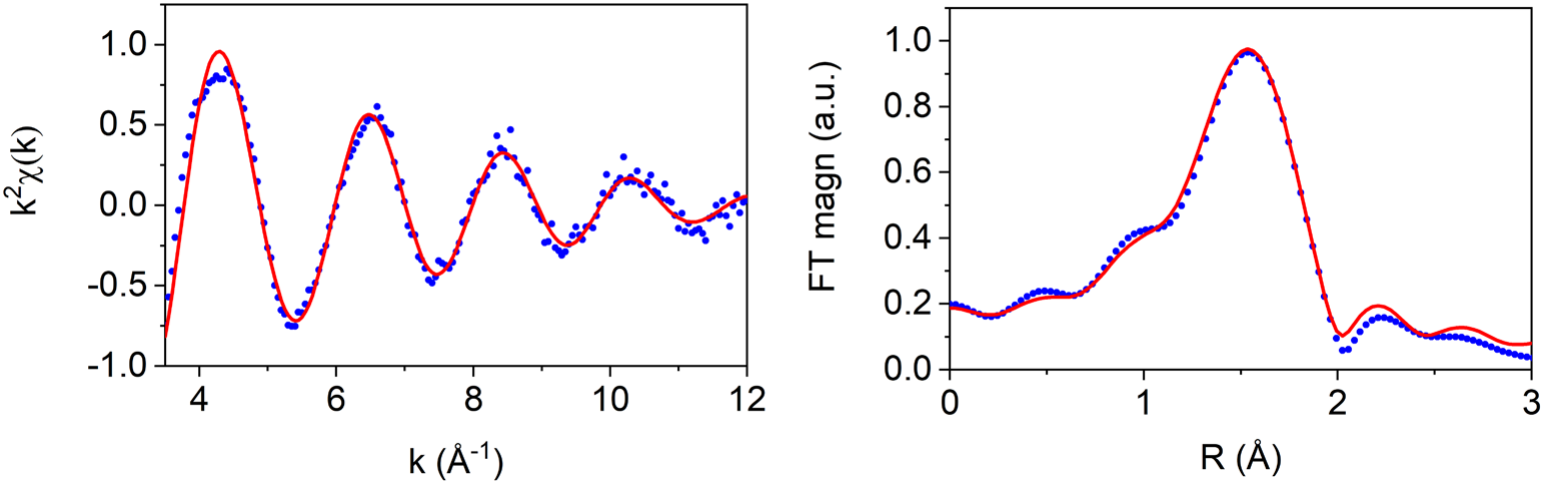
k^2^-weighted Cu K-edge-extracted EXAFS signal (*Left*) and corresponding Fourier transform magnitudes (*Right*) for a 1 mM Cu(II) solution in the presence of 6.6 mM MB1-2 peptide monomer and 50% glycerol in 50 mM HEPES buffer pH 7.0. The experimental data (blue circles) are shown with the best fit (solid red line) for a square pyramidal CuO_5_ structure.

Cu K-edge X-ray absorption spectroscopy (XAS) data analysis was undertaken of 1 mM Cu(MB1-2)_3_ in 50 mM HEPES buffer pH 7.0 and 50% glycerol, a common glassing agent routinely used at the cryogenic temperatures employed in XAS, in an effort to characterize the Cu(II)-binding environment. The absorption spectrum of the sample is clearly different from that of the control sample, CuCl_2_ in the absence of MB1-2 peptide, indicating that the local structure around Cu(II) ions is different in the two specimens (*SI Appendix*, Fig. S7). The extended X-ray absorption fine structure (EXAFS) spectrum of the sample could fit with good agreement to various models for CuO_x_, including trigonal planar, trigonal pyramidal, square pyramidal, and Jahn–Teller distorted octahedral, corresponding to 3, 4, 5, or 6 oxygen atoms around the copper center (Fig. 3 and *SI Appendix*, Fig. S8). The best-fit parameters from the EXAFS analysis for the four models considered are reported in *SI Appendix*, Table S1. All the four models yield good fits to the experimental spectrum, so it has not been possible to distinguish between them conclusively. In all cases, the data are consistent with a first Cu–O_1_ shell with bond lengths ranging from 1.93 to 1.96 Å, with a second Cu–O_2_ shell with bond lengths at 2.30-2.35 Å for the trigonal pyramidal, square pyramidal, and Jahn–Teller distorted octahedral geometries. However, given that EXAFS cannot distinguish between oxygen and nitrogen donor atoms, due to their similar atomic numbers, it has not been possible to rule out the existence of nitrogen atoms coordinating to the copper center.

Given glycerol’s established role as a protein-stabilizing agent (41, 42), including reports of its stabilization of coiled coils (43), we investigated its impact on Cu(II) binding to MB1-2. Glycerol was found to enhance MB1-2 folding (*SI Appendix*, Figs. S9 and S10), which is in turn accompanied by the formation of a more preorganized metal-binding site and the observation of enhanced Cu(II) binding by XAS (*SI Appendix*, Figs. S7 and S11). Full details and discussion can be found in *SI Appendix*.

### EPR Characterization of the Binding Site.

Given the presence of oxygen and nitrogen donors in MB1-2, the prevalence of nitrogen-coordinated Cu(II) in biology, and the inability of EXAFS to distinguish between oxygen and nitrogen donor atoms, it was necessary to use EPR techniques to assess the coordination environment of the Cu(II) bound to MB1-2 in the presence of glycerol (required for EPR measurements) (44–47).

**Fig. 4.**
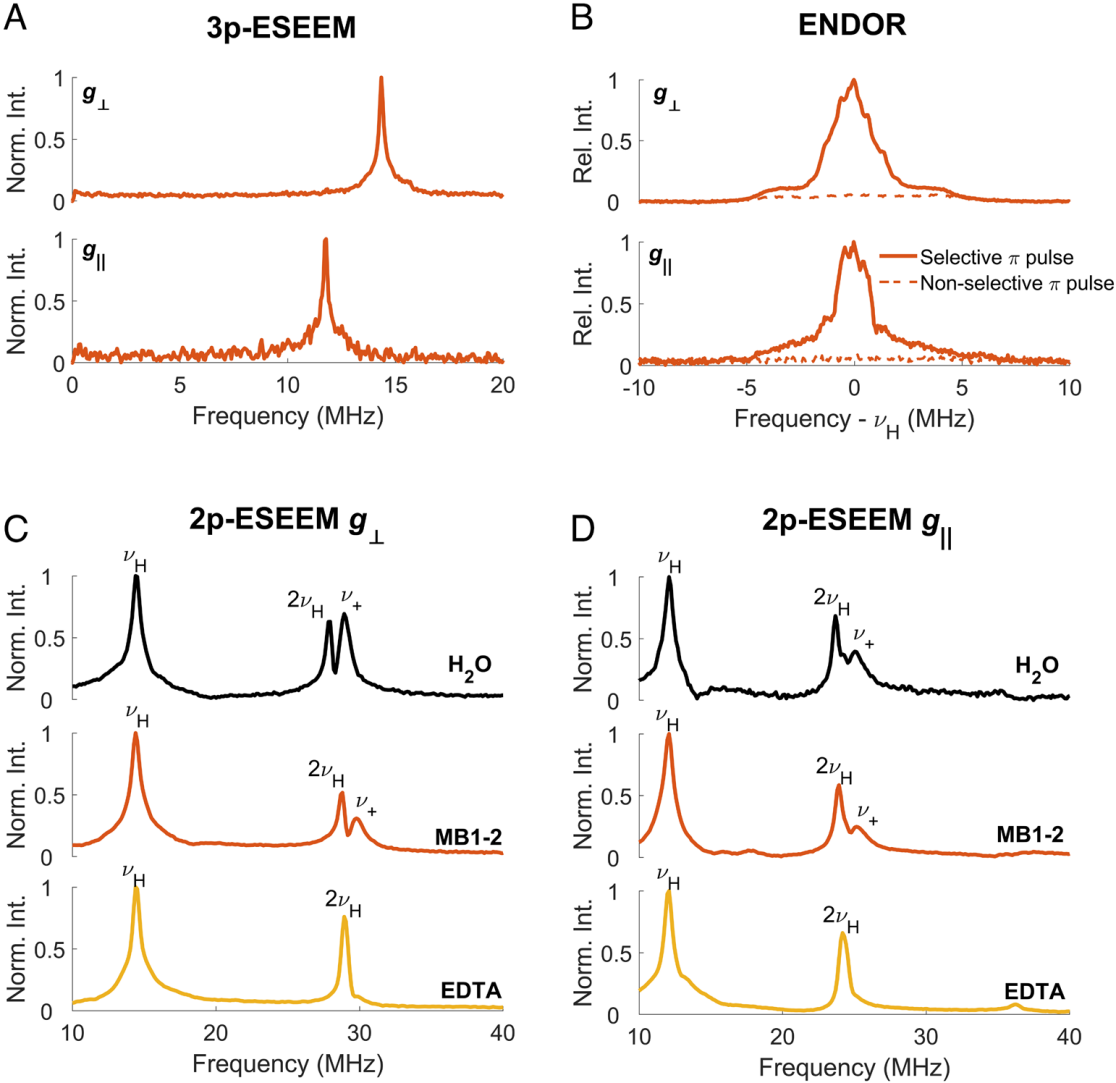
The results of EPR hyperfine spectroscopy measurements at X-band. The Larmor frequency of hydrogen nuclei is denoted as ν_H_. (*A*) Three-pulse ESEEM data in the frequency domain of MB1-2 and Cu(II) showing peaks only at ν_H_. (*B*) Davies ENDOR measured with selective (256 ns, solid lines) and nonselective (48 ns, dashed lines) pulses of MB1-2 and Cu(II). (*C* and *D*) Frequency domain spectra obtained from two-pulse ESEEM experiments for Cu(II) in water, Cu(II) and MB1-2 in buffer, and Cu(II) and EDTA in buffer. Data were collected at the extreme of the *g*_||_ (*A* and *C*) and peak of the *g*_⊥_ (*B* and *D*) orientations of the Cu(II) EPR spectrum, respectively, as shown in *SI Appendix*, Fig. S12. The shift of the sum combination peaks from hydrogen nuclei in the immediate vicinity of the Cu(II) is labeled with ν_+_.

The absorption profile in the presence of MB1-2, see *SI Appendix*, Fig. S12, is consistent with an all oxygen coordination environment (44, 48). Differences in the electron relaxation times (*T*_1_ and *T*_m_) of the Cu(II) in the presence and absence of peptide were minor but again indicated that binding had occurred (*SI Appendix*, Fig. S13). The presence or absence of ^14^N coupling can be inferred using the pulsed hyperfine EPR techniques of two- and three-pulse electron spin echo envelope modulation (2p-ESEEM, *SI Appendix*, Fig. S14*A*, 3p-ESEEM, *SI Appendix*, Fig. S14*B*) and electron nuclear double resonance (Davies ENDOR, *SI Appendix*, Fig. S15) (45, 46). Both the *g*_⊥_ and *g*_||_ orientations were probed with these pulsed methods in order to better resolve the hyperfine interactions and range of couplings around the full coordination sphere. The results are shown in Fig. 4. The three-pulse ESEEM (Fig. 4*A*) does not show any indication of weakly coupled nitrogen. Davies ENDOR data were recorded with selective and nonselective microwave inversion pulses, Fig. 4*B*. There is no evidence of strong Cu(II)–^14^N coupling and this therefore excludes the possibility of direct nitrogen binding. *SI Appendix*, Fig. S17 shows an example of the expected results for direct nitrogen binding by repeating the ENDOR experiments with Cu(II)-EDTA for comparison (46).

The two-pulse ESEEM experiment is able to detect both matrix protons and those on ligands (such as water/hydroxyl) directly bound to the Cu(II). The protons of the ligands usually have larger couplings and exhibit sum combination peaks shifted to higher frequencies. This is manifested as a splitting of the double-frequency peak of about 1 MHz in the Fourier-transformed two-pulse ESEEM. The data are shown in Fig. 4 *C* and *D* (see also *SI Appendix*, Fig. S18), with a comparison to coordinatively saturated Cu(II) bound to EDTA. The result for Cu(II) with MB1-2 indicates that protons exist both in the bulk water solution and in the immediate environment of the Cu(II) (49). Coiled coils are dynamic, nonrigid assemblies, and small molecules, such as water/hydroxides or bulkier substrates for catalysis, have previously been reported to bind to, or access, metal ion sites engineered within the hydrophobic core (34, 50–52). The hydrated Cu(II) MB1-2 complex is different from the related Tb(III) MB1-2 complex, which has previously been found to be coordinatively saturated with no waters bound (33). However, lanthanides are capable of flexible coordination geometries, and so they can capitalize on all the donor atoms presented within the designed binding site. In contrast, transition metals such as Cu(II) are more restricted in their coordination geometries and as such may be unable to fully saturate their coordination spheres with donor atoms presented by the relatively inflexible peptide-binding site, and instead could complete the coordination sphere with exogenous small-molecule ligands such as water/hydroxyl. Investigations using deuterium oxide rather than water have shown promising evidence to this assignment (*SI Appendix*, Fig. S19) (53).

### Establishing Binding Site Location.

Binding of Cu(II) and Ln(III) (33) to MB1-2 has been inferred from CD studies monitoring changes in peptide folding, and the XAS and EPR data are consistent with Cu(II) bound to the peptide in an exclusive oxygen coordination environment. But, these studies provide no information regarding binding site location. In contrast, Tb(III) binding is accompanied by sensitized emission, indicating binding in close proximity to the Trp side chain located adjacent to the designed binding site (33). To definitively demonstrate that Cu(II) binds to the same site as the Ln(III) ions, and to unambiguously locate the Ln(III)-binding site, the nitroxide spin label amino acid, 2,2,6,6-tetramethyl-N-oxyl-4-amino-4-carboxylic acid (TOAC) (54), was engineered into the 34th position, toward the C terminus, to yield MB1-2_TOAC_ (Fig. 1 and *SI Appendix*, Fig. S20 for supporting analysis). The positioning of the spin label will enable distance measurement between it and the spectroscopically orthogonal Cu(II) (and Gd(III) center) while leading to minimal disruption of the coiled coil (54).

A combination of CD and luminescence titrations demonstrates that the MB1-2_TOAC_ peptide binds Cu(II), Gd(III), and Tb(III) in the same way as MB1-2 (*SI Appendix*, Figs. S21–S24), and that introduction of the TOAC spin label does not alter the peptide folding or its subsequent coordination chemistry. The distance between the TOAC labels was measured by EPR detection of the dipolar coupling, using both the two-frequency four-pulse double electron–electron resonance (DEER, also known as PELDOR) technique (55–57) and the single-frequency relaxation–induced dipolar modulation enhancement (RIDME) (58) experiment, see *SI Appendix*, Fig. S16 for the pulse sequences. The results are shown in *SI Appendix*, Fig. S25. DEER determined the inter-TOAC distance distribution to have a most probable distance of 2.12 nm and a full-width at half-height (FWHH) on the main peak of 0.20 nm. The single predominant distance arising from our triply spin-labeled coiled coil indicates the correct symmetry, and the good agreement between the modeled and measured distance further supports our parallel three-stranded coiled coil model (Fig. 1). *SI Appendix* contains a discussion of the modulation depth of the DEER which is less than expected (though may be complicated through the multispin nature of the system) and concludes that the evidence is consistent with incomplete radical formation of the TOAC spin label.

The dipolar coupling between the bound metal ion and the TOAC labels on the peptide was measured using the DEER and RIDME experiments, and an analysis to determine distance distributions was carried out using DeerAnalysis (59). While there is precedence for using these measurements for determining the position of metal centers via strategic nitroxide spin labelling (60), to the best of our knowledge, TOAC has not previously been utilized.

**Fig. 5.**
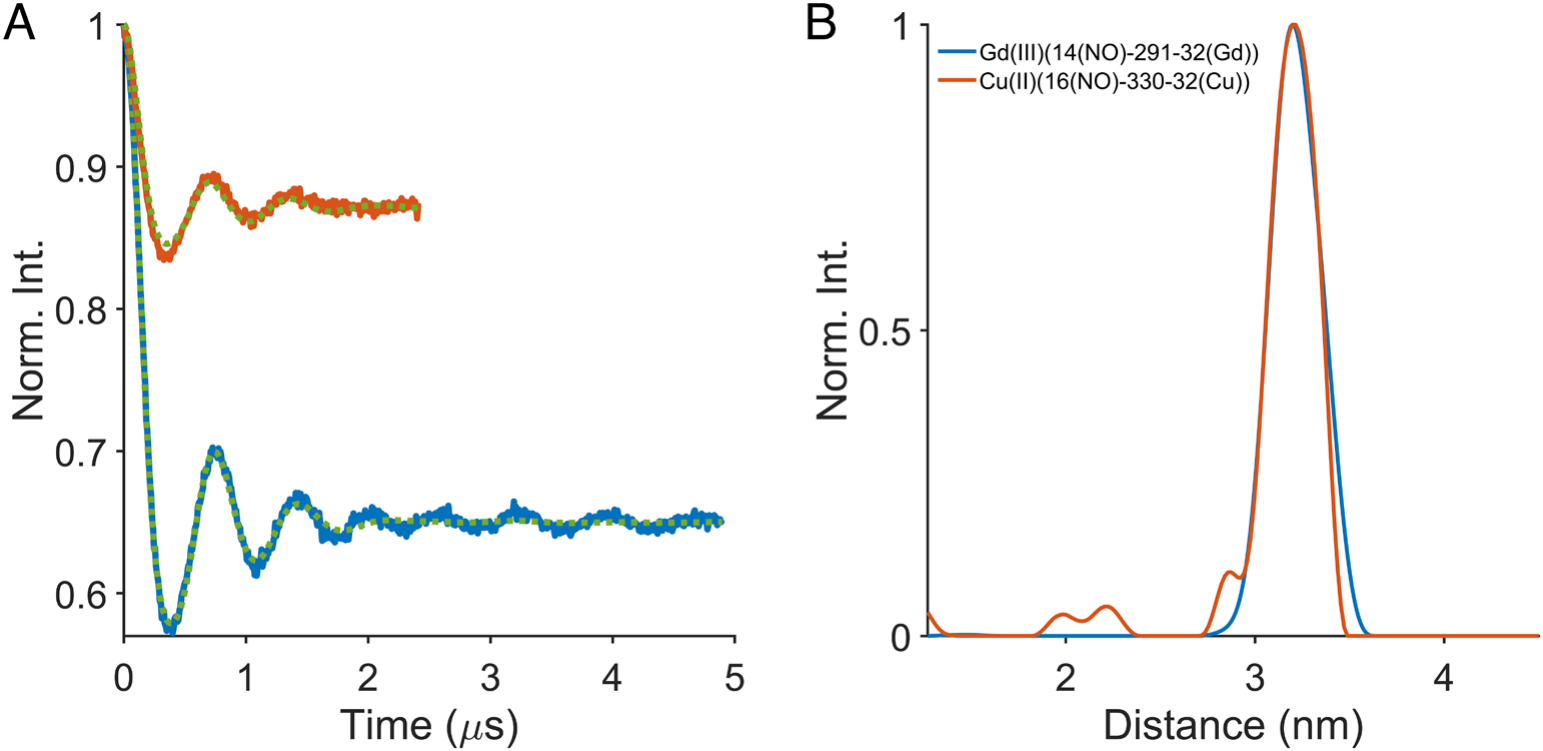
Four-pulse DEER experimental data for Gd(III) with MB1-2_TOAC_ at Q-band (blue) and Cu(II) with MB1-2_TOAC_ at X-band (red). The MB1-2_TOAC_ monomer was at three times excess to the metal and was 120 μM and 1.5 mM for the Q and X-band measurements, respectively. (*A*) The time domain data after background correction showing the distribution fit for each trace as a dashed green line. (*B*) The corresponding distance distributions. The legend panel provides the key experimental parameters for the DEER experiment, see *SI Appendix* and for validation, see *SI Appendix*, Figs. S26 and S27.

The DEER data for Gd(MB1-2_TOAC_)_3_ show a strong distinct dipolar modulation typical for a single predominant distance with a narrow distance distribution (Fig. 5*A*). The resultant distance distribution (Fig. 5*B*) has a most probable distance of 3.23 nm with an FWHH of 0.26 nm. This result is in good agreement with that approximated from our model, see Fig. 1, and the single predominant distance acts as further evidence that the symmetric coiled coil structure prevails. The narrow distance distribution is consistent with a tightly bound metal-binding site and very little coiled coil fraying toward the C terminus where the TOAC spin label is located.

The Cu(II) to TOAC DEER data were analyzed to give a distance distribution with a modal distance for the Cu(II)-bound peptide of 3.21 nm and an FWHH of 0.31 nm (Fig. 5). The broadening of the distribution with respect to the Gd(III) system may correlate to a relative wider distribution of the metal binding or it could also be a consequence of the necessarily shorter data collection in the Cu(II) case, due to relaxation, which may also have artifactual distortion (effectively damping) at the end, leading to fewer measured modulations of the DEER echo, and, therefore, a broader distance distribution. The very similar TOAC to bound-metal distances and narrow distance distributions are evidence that the Cu(II)- and Gd(III)-binding site locations are the same.

RIDME measurements of the Cu(II) and Gd(III) with MB1-2_TOAC_ were made at Q-band with results given in *SI Appendix*, Figs. S26 and S27. We found that the Cu(II)-TOAC RIDME (at Q-band) required a lot less sample than the DEER, but that the TOAC-labeled peptide needed to be present only sparsely (an MB1-2 and MB1-2_TOAC_ mixture is used) presumably due to unwanted detection of the nitroxide–nitroxide coupling (as shown in *SI Appendix*, Fig. S24) (61). The distance distributions for the Cu(II) and Gd(III) DEER and RIDME are shown in *SI Appendix*, Fig. S28. There is good agreement between the RIDME and DEER results of the two metal ions.

RIDME has been shown to be an excellent method to establish the binding of Cu(II) (62). This is because the RIDME measures the nitroxide response and is “blind” to any unbound copper (which only contributes to the background); thus, the modulation depth can be used to fit a binding curve. Though this has been shown to work well for Cu(II)–NTA labels, this measurement could not be reproduced in the MB1-2_TOAC_ system where increasing Cu(II) over peptide concentration by fivefold resulted in a reduction in the modulation depth (*SI Appendix*, Figs. S29 and S30). This is the opposite of the expected result. To investigate the origin of such an effect, we compared the ED-FS intensities for Cu(II) with and without peptide. We found that in the presence of peptide, the Cu(II) signal did not increase despite increasing its concentration in solution (*SI Appendix*, Fig. S30). We tentatively speculate that the resulting reduction in modulation depth in the RIDME signal, as the Cu(II) concentration increases (beyond 1 Cu(II) per three stranded coiled coil), may be consistent with an increasing population of bridged (diamagnetic) copper dimers within the peptide.

### MRI Relaxivity.

Having prepared a new-to-biology proteinaceous CuO_x_ site within a coiled coil, the question remained what new chemistry this could afford? Though Cu-responsive MRI contrast agents based on Gd(III) are being developed (31, 63, 64), the use of Cu(II) as a paramagnetic metal within the contrast agent has been largely disregarded as being suitable for use in MRI contrast agents, due to copper’s perceived poor relaxivity (a measure of a contrast agent’s efficiency to alter the relaxation time of bulk water) (30). However, the evidence of innersphere water/hydroxyl bound to the Cu(II), coupled with the encouraging relaxivity previously reported for the analogous Gd(MB1-2)_3_ complex (33), led us to evaluate the Cu(MB1-2)_3_ relaxivity at both 7 T and 1.4 T.

The longitudinal (*T*_1_) and transverse (*T*_2_) magnetic resonance relaxation times of water protons were measured for a solution of 40 µM CuCl_2_ in the presence of increasing equivalence of MB1-2 peptide trimer in 100 mM HEPES pH 7.0 at 300 MHz (7 T), see *SI Appendix*, Fig. S31. The addition of MB1-2 results in a decrease in relaxation time, with five equivalents of MB1-2 trimer found to be sufficient to ensure Cu(II) complexation, resulting in no further change in *T*_1_ or *T*_2_ relaxation times.

The longitudinal (*r*_1_) and transverse (*r*_2_) relaxivities of CuCl_2_, apo MB1-2, and Cu(MB1-2)_3_ were determined by plotting their relaxation rates (1/*T*_1_ and 1/*T*_2_, respectively) for each species, as a function of concentration, in 100 mM HEPES buffer at pH 7. In the case of Cu(MB1-2)_3_, solutions of CuCl_2_ were prepared in the presence of 5 equivalence of MB1-2 trimer. As there was an excess of peptide in the Cu(MB1-2)_3_ solution, the contribution of the unbound MB1-2 peptide needed to be corrected for, in the determination of the relaxivity of Cu(MB1-2)_3_. This was achieved by deducting the contribution of the excess apo-peptide from the relaxation rate of Cu(MB1-2)_3_. These corrected data reveal relaxivity values for Cu(MB1-2)_3_ (*r*_1_ = 1.3 ± 0.3 mM^−1^ s^−1^; *r*_2_ = 17.6 ± 0.7 mM^−1^ s^−1^) which are very close to those for the Gd(III) analog, Gd(MB1-2)_3_ (*r*_1_ = 5.4 ± 0.1 mM^−1^ s^−1^; *r*_2_ = 19.8 ± 0.5 mM^−1^ s^−1^) at 7 T, see Table 1 and *SI Appendix*, Figs. S32 and S33.

**Table 1.** Relaxivity data comparing Cu(MB1-2)_3_ and Gd(MB1-2)_3_

	7 T		1.4 T	
	*r*_1_/mM^−1^ s^−1^	*r*_2_/mM^−1^ s^−1^	*r*_1_/mM^−1^ s^−1^	*r*_2_/mM^−1^ s^−1^
Cu(MB1-2)_3_	1.3 ± 0.3	17.6 ± 0.7	14.2 ± 0.2	32.7 ± 0.4
Gd(MB1-2)_3_	5.4 ± 0.1	19.8 ± 0.5	15.3 ± 0.6	18.9 ± 0.1
CuCl_2_ + MB1C	nd	nd	0.6 ± 0.2	1.6 ± 0.4
CuCl_2_	0.5 ± 0.2	0.7 ± 0.4	0.6 ± 0.2	0.5 ± 0.3

Data corrected for the contribution of apo MB1-2. Errors reported are the SD of three independent repeats. nd – not determined.

The similar *r*_1_ and *r*_2_ values for the Gd(III) and Cu(II) complexes are despite the difference in spin quantum number [S = 7/2 for Gd(III) and S = 1/2 for Cu(II)] and are instead due to the different coordination chemistries of the two metal sites. The Gd(III) analog is coordinatively saturated with very little exogenous water bound and is presumed to operate primarily through an outer and second sphere mechanism (33, 34, 65). Whereas, given the evidence of bound water/hydroxyl to the Cu(II), see *SI Appendix*, Fig. S18, Cu(MB1-2)_3_ is hypothesized to operate through a combination of an outer and second sphere, similar to that present for Gd(MB1-2)_3_, and an inner sphere mechanism.

Relaxivity measurements performed at the clinically more relevant field strength of 60 MHz (1.4 T) reveal longitudinal relaxivity values (*r*_1_) that are much the same [14.2 ± 0.2 mM^−1^ s^−1^ (Cu(MB1-2)_3_); 15.3 ± 0.6 mM^−1^ s^−1^ (Gd(MB1-2)_3_)], and transverse relaxivity (*r*_2_) values that are even higher for the Cu(II) complex (*r*_2_ = 32.7 ± 0.4 mM^−1^ s^−1^) compared to the Gd(III) species (*r*_2_ = 18.9 ± 0.1 mM^−1^ s^−1^), see Table 1 and *SI Appendix*, Figs. S34 and S35. The greater *r*_2_ value for Cu(MB1-2)_3_ is likely a consequence of an important scalar contribution to *T*_2_ as observed in Mn(II) complexes (32).

To demonstrate that the high relaxivity values obtained are due to binding to the proposed oxygen site within the interior of the MB1-2 coiled coil, and not due to external nonspecific binding sites, relaxivity measurements were performed for Cu(II) in the presence of a control peptide, MB1C, which lacks the proposed metal-binding site provided by the AsnAsp layers in MB1-2 and has core hydrophobic Ile residues in their place, see Fig. 1 and *SI Appendix*, Fig. S36–S38. At 60 MHz (1.4 T), both the *r*_1_ (0.6 ± 0.2 mM^−1^ s^−1^) and *r*_2_ (1.6 ± 0.4 mM^−1^ s^−1^) values are similar to those of CuCl_2_ (*r*_1_ = 0.6 ± 0.2 mM^−1^ s^−1^ and *r*_2_ = 0.5 ± 0.3 mM^−1^ s^−1^, respectively). These findings ascertain that high relaxivity is a result of Cu(II) binding to the designed CuO_x_ site provided by MB1-2, rather than nonspecifically bound Cu(II).

Not only do the competitive relaxivity values for Cu(MB1-2)_3_ challenge the commonly held belief that Cu(II) is unsuitable for use in MRI CAs, the added advantage of Cu(II) is that ^64^Cu offers multimodal capabilities as a dual PET-MR imaging agent (66).

## Conclusions

We have successfully generated a highly elusive abiological copper site bound exclusively to oxygen donor atoms within a protein scaffold. Cu(II) binding to our designed coiled coil was probed by a range of spectroscopic techniques, including XAS and EPR, providing insight into both the coordination chemistry and binding site location. Hyperfine techniques, in particular, see a distinct lack of direct and remote nitrogen binding to Cu(II), and DEER measurements demonstrate that the Cu(II) is bound within a parallel coiled coil within the same binding site as Gd(III) by comparing distance distributions between the metal and a TOAC spin label. Despite copper largely being disregarded for use in MRI contrast agents, Cu(MB1-2)_3_ was shown to display extremely promising contrast agent capabilities, with relaxivities that are equal and superior to those of the Gd(III) analog, a metal used routinely in clinical MRI. Furthermore, imaging agents based on Cu potentially offer multimodal capabilities (i.e., PET and MRI). However, this manuscript is not advocating that these specific Cu(II) coiled coils with weak affinity be used in the clinic, rather that this class of complexes, and Cu(II) more widely, warrant further exploration in this regard.

The simplicity of the artificial coiled coil has allowed this atypical and abiological copper site to be generated within a protein scaffold, thereby achieving function and performance not normally associated with copper. Metal sites that are not part of the repertoire of biology are vital in providing metalloprotein designers with an expanded toolbox of chemistries with which to design new functional systems, such as the promising imaging capabilities reported here. This opens up applications beyond what biology is currently capable of and is the ultimate goal of metalloprotein design, and more generally synthetic biology. This work showcases some of the advantages of using simple miniature protein scaffolds as ligands into which to engineer new, and maybe currently unknown, metal-binding sites.

## Materials and Methods

Peptides were synthesized, characterized, and purified as previously reported (34). XAS measurements were recorded at the Cu K-edge absorption edge (8,979 eV) at beamline I20-Scanning at Diamond Light Source (United Kingdom). Briefly and in addition to the main text and figure captions, pulsed EPR experiments [relaxation, ED-FS, ESEEM, ENDOR (44–49, 53), DEER, and RIDME (55–62)] were performed on protonated and deuterated solutions of the peptides with 50% glycerol at X- or Q-band using a Bruker ELEXSYS E580 spectrometer operating at cryogenic temperatures (10 to 50 K). Full experimental details are provided in accompanying *SI Appendix*.

## Supplementary Material

Appendix 01 (PDF)

## Data Availability

All study data are included in the article and/or *SI Appendix*. The EPR research data supporting this publication can be accessed at https://doi.org/10.17630/835ab42b-5999-4437-8ccb-34cdc630a179 (67).
