# Supplementary material for: Design of the elusive proteinaceous oxygen donor copper site suggests a promising future for copper for MRI contrast agents
Source: Proc Natl Acad Sci U S A. 2023 Jun 26;120(27):e2219036120. doi: 10.1073/pnas.2219036120 (PMC10318980; doi:10.1073/pnas.2219036120)
Supplement: Supplementary file 1 — Appendix 01 (PDF) [file pnas.2219036120.sapp.pdf]

PNAS

Supporting Information for

Design of the elusive proteinaceous oxygen donor copper site suggests a promising future for copper for MRI contrast agents

Anokhi Shah,[a,b†] Michael J. Taylor,[a†] Giulia Molinaro,[c†] Sellamuthu Anbu,[c] Margaux Verdu,[c] Lucy Jennings,[c] Iuliia Mikulska,[d] Sofia Diaz-Moreno,[d] Hassane EL Mkami,[a] Graham M. Smith,[a] Melanie M. Britton,[c] Janet E. Lovett[a,b]* and Anna F. A. Peacock[c]*

[a] SUPA School of Physics and Astronomy, University of St Andrews, KY16 9SS, UK
[b] BSRC, University of St Andrews, KY16 9ST, UK
[c] School of Chemistry, University of Birmingham, Edgbaston, B15 2TT, UK
[d] Diamond Light Source, Harwell Science and Innovation Campus, Didcot, Oxfordshire, OX11 0DE, UK

* jel20@st-andrews.ac.uk
* a.f.a.peacock@bham.ac.uk

† AS, MJT and GM contributed equally to this work

This PDF file includes:

Supporting text
Figures S1 to S38
Table S1
SI References

Contents:

1. Materials and Methods
2. Impact of glycerol on Cu(II) binding to MB1-2
3. Figure S1 – Computational model of Cu(MB1-2)$_3$
4. Figure S2 – Fluoresence Cu(II) titration
5. Figure S3 – CD Cu(II) titration at different MB1-2 concentrations
6. Figure S4 – Job's plot analysis
7. Figure S5 – Kinetics of binding
8. Figure S6 – CD thermal unfolding
9. Figure S7 – Experimental EXAFS signals and FT magnitude and best fits
10. Table S1 – EXAFS best fit parameters
11. Figure S8 – XANES spectra of $CuCl_2$ +/- glycerol and of $CuCl_2$ & MB1-2 +/- glycerol
12. Figure S9 – CD MB1-2 +/- glycerol, +/- $CuCl_2$, +/- $GdCl_3$.
13. Figure S10 – CD Cu(II) MB1-2 titration +/- glycerol
14. Figure S11 – Linear combination fit of $CuCl_2$ in the presence of MB1-2
15. Figure S12 – ED-FS EPR spectrum of Cu(II) with MB1-2
16. Figure S13 – Echo decay/inversion recovery plots for Cu(II) with MB1-2
17. Figure S14 – Electron spin echo envelope modulation (ESEEM) pulse sequences
18. Figure S15 – Davies Electron Nuclear Double Resonance (ENDOR) pulse sequence
19. Figure S16 – Four-pulse DEER and the five-pulse RIDME pulse sequence
20. Figure S17 – Cu(II) EDTA Davies ENDOR
21. Figure S18 – Two-pulse ESEEM data for Cu(II) with MB1-2
22. Figure S19.i – Three-pulse ESEEM data of Cu(II) with MB1-2 and water counting
    Figure S19.ii – Three-pulse ESEEM data of Cu(II) with MB1-2 and water counting
23. Figure S20 – HPLC and mass spectrum for MB1-2_{TOAC}
24. Figure S21 – CD Cu(II) MB1-2_{TOAC} titration
25. Figure S22 – CD Gd(III) MB1-2_{TOAC} titration
26. Figure S23 – CD Tb(III) MB1-2_{TOAC} titration
27. Figure S24 – Luminescence Tb(III) MB1-2_{TOAC} titration
28. Figure S25 – Nitroxide-Nitroxide 4p-DEER and 5p-RIDME with validation
29. Figure S26 – Gd(III) MB1-2_{TOAC} DEER and F-L/Sp-L RIDME
30. Figure S27 – Cu(II) MB1-2_{TOAC} DEER and F-L/Sp-L RIDME
31. Figure S28 – Comparative distributions of Cu(II) and Gd(III) DEER/RIDME
32. Figure S29 – 2D RIDME modulation depth vs mixing time for Cu(II) bound to MB1-2
33. Figure S30 – ED-FS Pseudotitration of Cu(II) bound to MB1-2
34. Figure S31 – NMR T_1 and T_2 plots of Cu as a function of increasing MB1-2
35. Figure S32 – 300 MHz NMR T_1 and T_2 relaxivity plots of Cu(MB1-2)$_3$ at pH 7
36. Figure S33 – 300 MHz NMR T_1 and T_2 relaxivity plots of Cu(MB1-2)$_3$
37. Figure S34 – 60 MHz NMR T_1 and T_2 relaxivity plots of Cu(MB1-2)$_3$
38. Figure S35 – 60 MHz NMR T_1 and T_2 relaxivity plots of Gd(MB1-2)$_3$
39. Figure S36 – HPLC and mass spectrum for MB1C
40. Figure S37 – CD Cu(II) MB1C titration
41. Figure S38 – 60 MHz NMR T_1 and T_2 relaxivity plots of $CuCl_2$ + $MB1C_3$
42. References

1. Materials and Methods

Materials: Chemicals were used as received and purchased from; Sigma Aldrich (diethyl ether, gadolinium chloride hexahydrate, copper chloride dihydrate), Pepceuticals (Fmoc protected amino acids, HBTU (O-benzotriazole-N,N,N',N'-tetramethyluronium-hexafluorophosphate), synthesis grade DMF (dimethylformamide) and 20% piperidine in DMF premix), AGTC Bioproducts Ltd. (rink amide MBHA resin, NMP (N-methyl-2-pyrrolidone), DIEA (N,N-diisopropylethylamine) and DCM (dichloromethane)), Acros Organics (xylenol orange sodium salt, acetic anhydride, TIPS (triisopropylsilane) and TFA (trifluoroacetic acid)), Rathburn Chemicals Ltd. (>99.9% DMF), Merck (Fmoc-TOAC-OH (2,2,6,6-tetramethylpiperidine-N-oxyl-4-(9-fluorenylmethyloxycarbonyl-amino)-4-carboxylic acid**),** glycerol) and Fisher Scientific Ltd. (HPLC (high pressure liquid chromatography) grade water and acetonitrile, HEPES (2-[4-(2-hydroxyethyl)piperazin-1-yl]ethanesulfonic acid), urea, EDTA (ethylenediaminetetraacetic acid) and glacial acetic acid). Despite generally being considered to be a "good" buffer, HEPES has been reported to show mild binding affinity to Cu(II).(1, 2)

Peptide Synthesis, Characterization and Purification: Peptides MB1-2 and MB1C were synthesized, purified and characterized as reported previously.(3) The TOAC labelled derivative, MB1-2_{TOAC}, was synthesized on rink amide MBHA resin (0.1 mmol scale) and the first two residues were double coupled manually using standard Fmoc-amino acid solid-phase peptide synthesis protocols,(4) with manual couplings monitored by the Kaiser test.(5) Onto this was coupled Fmoc-TOAC-OH (0.250 g, 0.06 mmol) for 20 hrs (1st coupling), 10 hrs (2nd coupling) and 4 hrs (3rd and final coupling), respectively. A diazabicyclo[5.4.0]undec-7-ene (DBU) solution (1 mL DBU + 10 mL piperidine + 39 mL NMP) was used to deprotect the low nucleophilic amine TOAC group. The following amino acid, Glu, was triple coupled manually, but the remainder of the sequence was assembled on the automated CEM Liberty Blue peptide synthesizer, as previously reported.(3) In order to generate the N-Ȯ radical, the peptide was incubated with 10% ammonia solution for 2 hrs.

Models: The cartoon models presented in Figure 1 were generated in PyMOL (v. 1.4 Schrödinger, LLC) based on manipulation of 2W6B(6) through amino acid deletions and side chain mutagenesis. The TOAC spin label was added using ALLNOX.(7, 8) From the generated model the distance between the metal and the nitroxide radical of the TOAC spin label was measured to be 3.49 nm with a standard deviation of 0.02 nm. The inter-TOAC distances were measured as being 2.09 nm with a standard deviation of 0.09 nm. The standard deviation originates from assessing distances separately for the N and the O of the nitroxide.

The computational model of copper bound to the MB1-2 peptide trimer (Figure S1), was generated by applying ColabFold(9) to MB1-2, and subjecting the resulting structure to Metal3D,(10) to identify copper binding site(s).

UV-visible spectroscopy for peptide concentration determination: UV-visible spectra were recorded on a Shimadzu 1800 UV Spectrophotometer, recorded in single beam mode with a medium scan speed, slit width of 1.0 nm, a data interval of 1.0 nm and scan range 420-260 nm (Trp), using a 1 cm pathlength, 700 μL quartz cuvette. For the preparation of the EPR samples, these were recorded on an Eppendorf BioSpectrometer basic, using single beam absorption with a Xenon flash lamp across 200-830 nm wavelength range, a data interval of 1.0 nm and using a 1 mm pathlength μCuvette.

Stock solution Preparation: The peptides used in this work feature a single Trp chromophore to allow for accurate concentration determination. The concentration of freshly prepared peptide stock solutions in MilliQ water, were deduced from the absorption of the single Trp residue at 280 nm (ε_{280} = 5690 M^{-1} cm^{-1}) in 7 M aqueous urea, performed in triplicate for accuracy and left for 10 mins prior to quantitative UV determination.

$CuCl_2$ stock solutions were freshly prepared in volumetric glassware, to yield a concentrated stock (100 mM) in either water or deuterium oxide, and subsequently diluted where necessary. The concentrations of freshly prepared stock solutions of $TbCl_3$, $GdCl_3$ (~1 mM) in MilliQ water, were determined in triplicate using a xylenol orange indicator and EDTA titration with

Ln^{3+} standard solutions, following a procedure previously reported by Fedeli and co-workers.(11)

Circular Dichroism: CD spectra were recorded on a Jasco J-715 Spectropolarimeter in a 1 mm pathlength quartz cuvette for 30 μM peptide monomer solutions. The optical chamber was purged with nitrogen and kept under a nitrogen atmosphere throughout the experiments. Stock solutions (1 mM or 10 mM) of $CuCl_2$/$GdCl_3$/$TbCl_3$ were titrated into 30 μM peptide monomer in 10 mM HEPES buffer pH 7.0. $CuCl_2$ titrations were also performed into solutions of MB1-2 at lower (17 μM) and higher (30, 90, 120 and 150 μM) monomer concentrations in 10 mM HEPES buffer pH 7. A 30 μM MB1-2 monomer solution in 10 mM HEPES buffer pH 7.0, was recorded in the absence and presence of 50% glycerol, and $CuCl_2$ or $GdCl_3$, respectively. All solutions were left to equilibrate for 10 minutes before recording spectra, and the observed ellipticity converted into molar ellipticity, with the helical content calculated as the percentage folded, based on the theoretical maximum ellipticity as reported by Scholtz et al.(12)

Thermal unfolding data was recorded by monitoring the ellipticity at 222 nm of a 30 μM solution of MB1-2 peptide monomer in 10 mM HEPES buffer pH 7.0 in the absence and presence of 100 μM $CuCl_2$, using a Jasco Peltier Type PTC-4235 temperature controller ramping from 10 °C to 80 °C.

Fluorescence Spectroscopy: Emission spectra were recorded in a 1 cm pathlength quartz cuvette on a Jasco FP-8500 fluorescence spectrometer. Aliquots of a 1 mM stock solution of $CuCl_2$ were titrated into a 30-32 μM solution of MB1-2 peptide monomer in 10 mM HEPES buffer pH 7.0 and the emission profile recorded after 15 minutes equilibration. Solutions were excited at 280 nm, the emission was scanned and integrated from 300-455 nm and normalised. The normalised emission intensities were corrected for the dilution and plotted as a function of $CuCl_2$ equivalents.

X-ray Absorption Spectroscopy (XAS): XAS measurements were recorded at the Cu K-edge absorption edge (8979 eV) at beamline I20-Scanning at Diamond Light Source (United

Kingdom).(13) The beamline was equipped with a four-bounce scanning Si(111) monochromator,(14) and the rejection of higher harmonics was achieved by using two dedicated rhodium coated mirrors operating at 5 mrad incidence angle. Extended X-ray absorption fine structure (EXAFS) spectra were collected in fluorescence mode. A 30 cm long ionization chamber filled with the appropriate mixture of He and Ar gases to absorb 20% of the X-ray beam was used as the incident intensity monitor. A Canberra 64-pixel Monolithic Segmented Hyper Pure Germanium Detector (HPGe) partnered with the Xspress4 digital pulse processor(15) was used to measure the fluorescence signal. Energy calibration was achieved by the simultaneous measurement of a 5μm-thick Cu metal foil.

To avoid photoreduction of Cu(II) the X-ray beam delivered by the beamline was attenuated by using a 6 mm thick carbon filter. In addition, the samples were measured at cryogenic temperatures to reduce damage incurred by the X-ray beam. They were loaded in polyester capillaries (MiTeGen, LLC) and frozen rapidly by submersing them in liquid nitrogen. A LN_2 cryoject (Oxford Instrument) was used during the measurements to keep the samples at cryogenic temperatures.

Samples containing 1 mM $CuCl_2$ in 50 mM HEPES buffer pH 7.0 were prepared in the absence and presence of 6.6 mM MB1-2 peptide monomer. The high concentration of the peptide drives complexation and avoid complications from unbound copper. The samples were prepared with and without 50% glycerol, so the effect of the glassy agent in the copper complexation could be determined.

The EXAFS data analysis was performed by using the Demeter software(16) following standard procedures. The quantitative analysis of the first peak in the magnitude of the Fourier transform for the copper sample in the presence of MB1-2 peptide monomer and glycerol was performed by fitting it to a set of theoretical scattering paths derive from four different CuO_x structures. Trigonal planar (coordination 3), trigonal pyramidal (coordination 4), square pyramidal (coordination 5) and Jahn-Teller distorted octahedral (coordination 6) geometries were considered as input structures for the fitting procedure. The theoretical amplitudes and phases of the scattering paths derived from those structures were calculated by the FEFF-6

code.(17) The EXAFS fitting was performed in R-space (1-2 Å) using the Fourier transformed in the k range 3.7-11 Å^{-1}.

For the trigonal planar model, the number of oxygens was allowed to vary during the fitting procedure, and the best fit was obtained with N_O=2.7 ± 0.6. In the cases of the trigonal pyramidal, square pyramidal and Jahn-Teller elongated octahedral models, the oxygen coordination number was fixed to 4, 5 and 6 respectively. For all models considered, the amplitude reduction factor (S_0^2) was fixed to 0.91, as determined from the fitting of the Cu metal foil. The distance between the copper and the oxygen atoms (R), the Debye-Waller factors (σ^2) and the inner potential correction (ΔE_0) were allowed to vary in the fit for each model. The same Debye-Waller factor value was used for the axial and the planar atoms in the trigonal pyramidal, the square pyramidal and the Jahn-Teller elongated octahedral models. The best-fit parameters from the fit of the EXAFS signal are shown in Table S1.

EPR: In summary, and generally: the Cu(II) and Gd(III) EPR measurements were taken in the presence of three equivalents of MB1-2 monomer (or MB1-2_{TOAC} monomer either exclusively or at a 1:50 dilution with MB1-2 peptide) unless stated in the figure captions. The components were always mixed in the same order (water or deuterium oxide, HEPES solution in water or deuterium oxide, peptide, metal) and allowed to incubate for 30 min at room temperature. Following incubation, glycerol or glycerol-d_8 was added to give 50% by volume, and the samples were loaded into EPR tubes and then flash-frozen using liquid nitrogen.

In more detail: MB1-2 samples were prepared either in protonated or deuterated solvent using H_2O/D_2O and glycerol/glycerol-d_8 at 50% sample volume for cryogenic measurements with protonated/deuterated HEPES used as the buffering agent to control sample pH. Buffers used in the PDS measurements were prepared from 0.1 M stock (pH 7.0 at room temperature) to produce a final buffer concentration of 10 mM. The MB1-2 peptide was found to be acidic and not well buffered so subsequent measurements were buffered using a 1 M HEPES stock (pH 7.1 at room temperature) to give a final buffer concentration of 100 mM. The pH measurements of the samples were recorded using a Fisherbrand pH meter and Mettler Toledo InLab Micro pH electrode and measured after the addition of glycerol. This includes samples

measured in D_2O, and the values are reported without correcting for deuterium. The results presented in this work for the 500 μM ED-FS, relaxation, 2p-ESEEM, 3p-ESEEM and ENDOR measurements were performed on a single sample for each system with pH values of 7.0, 6.9 and 6.9 for the Cu(II) in buffer, MB1-2 and EDTA measurements respectively. The other ED-FS samples with less Cu(II) had a pH of 6.9. The 3p-ESEEM hydration measurement samples also had a pH of 6.9. The PDS samples' pH were assessed post measurement from stored samples. For the DEER samples, the Gd(III)-MB1-2 was measured as pH 6.8 (this is also the so-called fully labelled sample used in RIDME) whereas the more concentrated Cu(II)-MB1-2 had a pH 5.1. The Gd(III) RIDME samples were pH 6.7 for the Gd(III) with MB1-2 and pH 5.6 for the Cu(II) with MB1-2 where the MB1-2 was sparsely labelled (Sp-L, 50:1 MB1-2:MB1-2_{TOAC}). The Cu(II) with MB1-2_{TOAC} (so-called fully-labelled with short name F-L) was at pH 6.8. The origin of the difference in pH between the fully and sparsely TOAC labelled sample is that the overall monomer peptide concentration is increased from 150 μM to 600 μM from full MB1-2_{TOAC} to doped sample.

All measurements were performed on a Bruker ELEXSYS E580 spectrometer equipped with a Cryogen-Cree Variable Temperature Cryostat (CF-VTC) from Cryogenic Ltd. In general the PDS measurements were obtained at Q-band, with the exception of the copper-nitroxide DEER which was at X-band. The X-band DEER measurements were performed with a 1 kW TWT amplifier and MS3 spit-ring resonator with 60 μL sample volume. The Q-band measurements were performed with a 150 W TWT amplifier and EN 5106QT-2w cylindrical resonator with 60 μL sample volume. The Q-factor of the resonator remained low and approximately the same for DEER and RIDME experiments. The hyperfine measurements were at X-band. The hyperfine measurements (and ED-FS not associated with PDS measurements) utilized an MD4 resonator using 120 μL sample volume with the exception of the 3p-ESEEM hydration (water counting) study where the measurements used the MS3 spin-ring resonator.

Echo-detected field sweep (ED-FS) were taken with the π/2-τ-π and echo detection. Concentrations are given in the figure captions. ED-FS in Figure S12 were taken at 10 K with a π pulse length was 32 ns and τ of 200ns. The intensity of the ED-FS increases when the MB1-

2 is present, which may indicate that there is more monomeric Cu(II) present in this case. ED-FS are also provided for the PDS data at X (for copper-nitroxide DEER) or Q-band (Figures S25, S26, S27 and S30). These were measured at 50 K and 25 K for the nitroxide (π pulse length was 32 ns and τ of 400 ns). At 10 K Gd(III) and at 15 K for the and Cu(II) DEER (π pulse length was 32 ns and τ of 400 ns). The ED-FS pseudo titration RIDME shown in Figure S27 was taken at 20 K (π pulse length was 32 ns and τ of 200 ns). The shot repetition time (SRT) was optimised for detection signal-to-noise (and to allow for efficient averaging) which generally meant detecting about 70% of the maximum intensity of the echo. For Figure S12 and S27 this meant a SRT of 3 ms and 1 ms for 10 K and 25 K. The PDS measurements shown in Figures S25-27 include inserts of the ED-FS for the systems, where the metal and nitroxide field sweeps are optimised separately due to the different relaxation times. Here the nitroxide was measured with a 3 ms SRT at 50 K and 15 ms at 25 K, and the Cu(II) and Gd(III) were measured with a SRT of 500 μs and 1 ms, at 15 K and 10 K, respectively.

T_1 and T_m relaxation measurements were taken with a Cu(II) concentration of 500 μM. The pulse sequences were π-τ-π/2-T-π and π/2-τ-π, respectively, with the inter-pulse delay τ stepped and the π pulse length was 32 ns. The resulting echo integration provides an intensity plot against time which contains the relaxation dependant signal decay (Figure S13).

The ESEEM signal for probing remote (weakly coupled) ^{14}N is expected to be particularly prominent at X-band EPR frequency due to the meeting of the exact cancellation conditions. In such conditions, the nuclear Zeeman and hyperfine interactions of the ^{14}N effectively cancel in one electron spin-manifold and this would be expected to give an ESEEM spectrum with distinct ^{14}N nuclear quadrupole interaction frequencies spread over the range 0-10 MHz. The 3p-ESEEM experiment has the advantage over 2p-ESEEM of measuring a simpler spectrum. However, an analysis of the sum combination peaks in 2p-ESEEM can provide a deeper understanding of the system by clarifying couplings arising from ligand protons. Both are used here to gain insight into the binding environment of the Cu(II) in the MB1-2 coiled coil. ESEEM (pulse sequences in Figure S14, the 2p-ESEEM sequence is the same as the sequence used to measure T_m) measures echo modulations in the time domain while stepping τ or T in two- or three-pulse ESEEM respectively, and the associated raw data is then Fourier

transformed into the corresponding nuclear frequencies in the frequency domain. Prior to Fourier transformation, the data is first background corrected through the fitting and then removal of a bi- or stretched- exponential function. The corrected data is then processed using a HAMMING window, zero filled to 1024 points in the x-axis and then transformed by Fast-Fourier Transform (FFT). All ESEEM data were analyzed using MATLAB2020b using in house code for the hyperfine analysis, following established data processing techniques.

Two-pulse ESEEM measurements were made with 500 μM Cu(II) concentration at two orientations $g_\perp$ & $g_{||}$ within the Cu(II) spectrum. The two-pulse ESEEM experiments used a π pulse length of 32 ns, and data were recorded with two-step phase cycling and an initial inter-pulse delay of τ = 120 ns.

Two-pulse ESEEM (Figure 4 and S18) for Cu(II) in water, with MB1-2 or with EDTA present shows signals centred at the proton Larmor frequency as well as the double frequency. The splitting at the double frequency arises from the slight difference in coupling between the inner and outer sphere water protons coupled to the paramagnetic center. The amplitude of the sum combination peak is reduced from moving from water to the MB1-2 environment and is completely absent in the EDTA chelating agent, which should completely isolate the inner sphere of the paramagnetic center. This allows for the assignment of the second double peak to inner sphere protons, which we tentatively assign to water. When integrating the peak intensity of the inner sphere peak of the perpendicular orientation protons of MB1-2 and comparing it to the Cu(II) in water, it was found that the MB1-2 peak's intensity was 55% of the integrated intensity of the free Cu(II). Integration was performed by choosing the local minima separating the sum-combination and double peak as the low-field cut off and using the corresponding field position at the high-field side of the peak as the max field cut-off. This way the x-axis field length sampling for calculating the area was consistent across the H_2O and MB1-2 samples to make the comparison as fair as possible when integrating under the curves.

Three-pulse ESEEM was measured for various inter-pulse lengths to ensure that all blind spots were accounted for, and no information is lost. The observed peaks shown in Figure 4a were recorded at both the $g_\perp$ and $g_{||}$ orientations are centered at the Larmor frequency of protons which correspond to distant coupled protons present from the bulk solution. There is no

indication of weakly coupled nitrogen. Three-pulse ESEEM experiments for the skyline experiment used τ_1, τ_2= 120 ns (+16 ns step/scan), T = 400 ns with a 500 μM Cu(II) concentration. Three-pulse ESEEM experiments for hydration studies were set up with the initial inter-pulse delays τ_1, τ_2, T = 148 ns. These samples had 100 μM Cu(II) concentration and fifteen equivalents of MB1-2 monomer or five equivalents of EDTA at X-band (ν ~ 9.24 GHz) in 10 mM HEPES from 100 mM pH 7.0 stock in 50% glycerol as cryoprotectant in either H_2O or D_2O. The final sample volume was 60 μL. In order to more accurately process the data, two distinct orientations have been selected at the same field for each sample, 270 mT and 315 mT corresponding to out-of and in-plane respectively. This ensures that the observed nuclear Larmor frequencies for the coupled nuclei are the same for each sample in a given orientation, improving the analysis. Experiments were performed at the blind spot for protons(τ = 148 ns for 315 mT and 174 ns for 270 mT) to remove any contributions from the weakly coupled protons

The water counting 3p-ESEEM data (Figures S19.i/.ii) were analyzed based on using similar methods as Hoogstraten and Britt.(18) The ESEEM spectrum of Cu(II) in D_2O was divided by an ESEEM spectrum of an identical sample in H_2O (see associated spectra in Figures S19.i/.ii a) to remove contributions from nonexchangeable protons, and the resultant quotient spectrum (Figures S19.i/.ii b) shows only modulations related to solvent exchangeable deuterons. However, the latter arise either from contributions of the first or second-coordination sphere. To only keep signals from the first- coordination sphere (Figures S19.i/.ii g), the resultant quotient was subsequently divided by the quotient spectrum of Cu(II) in EDTA in D_2O and H_2O (Figures S19.i/.ii c), which has only modulations arising from the second-coordination sphere. This procedure led to the spectrum shown in Figures S19.i/.ii g which consists of only modulation form inner-sphere coordinated deuterons. Similar procedures were applied to Cu(II) in MB1-2 and the spectra resulting from described steps are shown respectively in Figure S19.i/.ii e,f and g. The ESEEM signal scales as the product of the number of like coupled nuclei, thus it was possible to power scale the ESEEM spectrum of the in-plane (Figure S19.i) four-coordinated water, and two-coordinate out-of-plane coordinated water (S19.ii) in the aqueous Cu(II) (Figures S19.i/.ii h) and to plot ESEEM spectra with modulation depths

corresponding to the expected bound D_2O. In each case the first 1 μs of the time domain data is highlighted more clearly show the coordinated assignment from the observed deuterium oxide modulation depth (Figures S19.i/.ii i). These results are evidence there are two directly bound water molecules present in the plane of the Cu(II) MB1-2 complex (Figure S19.i i), and little evidence of bound water in the out-of-plane orientation (Figure S19.ii i).

Davies ENDOR is best suited to measure strong (directly coordinated) ^{14}N coupling. It also offers the ability to selectively suppress signals from overlapping weakly and strongly coupled nuclei. The combination of 2p-, 3p- and ENDOR can therefore establish whether nitrogen is bound to the Cu(II). Davies ENDOR measurements (Figure S15) used a sample with 500 μM Cu(II) concentration at X-band (ν ~ 9.86 GHz) at an observation field of 338.3 mT and 277.7 mT for the $g_{\perp}$ & $g_{||}$ orientations respectively. The RF π pulses were optimized by nutation experiments and were found to be 12 μs. The microwave frequency selective π pulses were 256 ns length, and non-selective π pulses were a length of 48 ns. The other parameters in Figure S17 were τ = 680 ns and T = 16 μs. The radiofrequency was swept from 0 to 30 MHz in step of 1 MHz. The associated spectra are shown in Figure 4b and are centered around the proton Larmor frequency. A non-selective 48 ns inversion pulse was used to suppress the signal from weakly coupled protons and to probe the presence of strongly coupled nuclei such as nitrogen, whose peaks would be expected to be centered a few MHz offset from the proton Larmor frequency. There is no evidence of strong Cu(II)-^{14}N coupling and this therefore excludes the possibility of direct nitrogen binding. Figure S17 shows an example of the expected results for direct nitrogen binding by repeating the ENDOR experiments with Cu(II)-EDTA for comparison.

The pulse sequences for DEER and RIDME are shown in Figure S16. Q-band PDS measurements were preferred where possible due to much higher sensitivity, but TOAC-Cu(II) DEER measurements were limited by the bandwidth of the resonator at Q-band and so X-band was used. 2-Step phase cycling and 8-step phase cycling was employed for the 4p-DEER and 5p-RIDME experiments respectively, with τ averaging to avoid distortions and complications due to hyperfine modulations from protons and deuterons, which can be quite prominent in the time traces. The RIDME sequence had 3 τ values (40 ns step) for both π/2 - π sub-pulse-

sequences resulting in a total of 72 component traces for each complete scan. For the DEER this was set as 5 τ (24 ns step) values for Q-band, and 8 τ values (56 ns step) at X-band. These are typical experimental values used for τ averaging to remove deuterium modulation (ESEEM) effects during the data acquisition. Averages were taken until sufficient signal-to-noise was given.

DEER was performed at 15 K for Cu(II)-TOAC (concentration of Cu(II) 500 μM) with observation pulses on the copper and pump on the maximum of the nitroxide, 10 K for Gd(III) (concentration of Gd(III) 40 μM) with pump pulse on the nitroxide maximum and observer on the Gd(III), and 50 K for TOAC-TOAC (monomer concentration 150 μM) with pump pulse on the maximum of the nitroxide. All observer pulses in DEER are optimized to the same length. The key DEER variables are given in the figure legends (S25, S26 and S27) as [pump length in ns (paramagnetic center position) - frequency separation (MHz) - observation pulse length in ns (paramagnetic center position)].

The DEER modulation depth provides information on the fractional number of coupled spins. The TOAC-TOAC DEER (Figure S25) shows a modulation depth of only ~17% (as defined by DeerAnalysis), which is less than expected for three-coupled nitroxides.(19) Using equation 2 in Hilger et al (20) and assuming only pairwise interactions with a theoretical maximum modulation depth of 30% (which is based on usual observed values, though we note that for our measurements here our pump pulse was optimised at 20 ns rather than the more standard 14 or 16 ns) the number of labels per coiled coil is estimated to be 1.5 rather than 3. Repeating this calculation for the Gd(III)-MB1-2_{TOAC} DEER modulation depth (35% from DeerAnalysis), where the nitroxide was the pumped species and the theoretical pair-wise modulation depth of 30 % was again assumed, provided an estimate of label content of 1.2 per coiled coil. However, we note that modulation depth in multi-spin systems is often not what one might expect – even when fully spin labelled. Inspection of ED-FS data taken with Cu(III)-MB1-2_{TOAC} or Gd(III)- MB1-2_{TOAC} samples to assess the area under the absorption profiles of the two paramagnetic species (and noting that the precision of this will suffer from various relaxation effects, which will be detrimental to accuracy) were consistent with approximately 1.3 labels per trimer. There are many assumptions being made in these various assessments,

but they are fairly self-consistent and it can therefore be concluded that the peptides do not have full incorporation of the spin-active TOAC. Analysis of the Cu(II)-MB1-2_{TOAC} modulation depth (13% from DeerAnalysis) is complicated by possible orientation effects and not knowing the expected modulation depth for the used bandwidth of the resonator.(21) However, the result may indicate that there is incomplete binding of Cu(II) under the measurement conditions used, and hence RIDME was utilised to investigate further.

RIDME is a single frequency experiment so all pulses could be optimized at the center of the cavity response (ν ~ 34.01 GHz) with observer π pulses of 32 ns, set at the peak of the nitroxide ED-FS signal to give the best echo intensity. They enable a smaller concentration of Cu(II) and peptide than the X-band DEER and also provide a method to reduce any possible orientation selectively that might have been present in the Cu(II)-nitroxide DEER measurements. However, the RIDME measurements are complicated for Gd(II)-TOAC through the presence of harmonics of the dipolar coupling being present (from the high spin nature of the Gd(III)).

Nitroxide-Nitroxide RIDME was performed at 25 K with an 80 μs mixing time (T_{mix}) and an MB1-2_{TOAC} concentration of 150 μM. The temperature and mixing time were not optimised as the aim was to ascertain if any apparent dipolar couplings could be obtained from observing on the nitroxide, under the similar spectrometer conditions as the Cu(II) bound sample, without any paramagnetic metals present. The result of which could help explain the apparent short distance distortions that were present in the fully labelled peptide data (Figure S25).

RIDME in Figures S26-27 used T_{mix} of 80 μs which gave a good modulation depth and signal-to-noise. The operating temperatures were set at 25 K for Cu(II) and 20 K for Gd(III) corresponding to the optimum T_1 relaxation times for both the paramagnetic metal ions, respectively. Gd(III) with three equivalents of MB1-2_{TOAC} monomer (referred to as fully-labelled, F-L) had a Gd(III) concentration of 40 μM whereas the sparsely-labelled (Sp-L) sample with a ratio of 1:50 MB1-2_{TOAC}:MB1-2 monomer had an overall three equivalents of peptide and a Gd(III) concentration of 100 μM. F-L Cu(II) with MB1-2 contained 50 μM metal ion and the Sp-L sample contained 200 μM Cu(II).

To investigate the modulation depth trend with T_{mix} for Cu(II)-peptide, a 2D RIDME experiment was used where the length of the mixing block was increased (Figure S29). The modulation depth was then obtained from the DEERAnalysis software after background removal, and then fitted to give an approximation of the modulation depth trend for increasing mixing time (Figure S29e). To investigate the effect of increased copper concentration to the dipolar signal, samples of 150 µM monomer (Sp-L, 1:50 MB1-2_{TOAC}:MB1-2) at two concentration ratios of Cu(II): 50 µM (1:3 Cu(II):peptide) and 250 µM (5:3 Cu(II):peptide) were measured by RIDME (Figures S29/S30). A RIDME experiment was then measured with T_{mix} = 80 µs until a good signal-to-noise was obtained for both ratios of Cu(II) to peptide (Figure S30). To aid the explanation for the loss of signal, the same concentrations of Cu(II) were measured by ED-FS both in buffer solution and in the presence of 150 µM of MB1-2 peptide. The resulting traces show that in the presence of MB1-2, increasing the concentration of copper ion does not result in an increase in signal, and this loss could explain the reduction of RIDME modulation depth which could be due to the reduction of paramagnetic copper species bound to the peptide.

All PDS data was analyzed using experiment specific MATLAB plug-ins such as DEERAnalysis2020b,(22) for DEER and low spin (S = 1/2) RIDME, and OvertoneAnalysis2017(23) (OA) for high spin (S > 1/2) RIDME. DEER and RIDME are processed by removing background contributions to the time domain profile by fitting a background model to the experimental data using either DEERAnalysis or OvertoneAnalysis. Both homogeneous (stretched exponential) and polynomial background fitting are used using the lowest order polynomial that gave a good fit by eye. This is useful as the distance distribution, especially in the case of RIDME, can be strongly correlated to the background model and parameters used when performing the fit. The dimension of the homogeneous model or polynomial fit are reported in the figure captions. The corrected data is then fit and transformed into a distance distribution using Tikhonov regularization through the DEERAnalysis module where an optimum regularisation parameter is found by the L-curve criterion within the software.

The assumption behind the method is that the paramagnetic centers are coupled only through the dipolar interaction in the weak coupling case and with no orientation selection, that the centers are spin-half and that the *g*-factors are isotropic and as for a typical nitroxide. Therefore, the Gd(III) DEER data are analyzed assuming that Gd(III) can be well-approximated as a spin-half center, though some account of its high spin nature is taken into account for analysing the RIDME data, see later. Not accounting for the difference in *g*-values will lead to a small deviation for the Gd(III)-TOAC measurement; the error is a little larger for Cu(II)-TOAC since the *g*-values for Cu(II) deviate more from those of the nitroxide (the extracted distances may be of order 0.05 nm too short).(24) Although not explored here, since the effect was not obvious, there is a possibility of an orientation selection bias in the Cu(II)-TOAC DEER which would not be present in the Gd(III)-TOAC DEER (or Cu(II)-TOAC RIDME).(21) These various effects complicate the exact extraction of distances, but the distortion from the true values are likely to be small for the data we present here.

Validation of the PDS data is performed on both methods of background model fitting through DEERAnalysis. The noise contribution to the error and the background contribution are treated as free parameters when performing error validation. Briefly: the uncertainties in the noise are modelled by the introduction of pseudorandom numbers, raising the noise level by a given value (the parameter L was set to 1.5). This is combined with the uncertainties in the background correction to better estimate the error, which is modelled through variation of the start time for performing the background fitting using the DeerAnalysis predefined fine-grid. This provides a large number of possible trials for Tikhonov regularisation and provides an estimate of upper and lower bounds of certainty on distances and distributions. Graphs of the validated results (Figures S25, S26 and S27) are plotted showing the best from the validation (solid colour), as well as error ranges (shaded). Distance distribution results from the best fit and validation have the modal distance (m), average over whole distribution (av), and standard deviation over whole distribution (σ) with associated Tikhonov regularisation parameter (α), reported in the caption and are provided by DeerAnalysis. In the main paper the reported distance values are peak-picked so that only the statistics on the main peak are given and these are the mode and full-width-half-height (FWHH).

RIDME experiments using a high spin (S > 1/2) center (Figure S26) contain the desired dipolar coupling frequency but also higher frequency overtones in the time-domain data due to the increased number of spin manifolds contributing, which results in artifact peaks in the distance distribution. These can be damped, allowing for better assignment of 'real' experimental peaks using OvertoneAnalysis2017 where the data is processed and validated as described for low-spin RIDME, with two additional coefficients P_1 and P_2 allowed to fit to correct for the first and second harmonic overtones. Fitting of the Gd(III) data found $P_1 = 0.5$ and $P_2 = 0.094$ to be good parameters.

The legends for the 5p-RIDME experiments give key parameters for the experiment as [operating temperature in K - Mixing time in μs - Observation π-pulse length in ns (paramagnetic center position)].

NMR Spectroscopy: ^{1}H NMR measurements at 7 T were performed on a Bruker Avance III HD spectrometer, with a resonance frequency of 300.13 MHz. Data were acquired at 20 ± 0.2°C, using 5 mm ^{1}H coil in a Bruker Diff30 probe and 3 mm NMR tubes. A 90° pulse duration calibrated for each sample was used for the experiments. For T_1 NMR relaxation measurements, a saturation recovery sequence was used. A series of 8 spectra were collected with logarithmically spaced recovery delays from 0.01 s to a maximum of 12-15 s, 8 signal averages, 8k points with a spectral width of 10 kHz. For T_2 NMR relaxation measurements a Carr-Purcell-Meiboom-Gill (CPMG) pulse sequence was used with 15 s repetition time (> $5T_1$), 8 signal averages, 16k data points and a spectral width of 10 kHz. The variable counter list for the CPMG experiments was spaced with 10 values from 2 to 1024 with an echo time value of 2 ms.

^{1}H NMR measurements at 1.4 T were performed on a Spinsolve benchtop NMR (Magritek, Wellington, New Zealand), operating at resonance frequency of 60 MHz. Data were acquired at 21.0 ± 0.5°C using 3 mm NMR tubes and a 90° pulse width of 7 μs. For T_1 NMR relaxation measurements, an Inversion Recovery sequence was used with a repetition time > $5T_1$ (≥ 10 s). A series of 11 spectra were collected with inversion recovery delays linearly spaced from 0.001 s to a maximum of 5-10 s, with 8 signal averages and 32k points. For T_2 NMR relaxation

measurements a CPMG pulse sequence was used with a repetition time > $5T_1$ ($\geq$ 10 s). A series of 10 spectra were recorded with a 2 ms CPMG echo time and final echo time of 5 s, 8 signal averages and 32k data points.

All data were analysed using Prospa (Magritek, New Zealand). Signal intensity curves were fitted in KaleidaGraph (Synergy, USA).

Binding of Cu by MB1-2 was monitored by measuring the T_1 and T_2 ^{1}H magnetic resonance relaxation times of solutions of 40 μM $CuCl_2$ in the presence of increasing concentrations of MB1-2 trimer, in 100 mM HEPES buffer pH 7.0. The r_1 and r_2 relaxivities were calculated from the gradients of plots of the average, based on three independent repeats, ^{1}H magnetic resonance relaxation rates ($1/T_1$ and $1/T_2$ respectively), as a function of $CuCl_2$ concentration. A range of solutions were measured, containing 0, 10, 20, 30, 40 and in some cases 80 μM $CuCl_2$ and 15 equivalences of peptide monomer (5 equivalences peptide trimer), to drive Cu(II) complexation. Analogous control experiments were performed for solutions of $CuCl_2$ in the absence of MB1-2, and for MB1-2 in the absence of $CuCl_2$. The r_2 relaxivity of the apo MB1-2 peptide at 300.13 MHz was used to correct r_2 for the Cu(MB1-2)$_3$ and Gd(MB1-2)$_3$ complexes, by accounting for the contribution of excess unbound MB1-2 (MB1-2: 7 T r_1 = 0.0 ± 0.0 mM^{-1} s^{-1}; r_2 = 0.48 ± 0.01 mM^{-1} s^{-1}; 1.4 T r_1 = 0.02 ± 0.01 mM^{-1} s^{-1}; r_2 = 0.09 ± 0.01 mM^{-1} s^{-1}). Relaxivity experiments for $CuCl_2$ (0, 10, 20, 30 and 40 μM), in the presence of MB1C (15 equivalence monomer), were also performed. Relaxivity experiments were performed on solutions prepared in 100 mM HEPES buffer pH 7.0.

Relaxivity experiments for $CuCl_2$, in the absence and presence of MB1-2 (15 equivalence monomer), were also performed in nanopure water in the absence of HEPES buffer. For these aqueous solutions, the pH was manually adjusted to 7, by the addition of concentrated stock solutions of NaOH and HCl (0.01, 0.1 and 1 M). The r_1 and r_2 relaxivities were determined from plots of the average, based on three independent repeats, relaxation rate ($1/T_1$ and $1/T_2$ respectively) as a function of $CuCl_2$ concentration (0 to 40 μM).

2. Impact of glycerol on Cu(II) binding to MB1-2

To determine the effect of the glycerol on the local structure of the copper centre, control samples of a 1 mM $CuCl_2$ solution in 50 mM HEPES buffer pH 7.0, in the absence and presence of 50% glycerol, were measured by XAS. The absorption spectra of both samples are remarkably similar, indicating that the presence of glycerol does not affect the local coordination around the copper (see Figure S7). A similar test was performed for a 1 mM $CuCl_2$ solution in the presence of MB1-2. In this case, the absorption spectrum in the presence of 50% glycerol shows apparent differences to the spectrum with no glycerol, see Figure S7. In the absence of glycerol, the spectrum more closely resembles that for $CuCl_2$ recorded in the absence of the MB1-2 peptide. The absorption spectrum of the sample in the absence of glycerol can be reproduced by a linear combination fit of the absorption spectra of the $CuCl_2$ solution and the $CuCl_2$/MB1-2 solution with glycerol. The fit shows that Cu(II) binding to MB1-2 is not complete in the absence of glycerol (Figure S11).

The XAS data suggest a non-innocent role of glycerol in the binding of Cu(II) to MB1-2. A CD study of 30 μM MB1-2 peptide monomer in 10 mM HEPES buffer pH 7.0 is consistent with a poorly folded peptide, however, the analogous spectrum recorded in the presence of 50% glycerol, yields a well folded α-helical peptide with minima at 208 and 222 nm (Figure S9). The molar ellipticity at 222 nm provides an estimate of 79 ± 3% folded peptide (Figure S9).(12) This behaviour is consistent with glycerol's established role as a protein stabilizing agent,(25, 26) including reports of its stabilization of coiled coils.(27) Given that enhanced folding is accompanied by the formation of a more preorganized metal binding site, it is not surprising that enhanced Cu(II) binding is observed by XAS in the presence of glycerol. The molar ellipticity of MB1-2 in the presence of 50% glycerol and in the presence of one molar equivalent of Cu(II) per trimer, increases to 81% folded, similar to the 82% folded for the analogous solution with one molar equivalent of Gd(III) (Figure S9). A titration of $CuCl_2$ into a solution of MB1-2 in the presence of 50% glycerol is shown in Figure S10, but given the small change infolding on addition of $CuCl_2$, it was not possible to reliably fit the binding curve.

3. Figure S1:

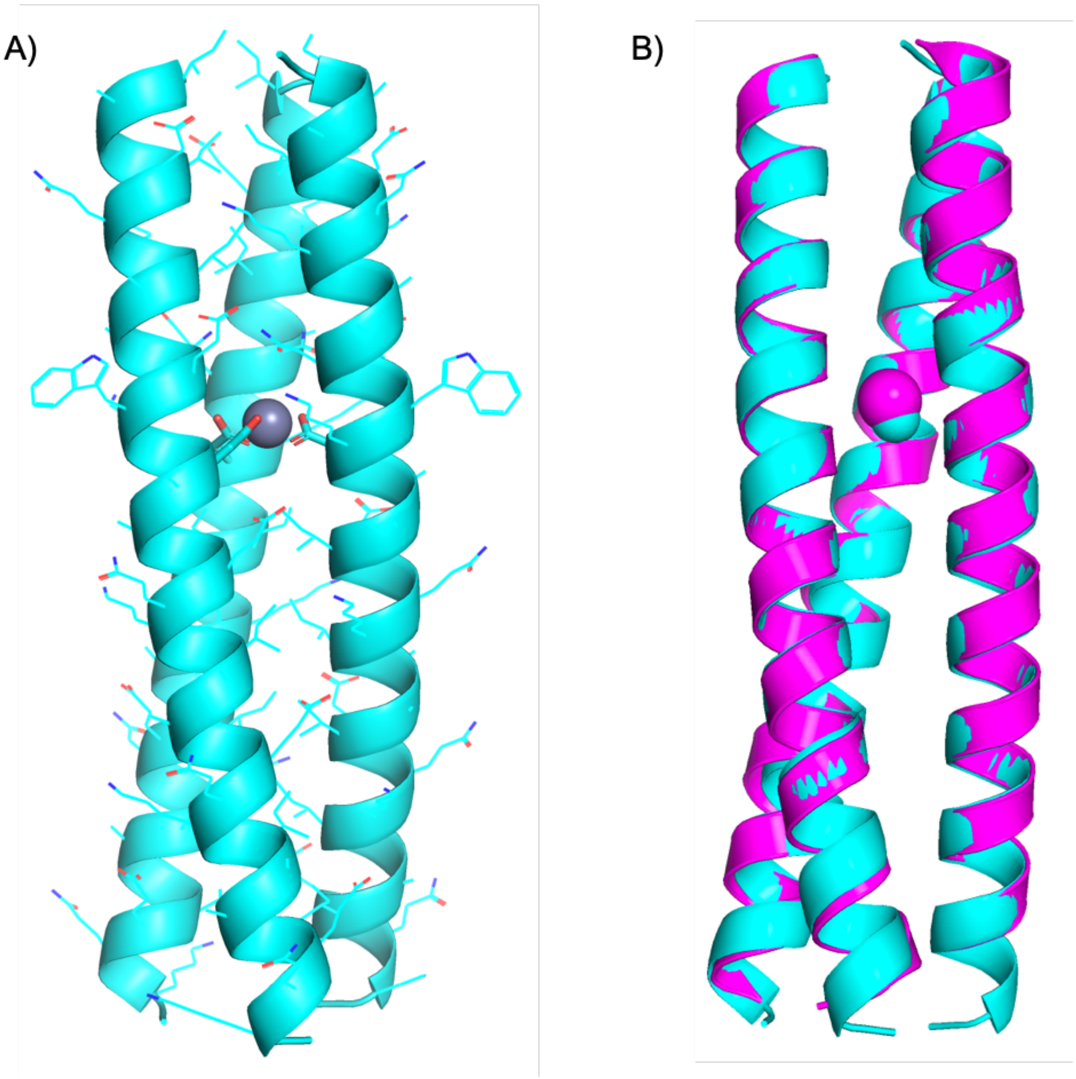

Figure S1. A) Model of MB1-2 trimer, generated using ColabFold,(9) and the transition metal binding site identified using Metal3D.(10) B) Overlay of Tb(III) bound crystal structure (magenta, based on PDB 7P3H)(28) and Cu(II) bound model (cyan). Metals are not full colocalized. Shown are main chain atoms as ribbons, amino acid side chains are shown as lines, apart from coordinating Asp residues (shown in stick form), and bound metal (sphere).

4. Figure S2:

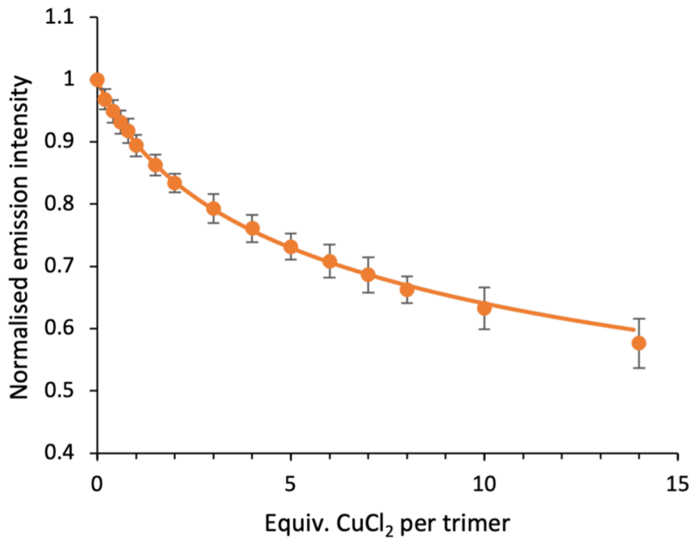

Figure S2. $CuCl_2$ (1-14 equiv. per trimer) titration into 30-32 µM MB1-2 peptide monomer in 10 mM HEPES buffer pH 7.0, monitored by fluorescence (λ_{ex} 280 nm). The plot of normalised emission intensity for the Trp, as a function of $CuCl_2$ equivalents per MB1-2 trimer, is shown fit to a nonlinear least-squares fitting based on M + 3L ↔ ML_3 binding model using DynaFit, apparent log K 4.6 ± 0.1.[43] Error bars shown for the standard deviation error of three independent repeats.

5. Figure S3:

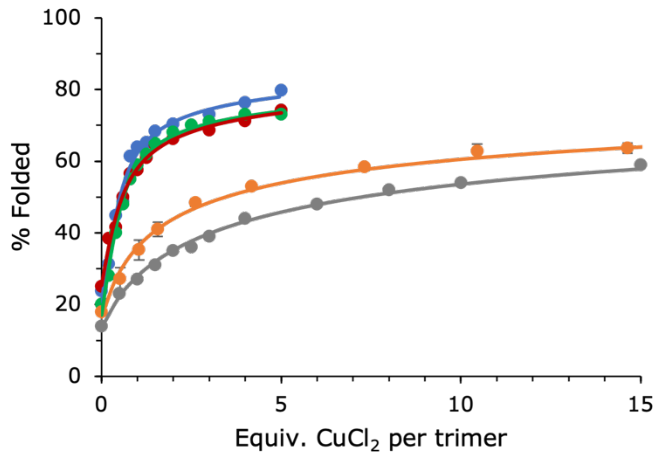

Figure S3. $CuCl_2$ titration into 17 (grey), 30 (orange), 90 (green), 120 (red) and 150 µM (blue) MB1-2 peptide monomer, in 10 mM HEPES buffer pH 7.0, monitored by CD. The plot of % folded, based on the molar ellipticity at 222 nm, as a function of $CuCl_2$ concentration, is shown fit to a nonlinear least-squares fitting based on $M + 3L \leftrightarrow ML_3$ binding model.

6. Figure S4:

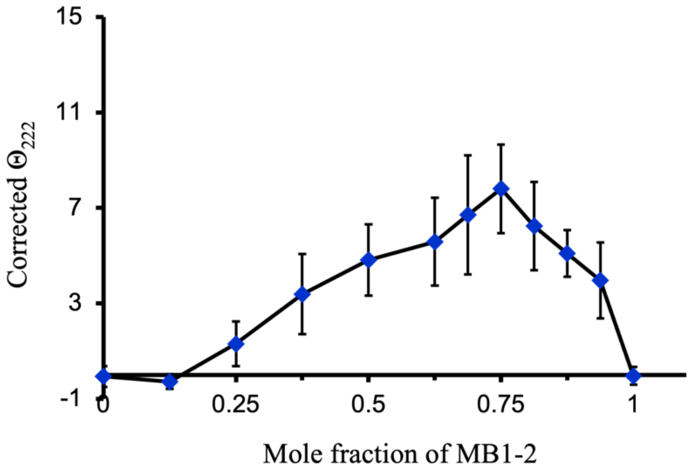

Figure S4. Job's continuous variation plot for MB1-2 and $CuCl_2$ in 10 mM HEPES buffer pH 7.0, with C_{total} constant and equal to 40 μM. Showing the mole fraction of MB1-2 monomer versus CD ellipticity at 222 nm, an indication of peptide folding, corrected for the contribution from MB1-2 in the absence of Cu(II). Error bars are the standard deviation error of three independent repeats.

7. Figure S5:

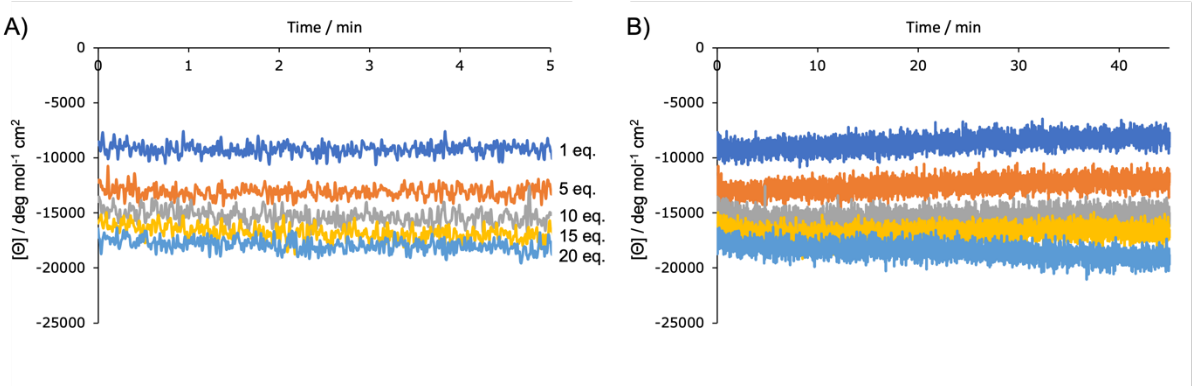

Figure S5. CD kinetic experiment of 29 µM MB1-2 monomer in the presence of 1 (dark blue), 5 (orange), 10 (grey), 15 (yellow) and 20 (blue) equivalents $CuCl_2$ in 10 mM HEPES buffer pH 7.0, monitored by the molar ellipticity at 222 nm.

8. Figure S6:

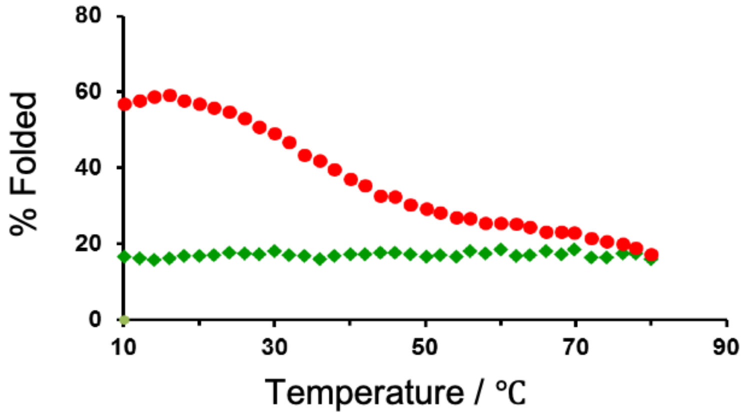

Figure S6. CD thermal unfolding of 30 µM MB1-2 monomer in the absence (green diamonds) and presence of 100 µM $CuCl_2$ (red circles) in 10 mM HEPES buffer pH 7.0, monitored by the molar ellipticity at 222 nm.

9. Figure S7:

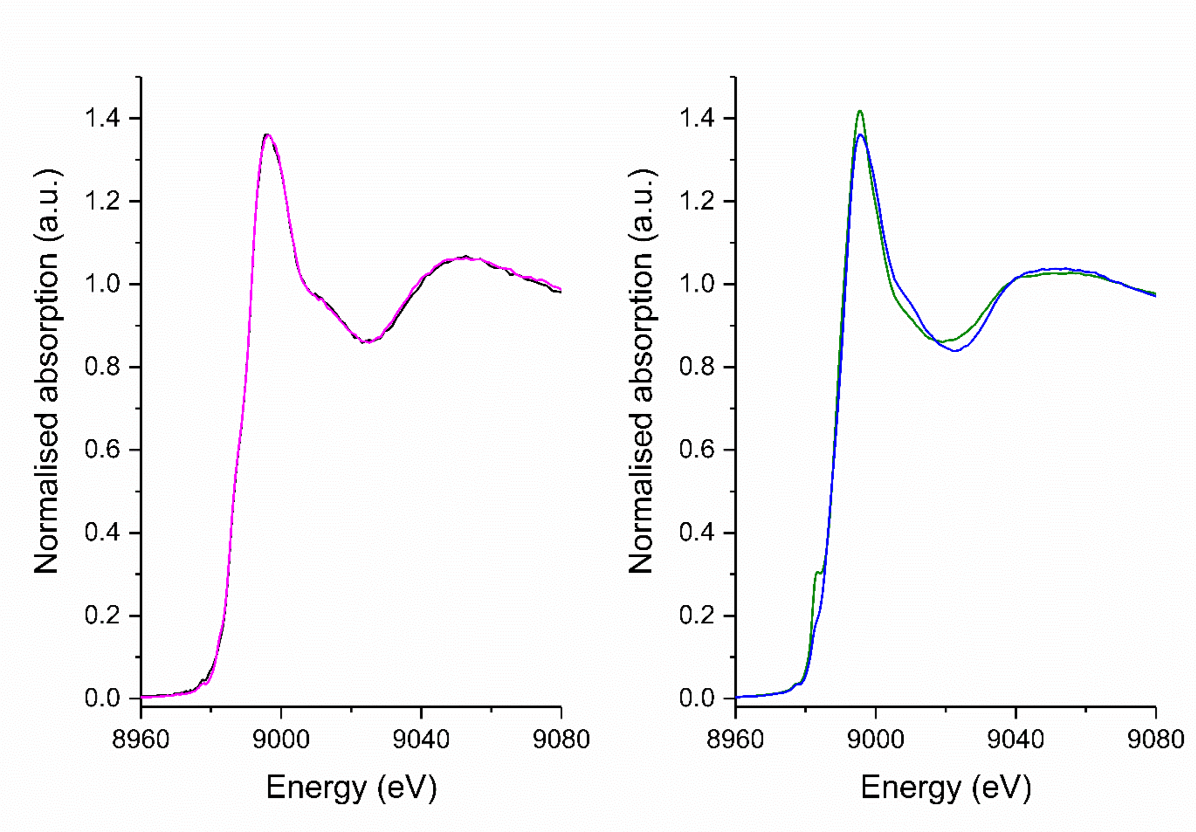

Figure S7. Cu K-edge absorption spectra (left) of a 1mM $CuCl_2$ solution in 50 mM HEPES buffer pH 7.0 in the absence (black) and presence (magenta) of 50% glycerol and (right) of a 1 mM $CuCl_2$ and 6.6 mM MB1-2 sample in 50 mM HEPES buffer pH 7.0 recorded in the absence (blue) and presence (green) of 50% glycerol.

10. Table S1. Structural parameters derived from the EXAFS analysis of $CuCl_2$ (1 mM) and MB1-2 peptide (6.6 mM peptide monomer) sample in 50 mM HEPES buffer pH 7.0, recorded in the presence of 50% glycerol, using four models.

Model		N_O	R (Å)	σ^2 (Å^2)	ΔE_0 (eV)	S_0^2	R-factor
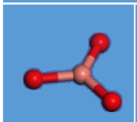	**Trigonal planar**	2.7 ± 0.6	1.95 ± 0.02	0.0032 ± 0.0024	6.6 ± 3.2	0.91	0.006
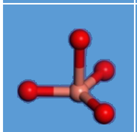	**Trigonal pyramidal**	3	1.93 ± 0.02	0.0046 ± 0.0010	2.4 ± 3.9	0.91	0.005
		1	2.35 ± 0.04				
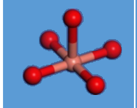	**Square pyramidal**	4	1.96 ± 0.03	0.0069 ± 0.0013	6.1 ± 5.9	0.91	0.003
		1	2.30 ± 0.08				
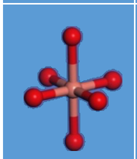	**Jahn-Teller elongated Octahedral**	4	1.94 ± 0.03	0.0074 ± 0.0013	1.5 ± 6.0	0.91	0.006
		2	2.33 ± 0.03				

N_O – oxygen coordination number, R – Cu-O distance, σ^2 – Debye-Waller factor, S_0^2 – amplitude reduction factor, DE_0 – inner potential correction, R-factor – evaluation of the percentage misfit between data and theory.

11. Figure S8:

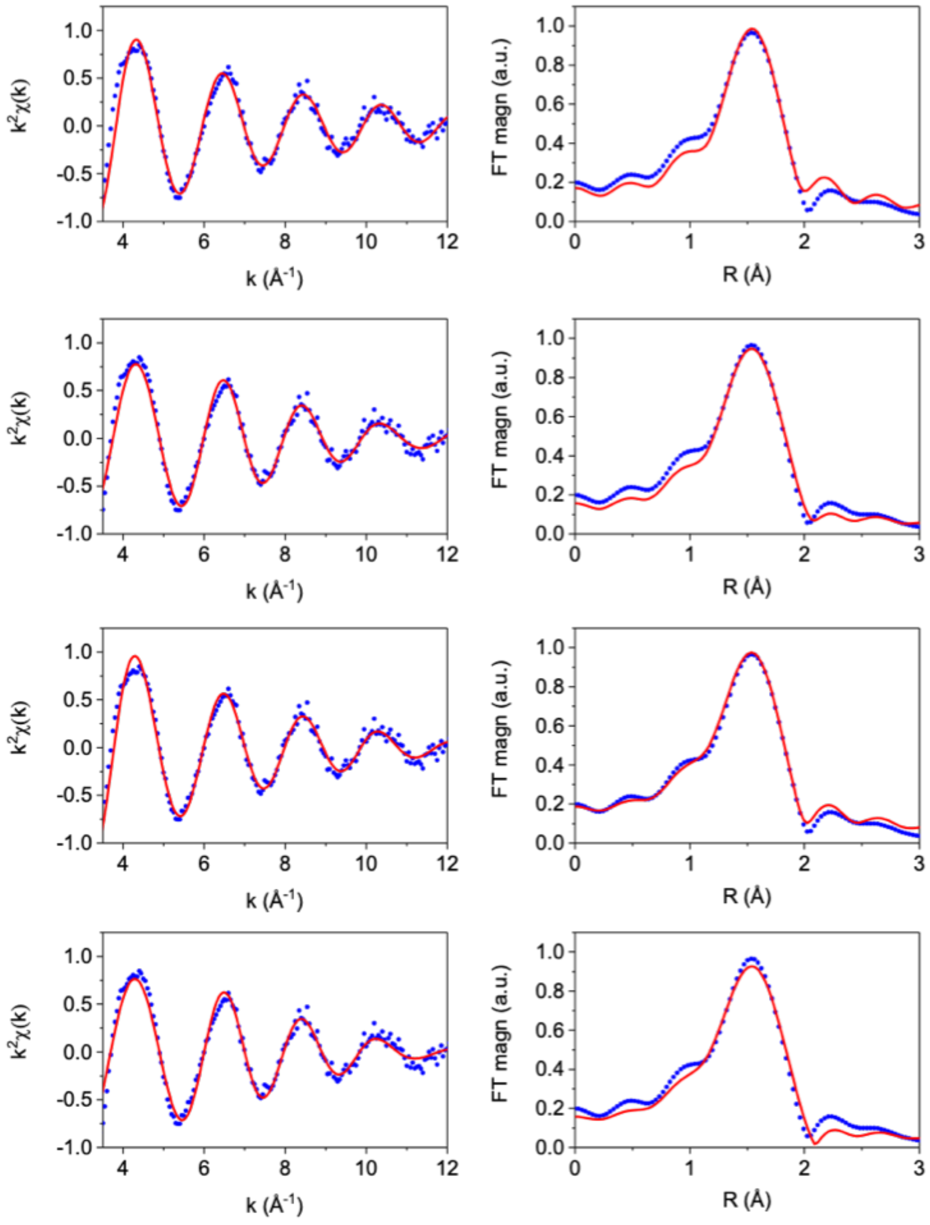

Figure S8. k^2-weighted extracted EXAFS signal (left) and corresponding Fourier transform magnitudes (right) for a $CuCl_2$ (1 mM) and MB1-2 peptide (6.6 mM monomer) sample in 50 mM HEPES buffer pH 7.0 recorded in the presence of 50% glycerol. The experimental data (blue circles) are shown with the best fit (red solid line) for the following CuO_x EXAFS models (top – bottom): trigonal planar, trigonal pyramidal, square pyramidal and Jahn-Teller distorted octahedral.

12. Figure S9:

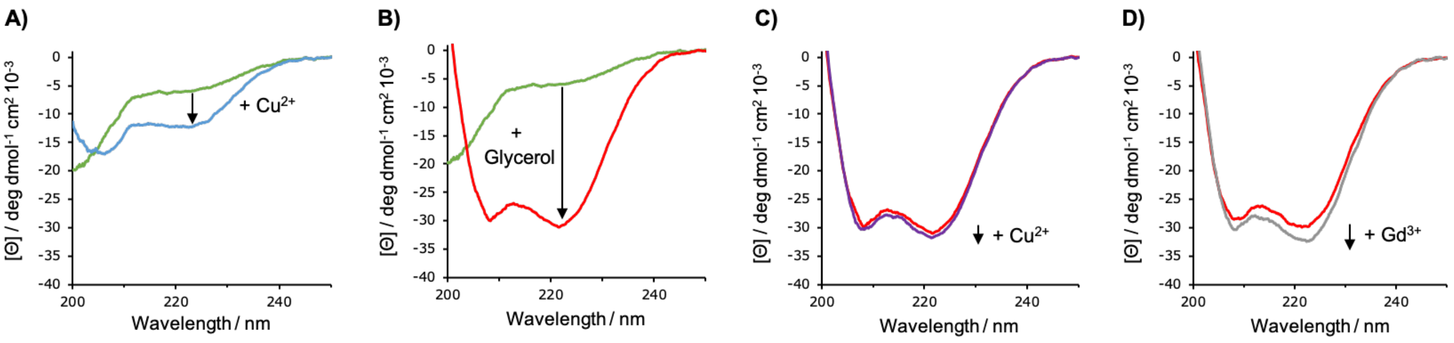

Figure S9. CD spectra of apo 30 μM MB1-2 monomer in 10 mM HEPES buffer pH 7.0, in A) the absence (green) and presence (blue) of 10 μM $CuCl_2$, B) the absence (green) and presence (red) of 50% glycerol, a cryo-protectant routinely employed in XAS and EPR, and C) and D) in the presence of 50% glycerol and of 10 μM $CuCl_2$ (purple) and $GdCl_3$ (grey), respectively.

13. Figure S10:

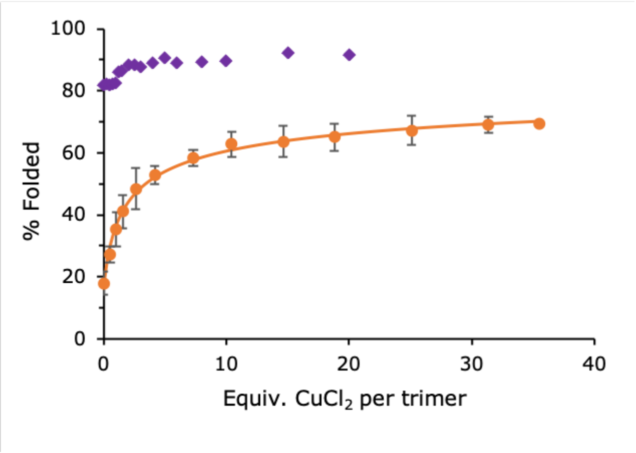

Figure S10: $CuCl_2$ titration into 30 μM MB1-2 peptide monomer in 10 mM HEPES buffer pH 7.0, monitored by CD, in the absence (orange) and presence of 50% glycerol (purple). The plot of % folded, based on the molar ellipticity at 222 nm, as a function of $CuCl_2$ concentration, is shown.

14. Figure S11:

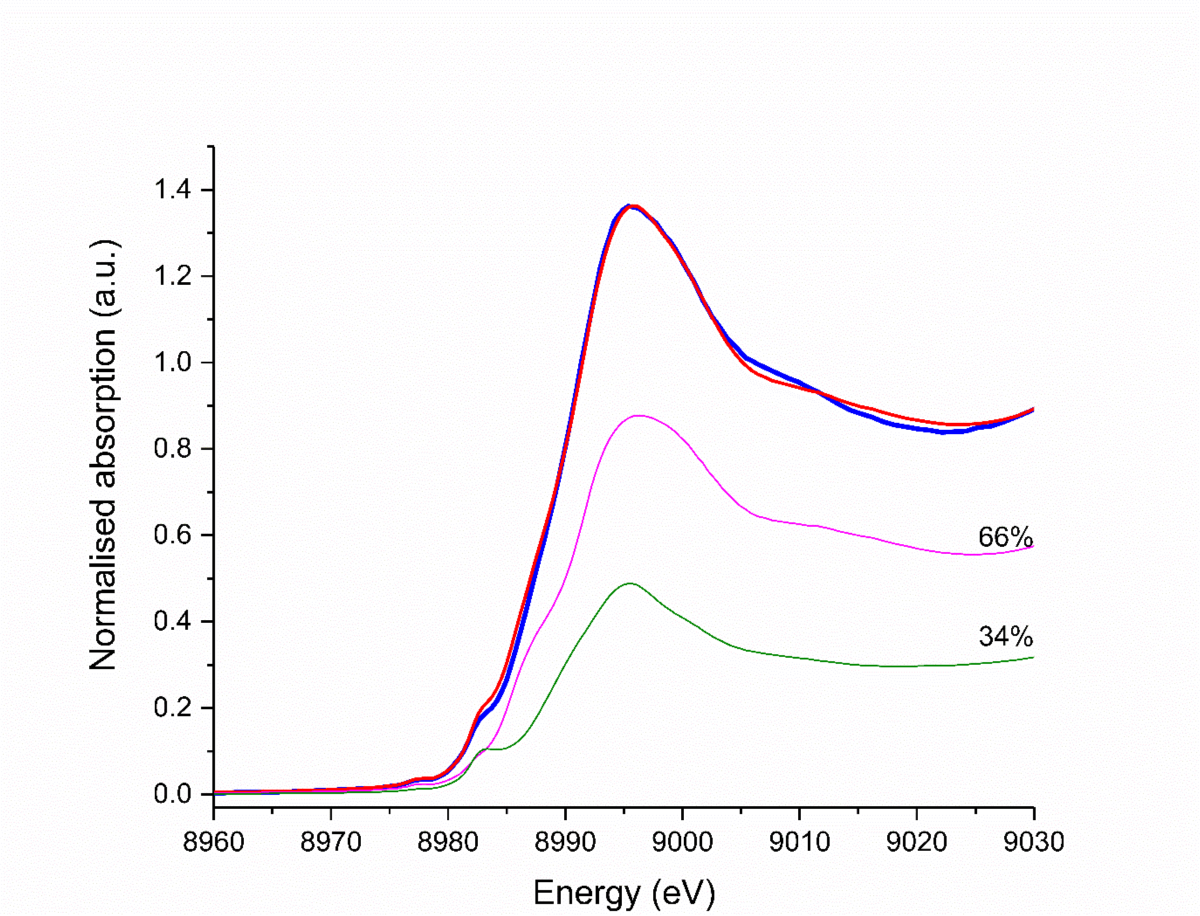

Figure S11. Cu K-edge absorption spectrum of a 1mM $CuCl_2$ and 6.6 mM MB1-2 sample in 50 mM HEPES buffer pH 7.0 recorded in the absence of glycerol (blue line). Best fit linear combination (red line) of the absorption profiles: 34% $CuCl_2$ (1 mM) and MB1-2 (6.6 mM peptide monomer) sample in 50 mM HEPES buffer pH 7.0 recorded in the presence of 50% glycerol (green line), and 66% $CuCl_2$ (1 mM) sample in 50 mM HEPES buffer pH 7.0 in the presence of 50% glycerol (magenta line). Percentages based on the assumption that data recorded in the presence of glycerol corresponds to 100% bound Cu(II).

15. Figure S12:

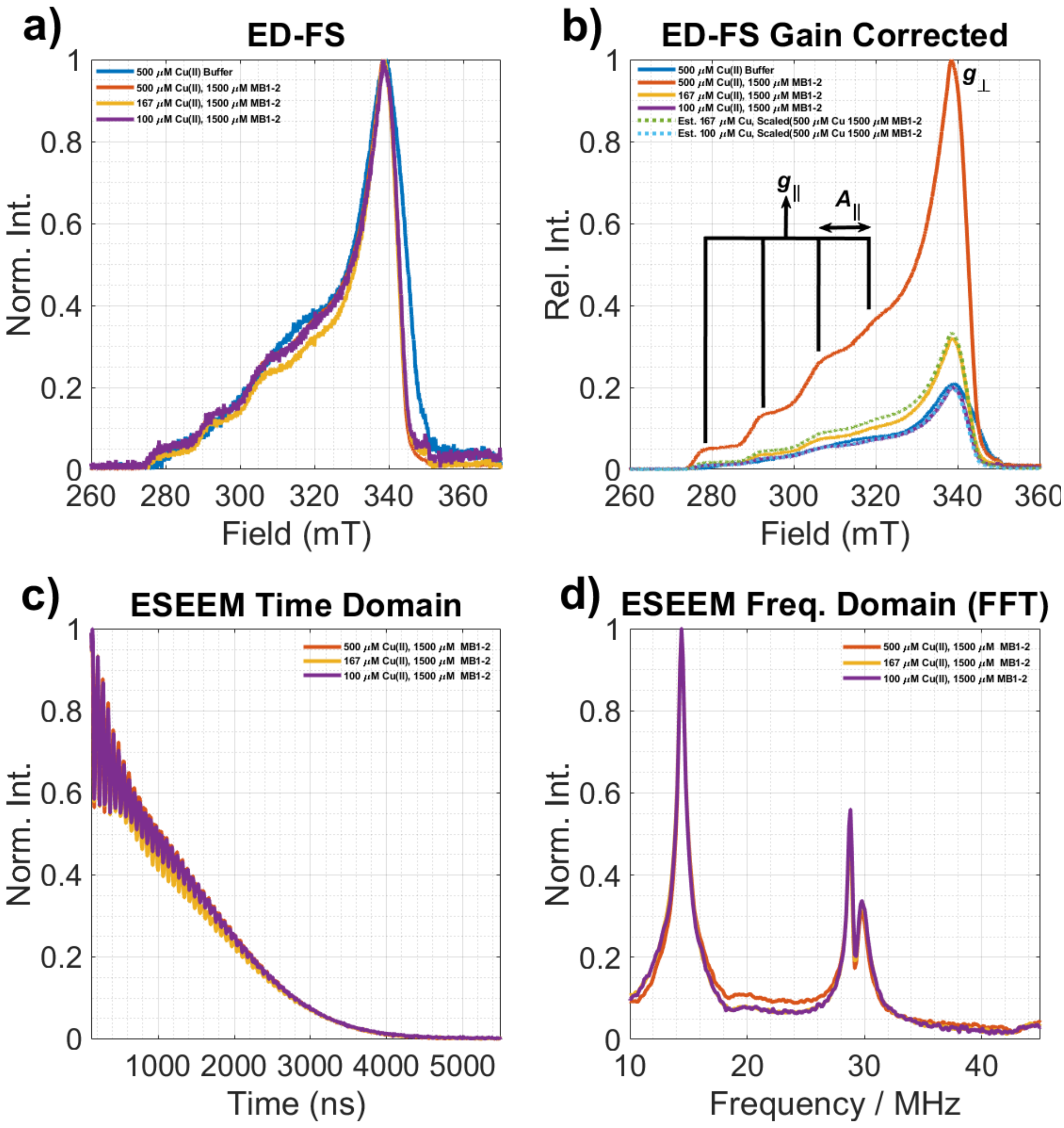

Figure S12. (a) Echo detected field-swept (ED-FS) data for Cu(II) in the presence of protonated solvent, buffer only (blue), 500 μM Cu(II) with 1500 μM MB1-2 (red), 167 μM Cu(II) with 1500 μM MB1-2 (yellow), and 100 μM Cu(II) with 1500 μM MB1-2 (purple). Measurements taken at 10 K at X-band (ν ~ 9.82 GHz) and normalized to the maximum. (b) the data presented in panel (a) scaled according to the video gain dB used to acquire the measurement in order to highlight the changes in relative intensity according to concentration

of paramagnetic metal present. The dashed lines represent the expected intensity lines for a given concentration by dividing the 500 μM Cu(II) with 1500 μM MB1-2 spectral signal by 3 (green) and 5 (aqua), respectively. The parallel and perpendicular orientations of the *g*-factor in the field spectra are annotated. The ED-FS spectra show the characteristic 'normal' shape of a tetragonal Cu complex where $g_{\perp} > g_{\parallel} > g_e$ with elongated octahedral geometry. Where the parallel orientation hyperfine splitting is particularly sensitive to the coordination environment and donor ligands.(29, 30) The sample with peptide present shows a much stronger signal than the sample in buffer without peptide. We attribute this observed 'missing signal' to some of the Cu(II) in solution interacting to form bridged copper dimers and thus quenching the paramagnetism. (c) Time domain data for 2-pulse ESEEM experiments performed on the 500 μM (orange), 167 μM (yellow) and 100 μM Cu(II) (purple) samples. (d) Frequency domain data of panel c, processed through background correction, hamming window, zero-filling and finally FFT. The relative intensities of the proton peaks present in the 2p-ESEEM remain the same with increasing ratios of MB1-2 to Cu(II). This, with the estimated scaling of the echo-detected field swept data in panel (b), leads us to conclude that the binding environments are the same across the samples, with little to no free copper being detected in the EPR measurement.

16. Figure S13:

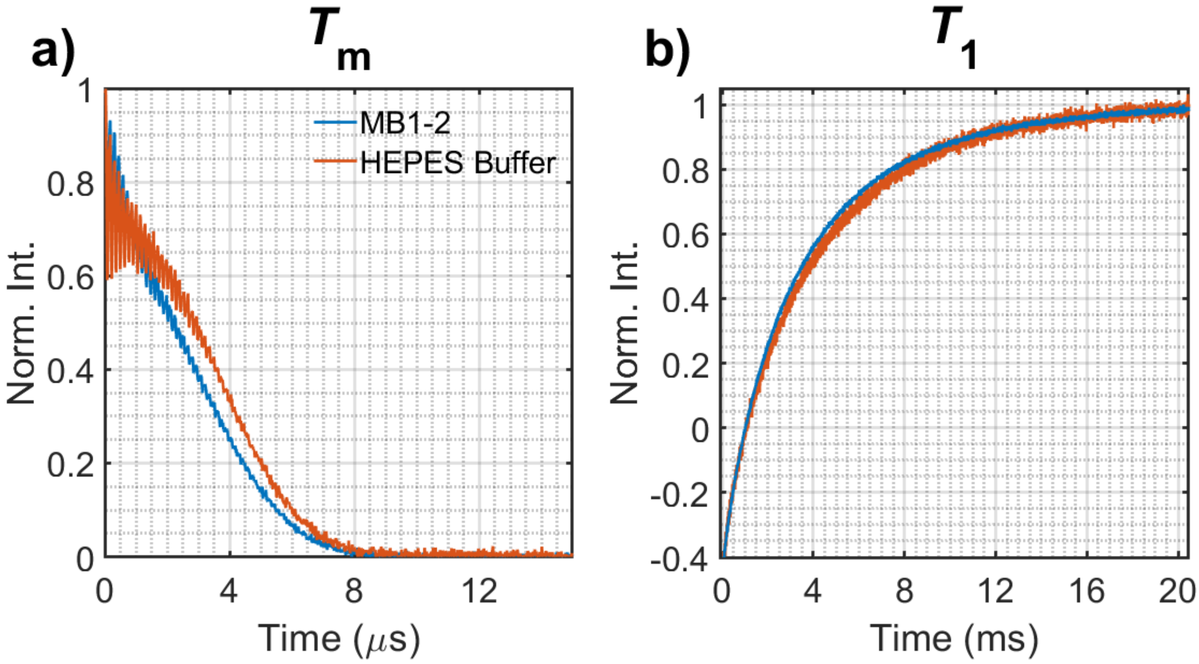

Figure S13. a) Echo decay curves with time axis 2τ and b) inversion recovery plots for 500 μM Cu(II) in protonated solvent, in the absence (red) and presence of 1500 μM MB1-2 monomer (blue). Data for the T_1 is normalized against a simulated fit which extrapolates to full recovery for more accurate normalization. Differences in relaxation times of Cu(II) in the presence and absence of MB1-2 are small but indicate that the Cu(II) is bound to the peptide. Measurements taken at 10 K at X-band (ν ~ 9.82 GHz).

17. Figure S14:

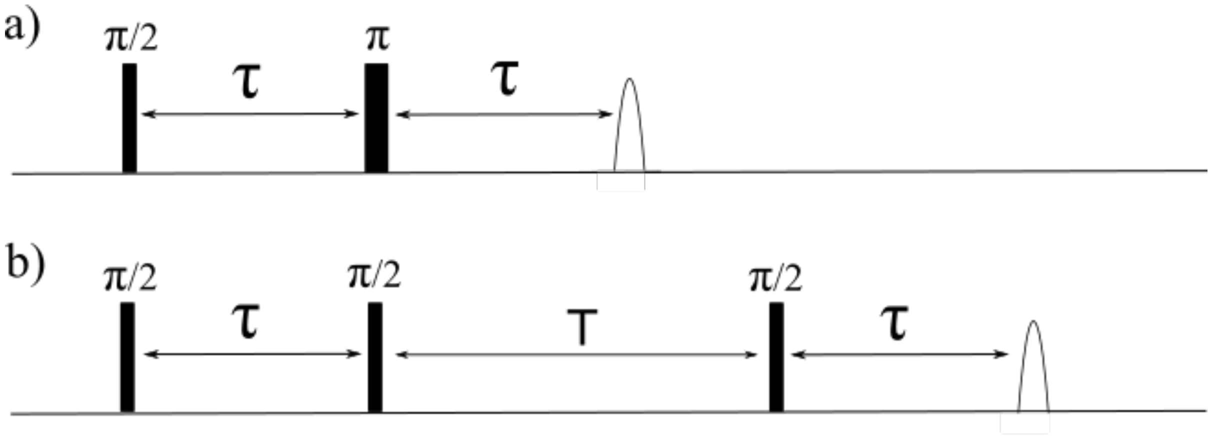

Figure S14. Pulse sequences for the a) two-pulse, and b) three-pulse ESEEM experiments. Two-pulse ESEEM measures the intensity of the primary (Hahn) echo signal as a function of the inter-pulse delay τ between the π and $\pi/2$ pulses as it is stepped incrementally. Three-pulse ESEEM measures the intensity of the stimulated echo as a function of the inter-pulse delay between the second and third pulses, T, is stepped in time.

18. Figure S15:

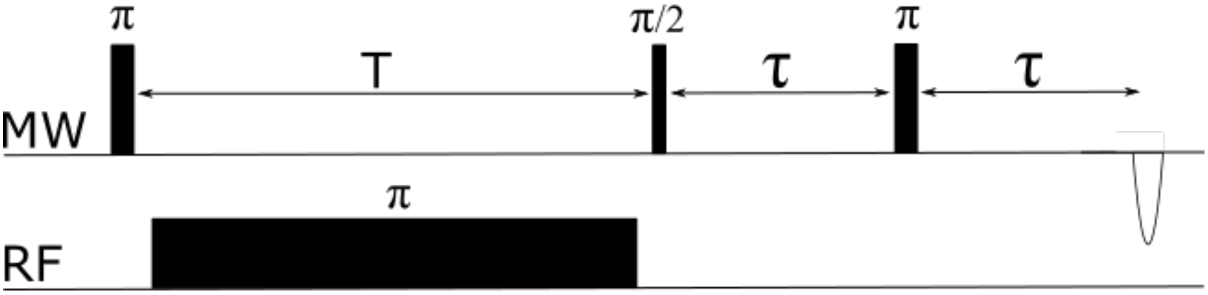

Figure S15. Microwave/radiofrequency pulse Davies ENDOR sequence. A preparation π pulse inverts the spin magnetization after which a π radio-frequency pulse is applied during the mixing period T. If the RF pulse is on resonance with any NMR transitions, there will be magnetization transfer between the spin manifolds. This is detected using a π- π/2 Hahn echo sequence.

19. Figure S16:

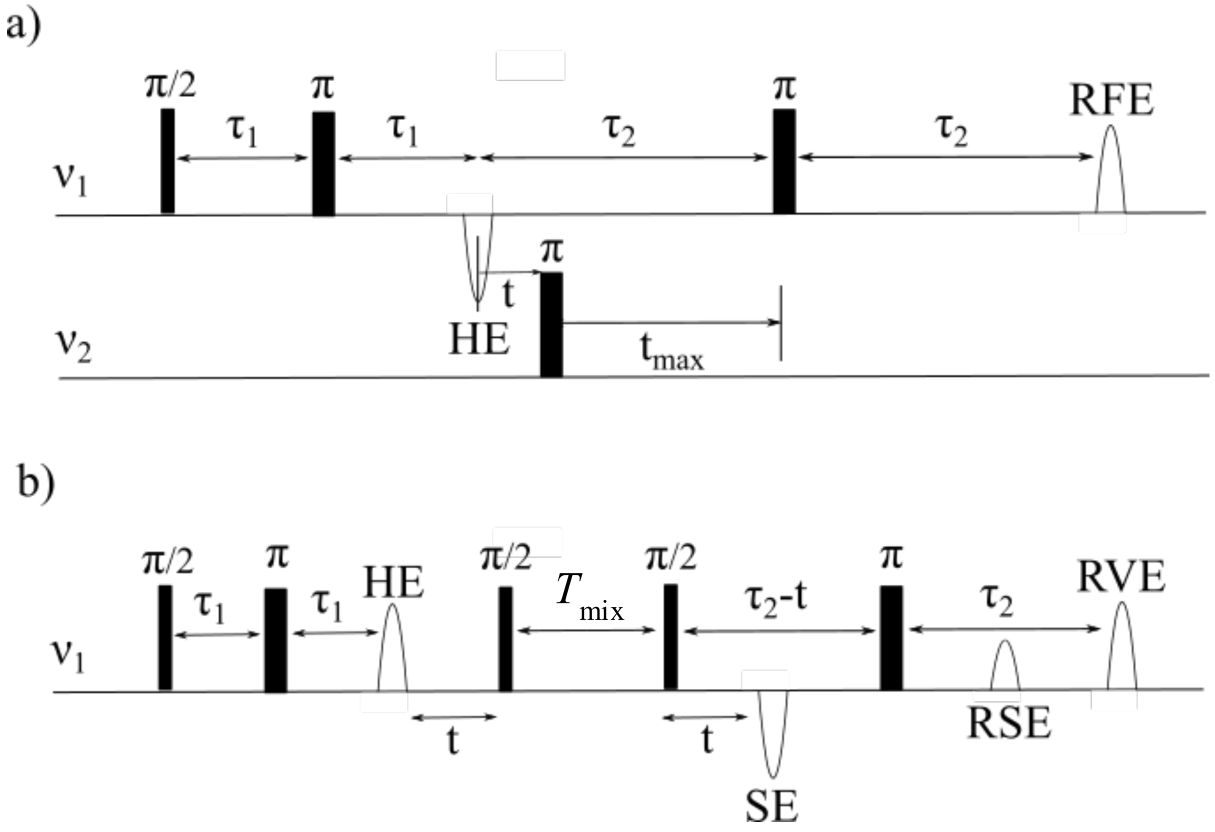

Figure S16. The four-pulse DEER (a) and the five-pulse RIDME (b) experiments. The positions of the primary Hahn echo (HE), virtual (VE), refocused (RFE), stimulated (SE), refocused stimulated (RSE) and refocused virtual (RVE) echoes are marked in the pulse sequences. Four-pulse DEER is a two frequency technique where a primary echo is formed by a Hahn echo sequence to prepare the observed spins at frequency 1 (ν_1) and then a π pulse at frequency 2 (ν_2) is applied during the free evolution period $\tau_1 + \tau_2$ and stepped in time, which acts to pump the second group of spins. This modulates the primary echo by the dipolar coupling frequency, which is detected by a refocused echo which is obtained by a final π pulse at the observing frequency at the end of the free evolution period. In five-pulse RIDME, the pump pulse is replaced by two π/2 pulses at the observer frequency separated by a mixing time T_{mix}, making it a single frequency experiment. These pulses act to transfer electron coherence to the electron population so that it can be stored during the long mixing block (> 10 μs) so that dipolar modulation can be detected. Here, the effect of pumping on the observed spins is achieved by spontaneous relaxation of the coupled paramagnetic center occurring during the mixing time. For systems where there is a differential relaxation rate the sequence will observe

the slower relaxing spins and the mixing block time will be of order of the T_1 relaxation of the faster relaxing spins (T = T_1). By stepping this block in time, the RVE is modulated by the dipolar coupling frequency, as in the four-pulse DEER experiment.

20. Figure S17:

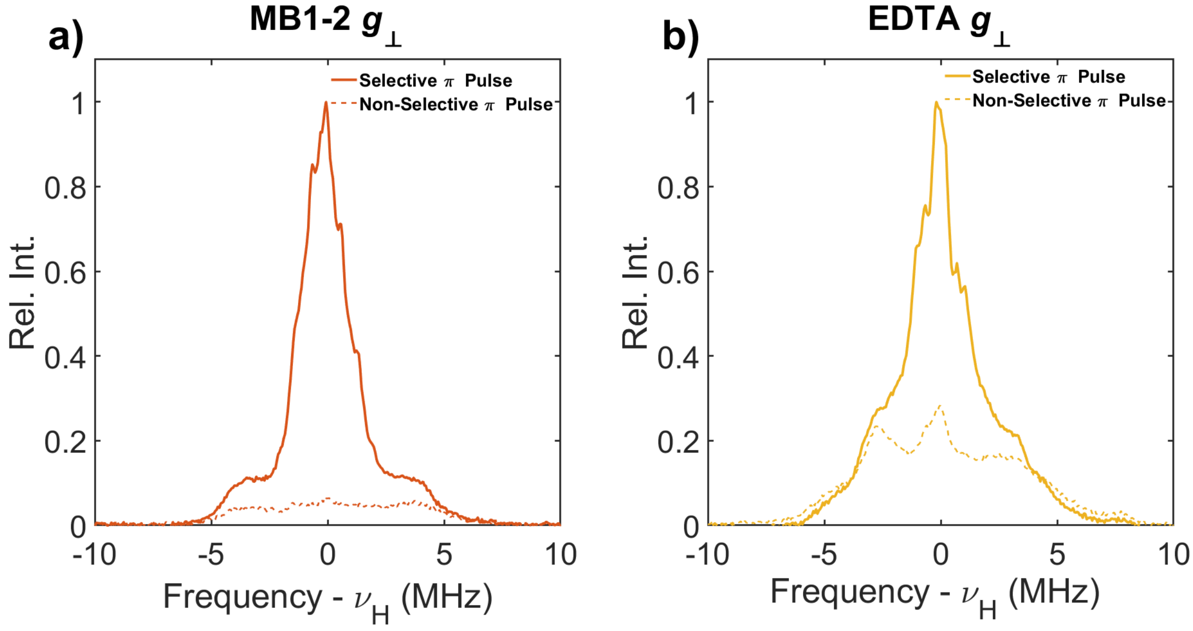

Figure S17. Pulsed ENDOR data recorded along (a) the perpendicular $g_{\perp}$ (338 mT) of the Cu(II) bound MB1-2 and (b) Cu(II) bound EDTA of the Cu(II) ED-FS (see Figure S11 for the field position). ENDOR was measured with selective (solid lines) and non-selective (dashed lines) π pulses. Panel (a): is also shown in the main paper in Figure 4b and was taken with 3 equivalents of MB1-2 (500 µM Cu(II)), but here the non-selective data is normalized against the selective-pulse data and then scaled by 8x so the differences in the spectra are more clear. Panel (b): the spectrum recorded with non-selective pulse shows the typical hyperfine and quadrupole splitting assigned to strongly coupled nitrogen where the peaks are expected to be centered at half the hyperfine coupling constant (A/2). With selective pulses the spectrum is centered around the proton Larmor frequency and is dominated by the contribution of the nearby protons. Measurements were performed with 500 µM Cu(II) in the presence of 5 equivalents EDTA. The non-selective data is normalized against the selective-pulse data and then scaled by 2.8x: additional signals due to the presence of nitrogen are clear.

21. Figure S18:

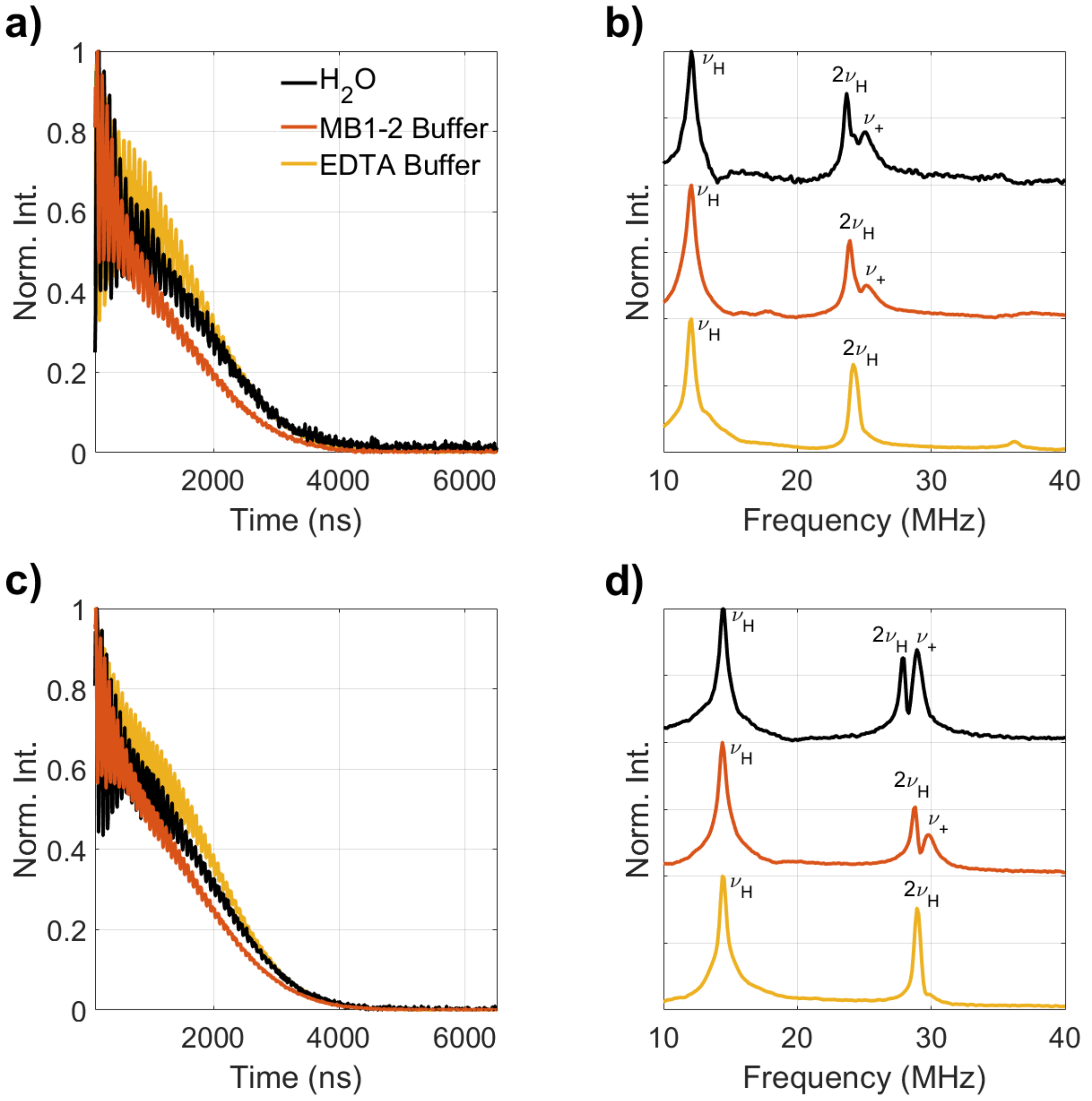

Figure S18. Frequency and time domain spectra showing the two-pulse ESEEM plots for Cu(II) in water (blue), in buffer with MB1-2 (red), and in buffer with EDTA (yellow) for the parallel (298 mT) (a,b) and perpendicular (338 mT) (c,d) orientations with respect to the Cu(II) ED-FS (Figure S11). Panels a & c show the unprocessed time-domain data, and panels b & d show the processed data in the frequency domain. A discussion of the analysis of these data is made in the Materials and Methods Section above.

22. Figure S19.i:

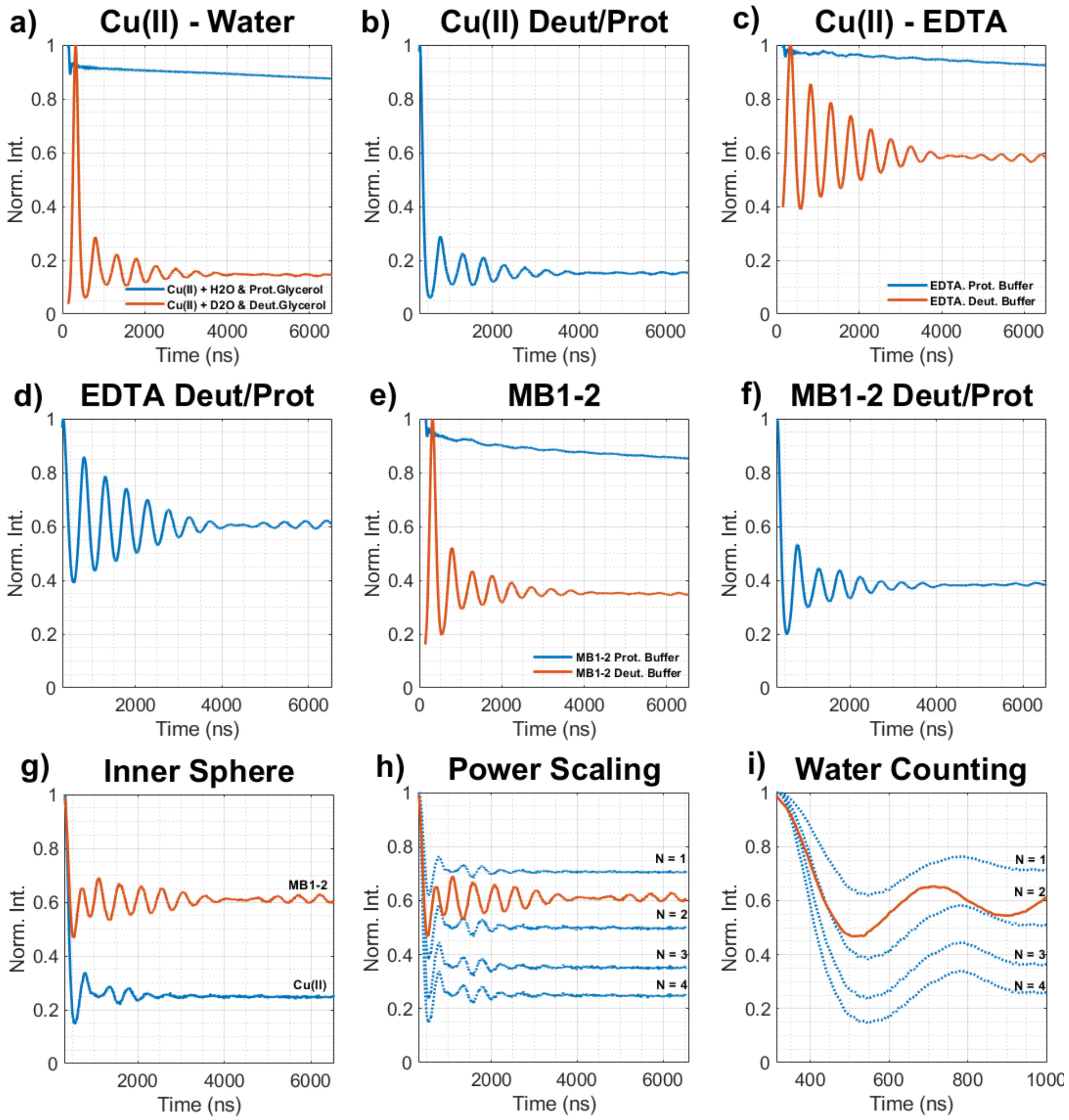

Figure S19.i: Three-pulse ESEEM time domain data for (a) Cu(II) in H_2O and D_2O, (c) Cu(II) and EDTA in H_2O and D_2O, and (e) Cu(II)+MB1-2 in protonated and deuterated solvent at the ~ $g_{\perp}$ position (in-plane) of the ED-FS. Following similar work done by Hoogstraten and Britt(18) these separate contributions are processed to remove background proton signals from the time domain data (b,d,f) and then processed to give the modulation depth for the inner sphere deuterium signal in MB1-2 (g). Power scaling the fully deuterium oxide coordinated

copper (h) allows for direct comparison of the MB1-2 inner sphere and therefore deuterium oxide (and hence water) population assignment. The first 1 μs is highlighted to show more clearly the assignment for the coordinated water in figure (i). The data is normalized against the initial intense ^{2}H modulation, and the depth modulation st closely matches the signal expected for 2 coordinated deuterium oxide (and therefore water) molecules. A further discussion of the analysis of these data is made in the Materials and Methods Section above.

22. **Figure S19.ii:**

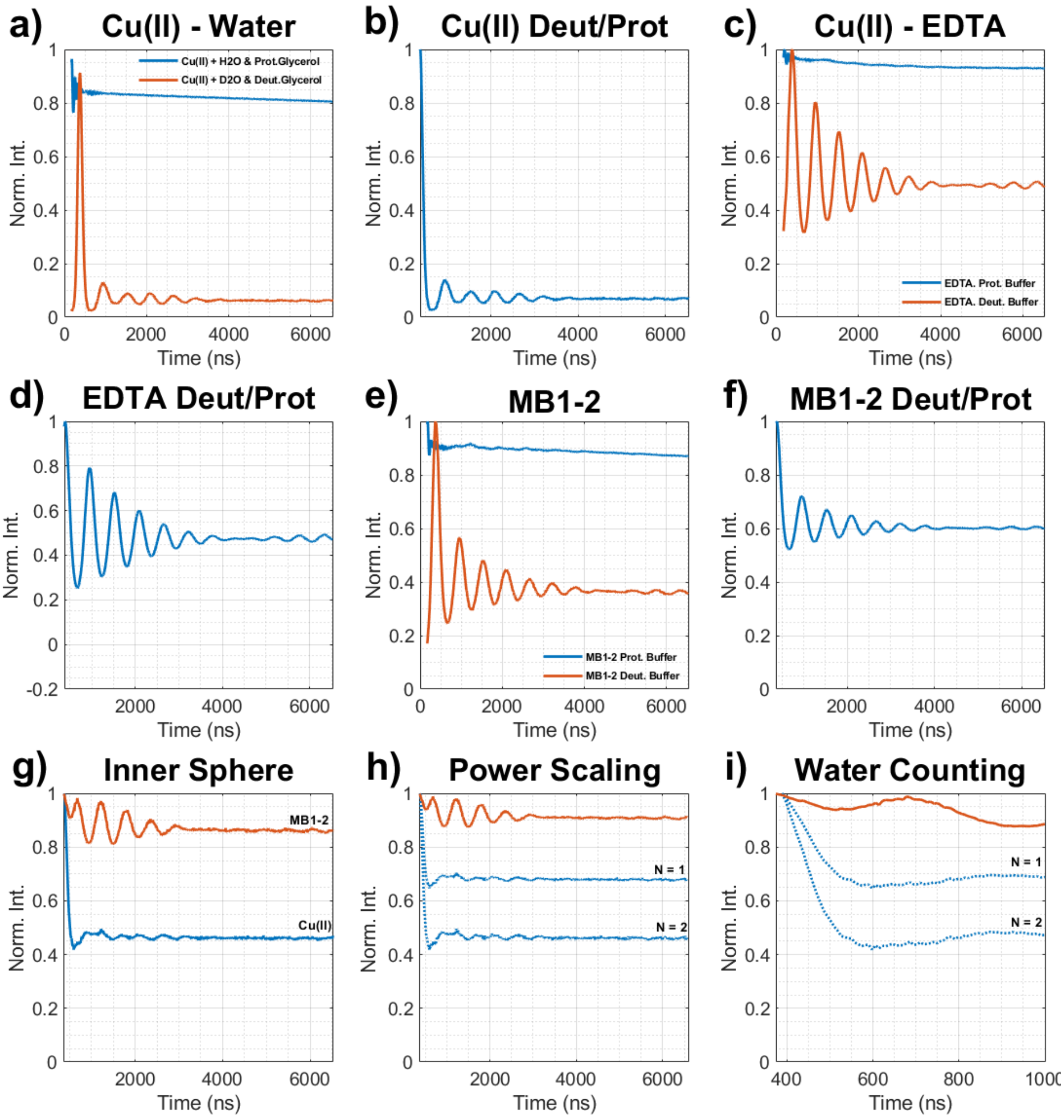

Figure S19.ii: Three-pulse ESEEM time domain data for (a) Cu(II) in H_2O and D_2O, (c) Cu(II) and EDTA in H_2O and D_2O, and (e) Cu(II)+MB1-2 in protonated and deuterated solvent at the ~ $g_{||}$ position (out-of-plane) of the ED-FS. As in the Figure S18.i, these separate contributions are processed to remove background proton signals from the time domain data (b,d,f) and then processed to give the modulation depth for the inner sphere deuterium signal in MB1-2 (g). Power scaling the fully deuterium oxide coordinated copper (h) allows for direct comparison

of the MB1-2 inner sphere and therefore deuterium oxide (and hence water) population assignment. The first 1 μs is highlighted to show more clearly the assignment for the coordinated water (i). The data is normalised against the initial intense ^{2}H modulation, and as can be see the depth does not closely match the signals expected for coordinated deuterium oxide molecules. A further discussion of the analysis of these data is made in the Materials and Methods Section above.

23. Figure S20:

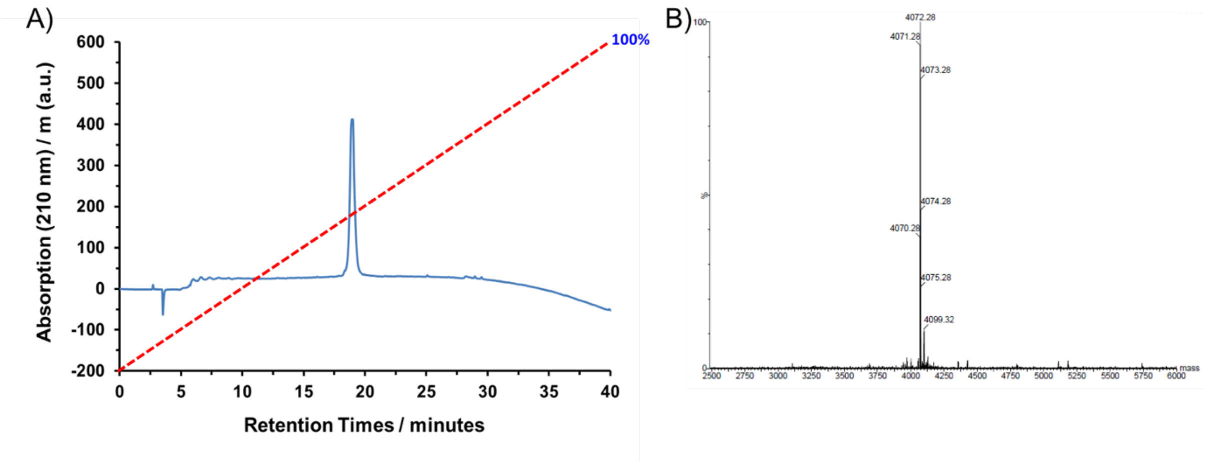

Figure S20. A) Analytical reverse phase C18-HPLC chromatograph of purified MB1-2-TOAC using H_2O/MeCN gradient (0 – 100% MeCN over 40 minutes) in the presence of 0.1% TFA. B) Deconvoluted electrospray mass spectrum of purified MB1-2-TOAC.

24. Figure S21:

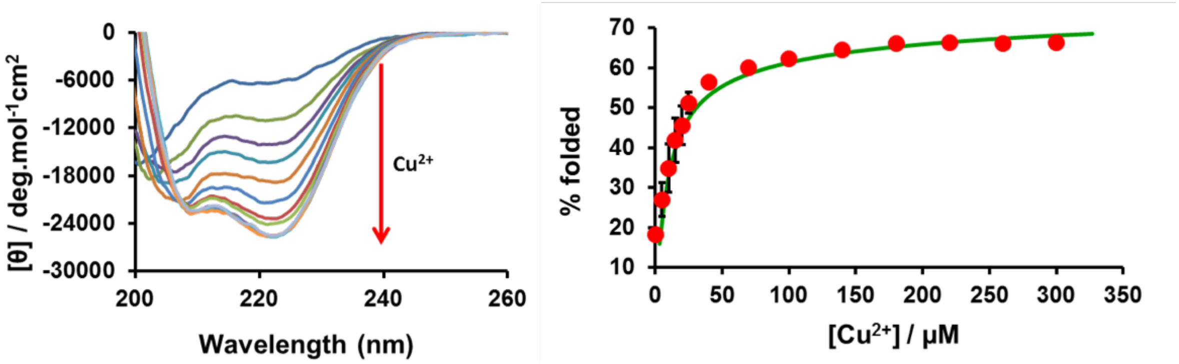

Figure S21. $CuCl_2$ (0-300 μM) titration into 30 μM MB1-2_{TOAC} peptide monomer in 10 mM HEPES buffer pH 7.0, monitored by CD. The plot of % folded, based on the molar ellipticity at 222 nm, as a function of $CuCl_2$ concentration, is shown fit to a nonlinear least-squares fitting based on M + 3L ↔ ML_3 binding model, apparent log K 4.6 ± 0.1. Error bars determined from standard deviation error of three repeat experiments.

25. Figure S22:

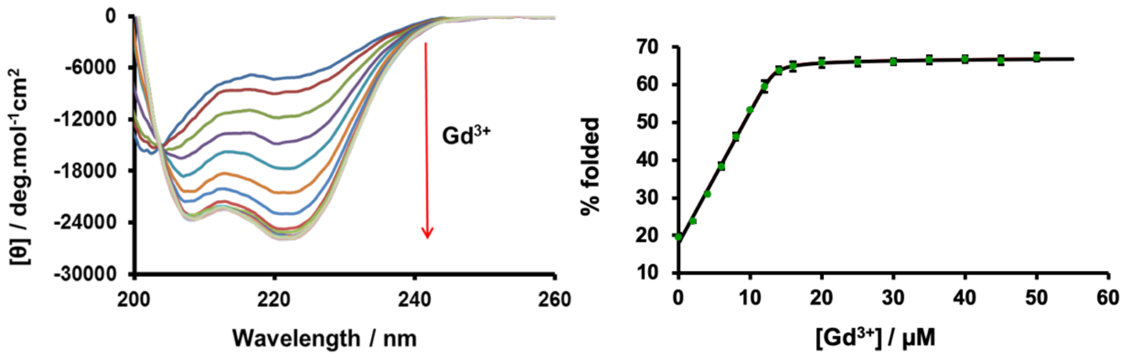

Figure S22. $GdCl_3$ (0-50 μM) titration into 30 μM MB1-2_{TOAC} peptide monomer in 10 mM HEPES buffer pH 7.0, monitored by CD. The plot of % folded, based on the molar ellipticity at 222 nm, as a function of $GdCl_3$ concentration, is shown fit to a nonlinear least-squares fitting based on $M + 3L \leftrightarrow ML_3$ binding model, apparent log K 5.8 ± 0.1. Error bars determined from standard deviation error of three repeat experiments.

26. Figure S23:

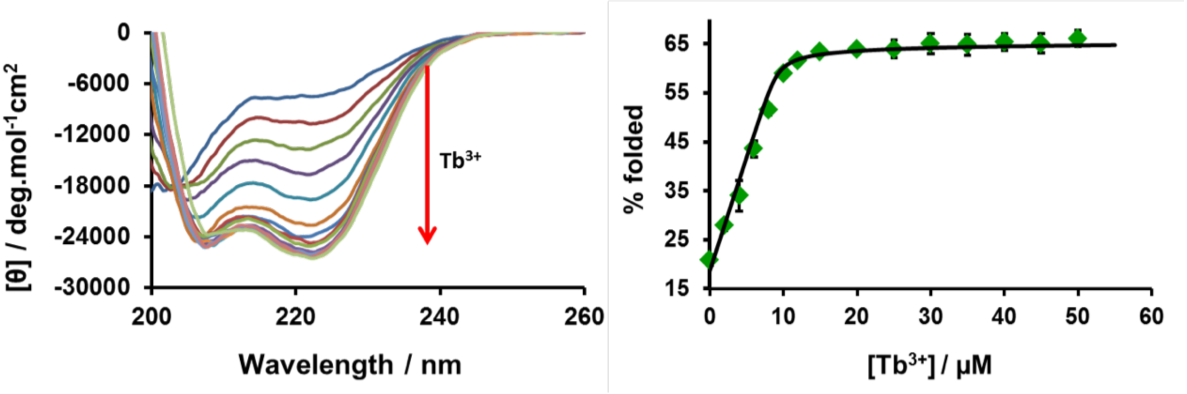

Figure S23. $TbCl_3$ (0-50 μM) titration into 30 μM MB1-2_{TOAC} peptide monomer in 10 mM HEPES buffer pH 7.0, monitored by CD. The plot of % folded, based on the molar ellipticity at 222 nm, as a function of $TbCl_3$ concentration, is shown fit to a nonlinear least-squares fitting based on $M + 3L \leftrightarrow ML_3$ binding model, apparent log K 5.7 ± 0.2. Error bars determined from standard deviation error of three repeat experiments.

27. Figure S24:

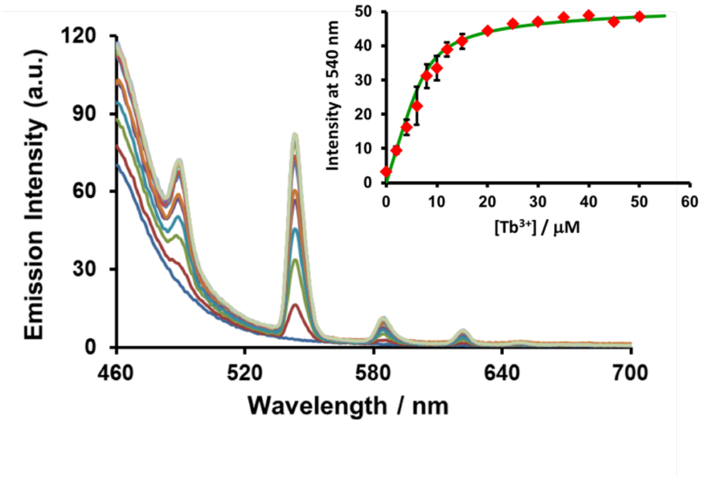

Figure S24. Emission spectra upon titration of $TbCl_3$ (0-50 μM) into 30 μM MB1-2_{TOAC} peptide monomer in 10 mM HEPES buffer pH 7.0 at 293 K, λ_{ex} = 280 nm. Inset is a plot of emission intensity at 540 nm as a function of $TbCl_3$ concentration. Data fit to M + L ↔ ML_3 model using DynaFit, apparent log *K* 5.1 ± 0.1.(31) Error bars determined from standard deviation error of three repeat experiments.

28. Figure S25:

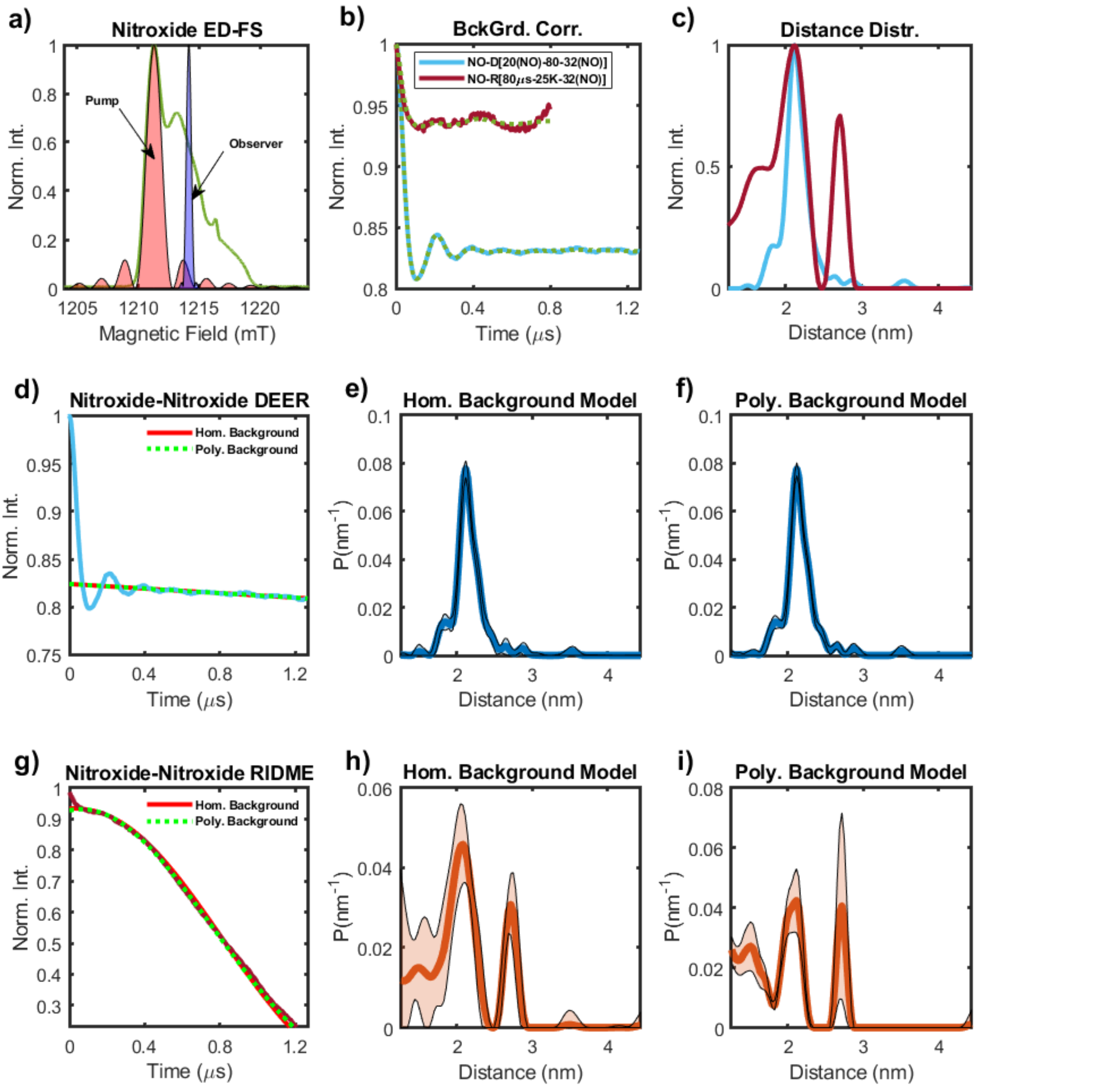

Figure S25: PDS data for the TOAC-TOAC (nitroxide-nitroxide) distances in MB1-2_{TOAC}. (a) ED-FS profile of the TOAC (green), with the excitation profiles of the observer (blue) and pump (red) superimposed over the spectral pulse positions. (b) Four-pulse DEER (D) and RIDME (R) experimental data after background correction showing homogeneous model background fitting and (c) the corresponding distance distributions. Both the DEER and RIDME experiments were performed with no metal present, all folding was assisted by the

addition of glycerol (Figure S10). The RIDME data are presented as a solution to the anomalous short distances to the distance distributions for the F-L RIDME signals when Cu(II) or Gd(III) are present (Figures S25 and S26). The RIDME data are fit to the cut data (b, cut to 800 ns) as this helped to remove the visible artifact deviations present in the fit past this time stamp while still allowing the fitting of the background in DEERAnalysis, but the background fit is shown on the full-length time-trace data. DeerAnalysis validation is provided in the panels e, f, h, i. (e) DEER, with Dim. 3, m = 2.12 nm, Avg. = 2.17, σ = 0.28 nm, α = 2.5119 (f) and 2nd-order polynomial fitted background, m = 2.12 nm, Avg. = 2.17 nm, σ = 0.27 nm, α = 2.5119. (h) RIDME, with Dim. 6.80, m = 2.10 nm, Avg. = 2.03 nm, σ = 0.42 nm, α = 12.5893 (i) and 3rd-order polynomial fitted background, m = 2.11 nm, Avg. = 2.21 nm, σ = 0.59 nm, α = 5.0119.

29. Figure S26:

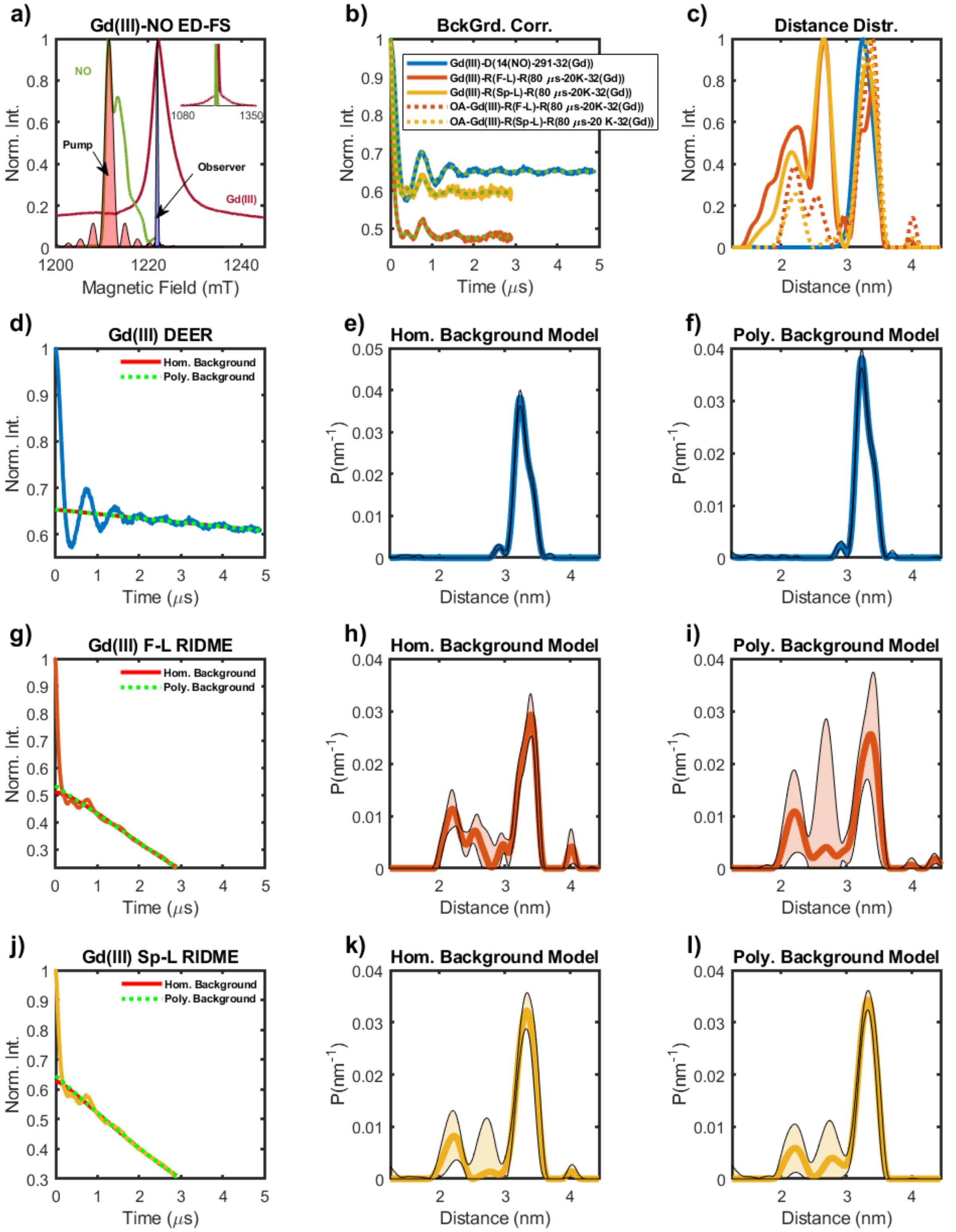

Figure S26: Gd(III) (MB1-2$_{TOAC}$)$_3$ PDS data. (a) ED-FS profile of the TOAC (green) and Gd(III) (maroon), with the excitation profiles of the observer (blue) and pump (red) superimposed over the spectral pulse positions. The pulse profiles are shown on a limited

spectral view, with the full ED-FS shown in the insert. (b) The PDS data after homogeneous background correction for four-pulse DEER (D) of Gd(III)(MB1-2_{TOAC})$_3$ (blue), five-pulse RIDME (R) for fully-labelled (F-L) Gd(III)(MB1-2_{TOAC})$_3$ (orange), and Gd(III)(MB1-2_{TOAC})$_3$ (Sp-L) RIDME (yellow). The F-L (dashed-orange) and Sp-L (dashed-yellow) data is also presented after processing using OvertoneAnalysis2017 (OA) to correct the high frequency overtones present in the Gd(III) RIDME data. (c) The distance distributions corresponding to the time traces in (b). (d, g, j) show the raw data for (b) with background corrections. (e, f, h, i, k, l) distance distributions after homogenous or polynomial background correction and their validations. Validation of RIDME data is performed on the OvertoneAnalysis corrected data using the P parameters defined from the best fit. (g) Gd(III)-nitroxide DEER with (e) Dim. 3, m = 3.23 nm, Avg. = 3.28 nm, σ = 0.21 nm, α = 63.0957and (f) 2nd-order polynomial, m = 3.23 nm, Avg. = 3.29 nm, σ = 0.22 nm, α = 63.0957. (d) Gd(III)-nitroxide (F-L) OA RIDME, with (f) Dim. 4.38, m = 3.40 nm, Avg. = 3.00 nm, σ = 0.52 nm α = 14.0248 and (g) 2nd-order polynomial, m = 3.36 nm, Avg. = 2.98 nm, σ = 0.5 nm, α = 9.0566. (j) Gd(III)-nitroxide (Sp-L) OA RIDME, with (k) Dim. 3.712, m = 3.33 nm, Avg. = 3.12 nm, σ = 0.46 nm, α = 61.2041 and (l) 2nd-order polynomial, m = 3.33 nm, Avg. = 3.11 nm, σ = 0.43 nm, α = 51.3418.

30. Figure S27:

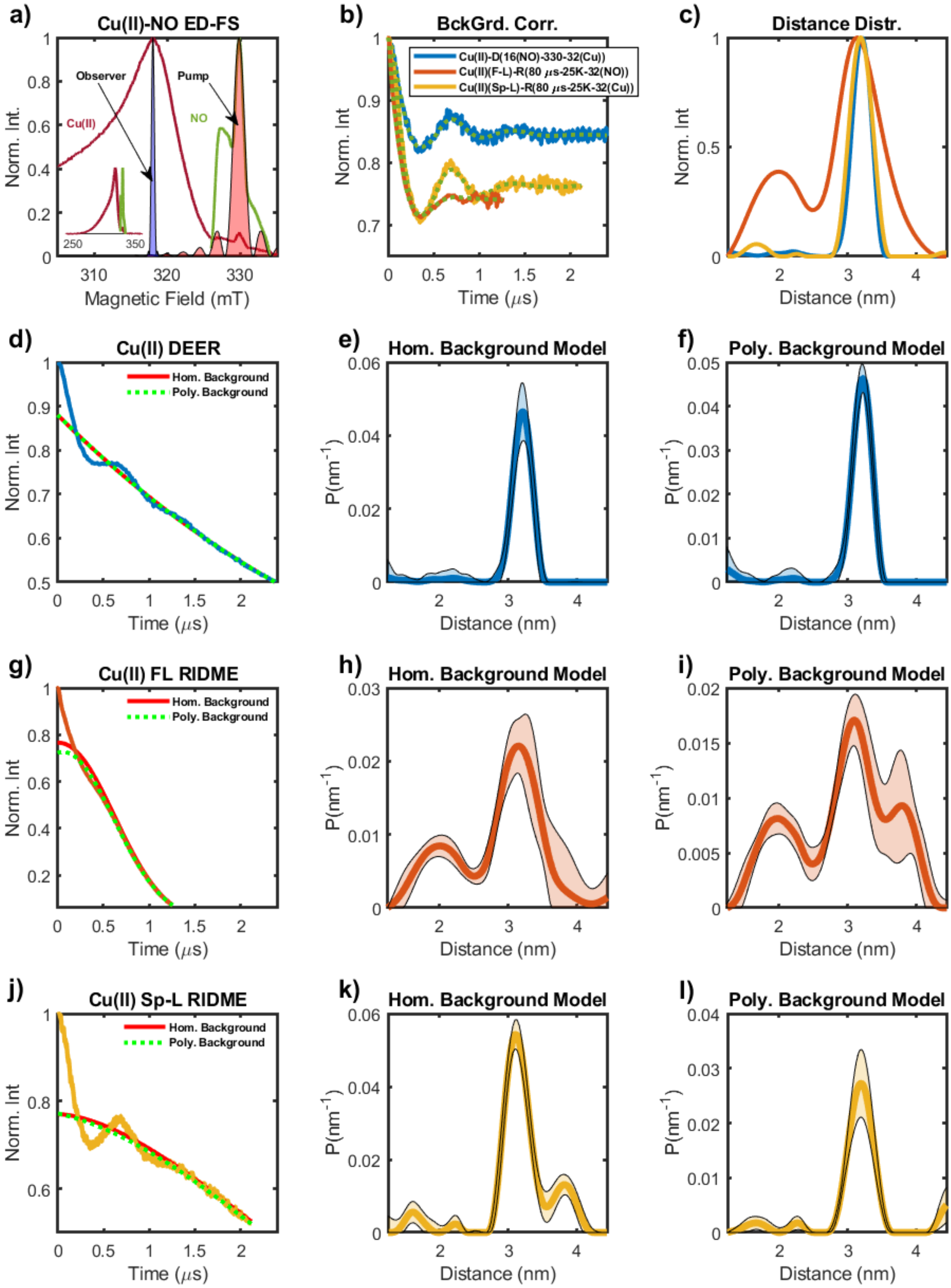

Figure S27. Cu(II)(MB1-2_{TOAC})$_3$ PDS data. (a) ED-FS profile of the TOAC (green) and Cu(II) (maroon), with the excitation profiles of the observer (blue) and pump (red) superimposed over the spectral pulse positions. The pulse profiles are shown on a limited spectral view, with the full ED-FS shown in the insert. (b) the PDS data after background correction for four-pulse DEER (D) of Cu(II)(MB1-2_{TOAC})$_3$ (blue), five-pulse RIDME (R) for fully labelled (F-L) Cu(II)(MB1-2_{TOAC})$_3$ (orange), and Cu(II)(MB1-2_{TOAC})$_3$ (Sp-L) RIDME (yellow). The distance distributions corresponding to the time traces in (b). (d, g, j) show the raw data for (b) with background corrections. (e, f, h, i, k, l) distance distributions after homogenous or polynomial background correction and their validations. (d) Cu(II)-nitroxide DEER with (e) Dim. 3, m = 3.21 nm, Avg. = 3.17 nm, σ = 0.25 nm, α = 79.4328 and (f) and 2nd-order polynomial, m = 3.21 nm, Avg. = 3.16, σ = 0.27 nm, α = 79.4328. (g) Cu(II)-nitroxide (F-L) RIDME, with (g) Dim. 6.4, m = 3.14 nm, Avg. = 2.85, σ = 0.64, α = 100 and (h) 2nd-order polynomial, m = 3.21 nm, Avg. = 2.88 nm, σ = 0.72 nm, α = 100. (j) Cu(II)-nitroxide (Sp-L) RIDME with (k) Dim. 5.04, m = 3.20 nm, Avg. = 3.21 nm, σ = 0.30 nm, α = 100 and (l) 2nd-order polynomial, m = 3.20 nm, Avg. = 3.20 nm, σ = 0.22 nm, α = 100.

31. Figure S28:

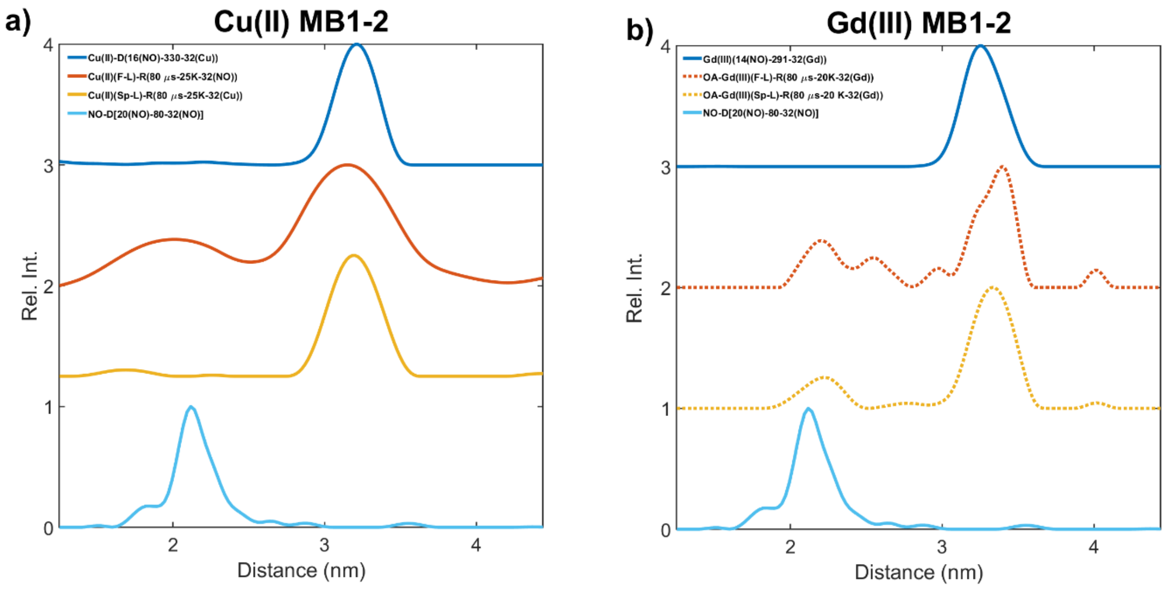

Figure S28: Distance distribution comparisons for the metal to nitroxide PDS data shown in Figures S25 and S26. Nitroxide-nitroxide DEER distance distributions (Figure S24, aqua) is presented in both panels to demonstrate that the F-L RIDME short distances may correspond to nitroxide-nitroxide dipolar coupling being measured by RIDME. (a) Cu(II)(MB1-2_{TOAC})$_3$ distance distributions for DEER (blue), RIDME F-L (red) and RIDME Sp-L (yellow). (b) Gd(III)(MB1-2_{TOAC})$_3$ distance distributions for DEER (blue), RIDME F-L OA, RIDME Sp-L OA (yellow).

32. Figure S29:

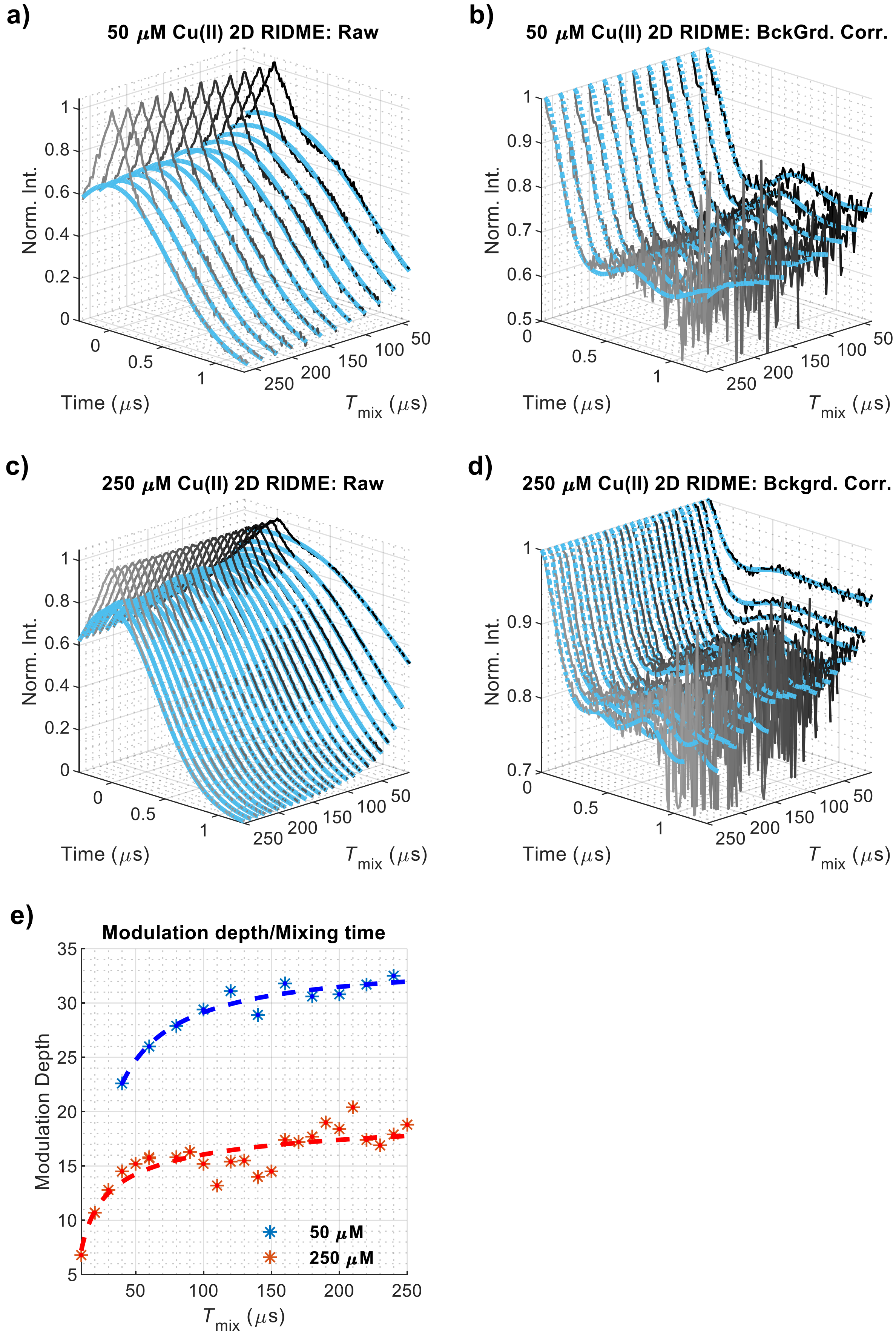

Figure S29: RIDME taken with various mixing times for 150 µM MB1-2 monomer (1:50 MB1-2_{TOAC}:MB1-2, sparsely labelled) with two concentrations of Cu(II). (a) 50 µM Cu(II) time traces. (b) as for (a) but after background correction. (c) 250 µM Cu(II) time traces. (d) as for (c) but after background correction. The DEERAnalysis calculated fits to the background (a,c) and experimental data (b,d) are shown in blue on the traces. (e) Modulation depth results fitted by a two-parameter power function (($f(x) = a * x^{b+c}$) , where the parameters are a = -37.82, b = -0.4284, c = 21.32 and a = -339.8b, = -0.9166, c = 34.13 for the 50 µM and 250 µM respectively). The modulation depths are greater for the 50 µM Cu(II) (which corresponds to a 1:3 ratio of Cu(II) to peptide monomer) than 250 µM Cu(II).

33. Figure S30:

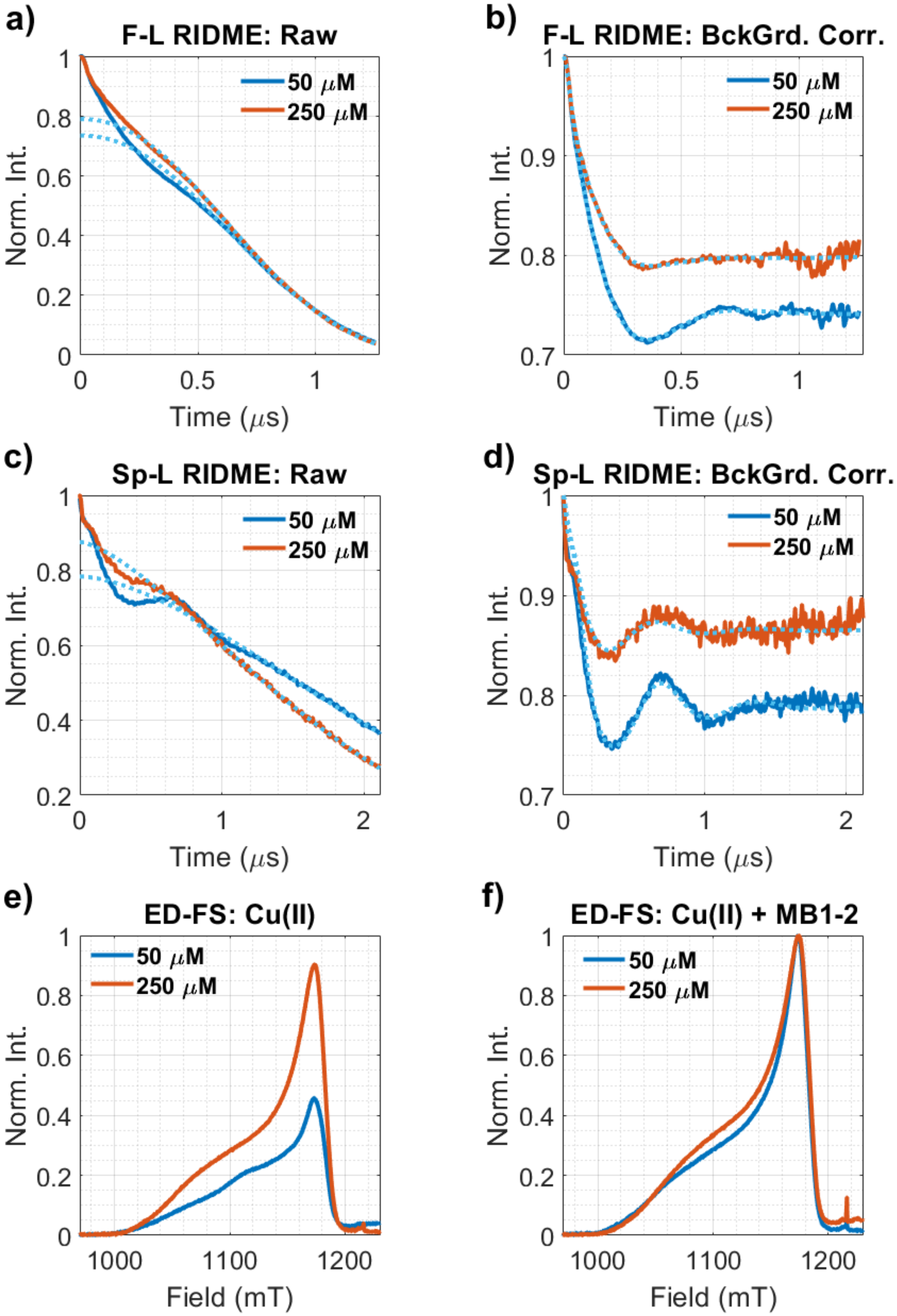

Figure S30: The effect on the RIDME and ED-FS of increasing the ratio of Cu(II) to MB1-2 monomer from 1:3 to 5:3. (a) Cu(II)(MB1-2_{TOAC})$_3$ F-L RIDME (150 µM peptide) with 50 and 250 µM Cu(II). (b) as for (a) but following background correction to clarify the effect of increasing Cu(II) concentration on reducing the modulation depth. (c) Cu(II)(MB1-2_{TOAC})$_3$ Sp-L RIDME (150 µM total peptide) with 50 µM and 250 µM Cu(II). (d) as for (c) but following background correction to clearly show the effect of increasing Cu(II) concentration on reducing the modulation depth. (e) ED-FS data for Cu(II) in buffer. (f) ED-FS for Cu(II) with MB1-2. In both (e) and (f) the signals are corrected for their relative acquisition gains and then normalized to the most intense signal (identical for both concentrations of Cu(II) with MB1-2), this provides a signal scaling for varying concentration.

34. Figure S31:

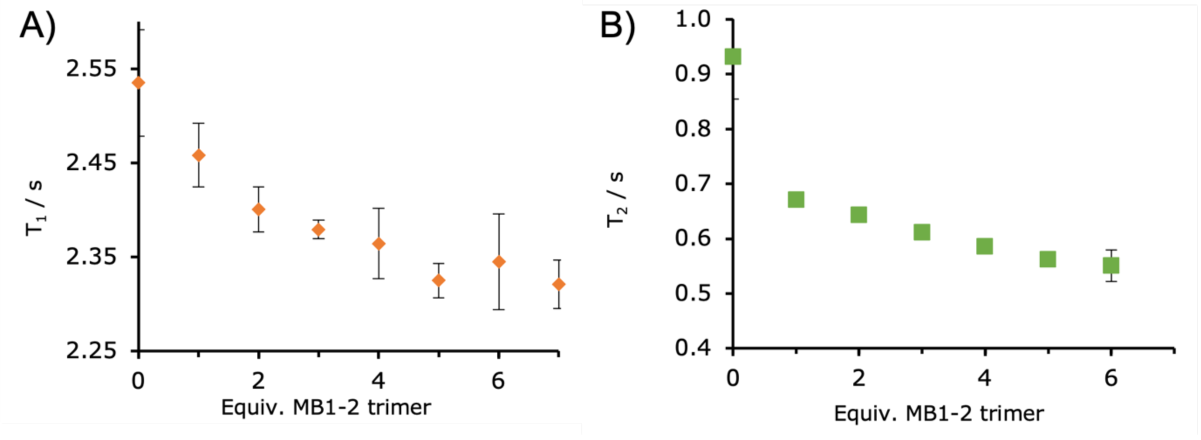

Figure S31: Plots of A) T_1 and B) T_2 ^{1}H magnetic resonance relaxation times for solutions of 40 µM $CuCl_2$ in 100 mM HEPES buffer pH 7.0, as a function of increasing equivalents MB1-2 peptide trimer. Samples recorded were at 293 K on a 300 MHz NMR spectrometer. Error bars determined from standard deviation of three independent repeat experiments.

35. Figure S32:

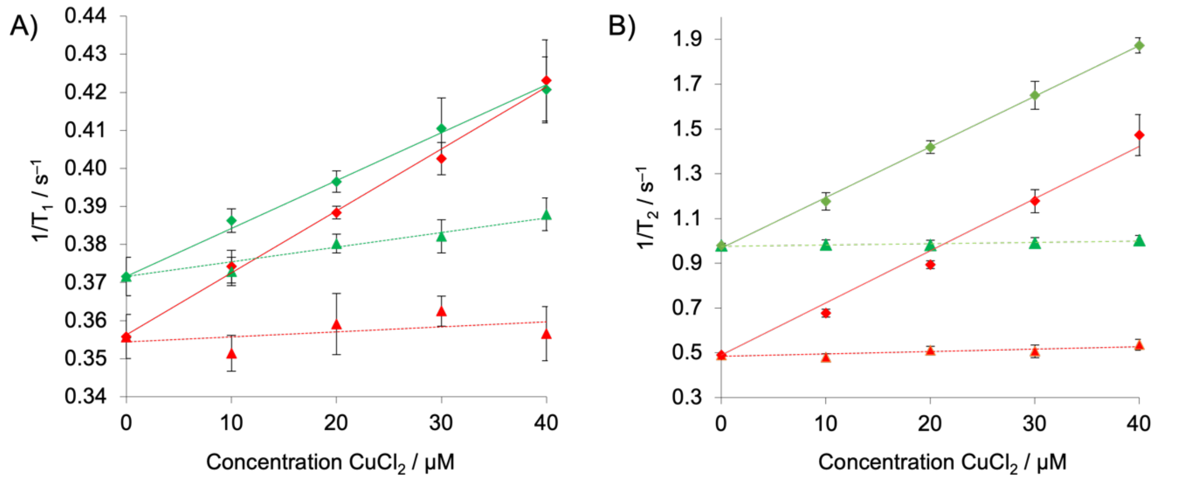

Figure S32: A) T_1 and B) T_2 ^{1}H magnetic resonance relaxivity plots, showing the reciprocal of ^{1}H magnetic resonance relaxation times as a function of [Cu^{2+}] concentration, recorded at pH 7, where samples were either manually adjusted (red) or prepared in 100 mM HEPES buffer (green), in the absence (triangles) and presence of 5 equivalents MB1-2 trimer (diamonds). Samples were recorded at 293 K on a 300 MHz NMR spectrometer. Error bars were determined from the standard deviation of three independent repeat experiments.

36. Figure S33:

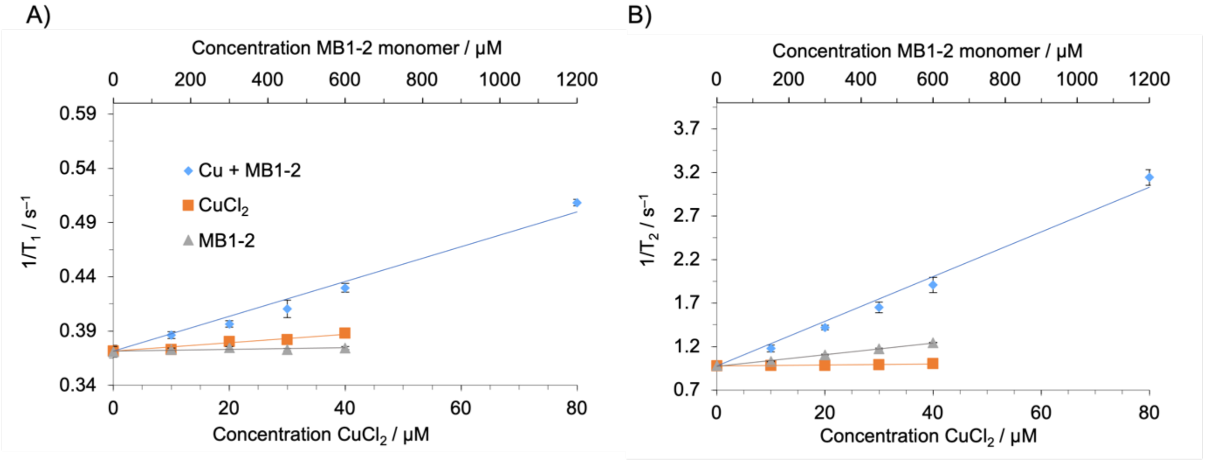

Figure S33: 300 MHz A) T_1 and B) T_2 ^{1}H magnetic resonance relaxivity plots, showing the reciprocal of ^{1}H magnetic resonance relaxation times, as a function of concentration for $CuCl_2$ (red square), apo MB1-2 (green triangle), and Cu(MB1-2)$_3$ (blue diamond). Cu(MB1-2)$_3$ samples prepared for Cu(II) in the presence of 5 equivalents MB1-2 peptide trimer. Samples recorded at 293 K, in the presence of 100 mM HEPES buffer pH 7.0 and on a 300 MHz NMR spectrometer. Error bars determined from standard deviation error of three independent repeat experiments.

37. Figure S34:

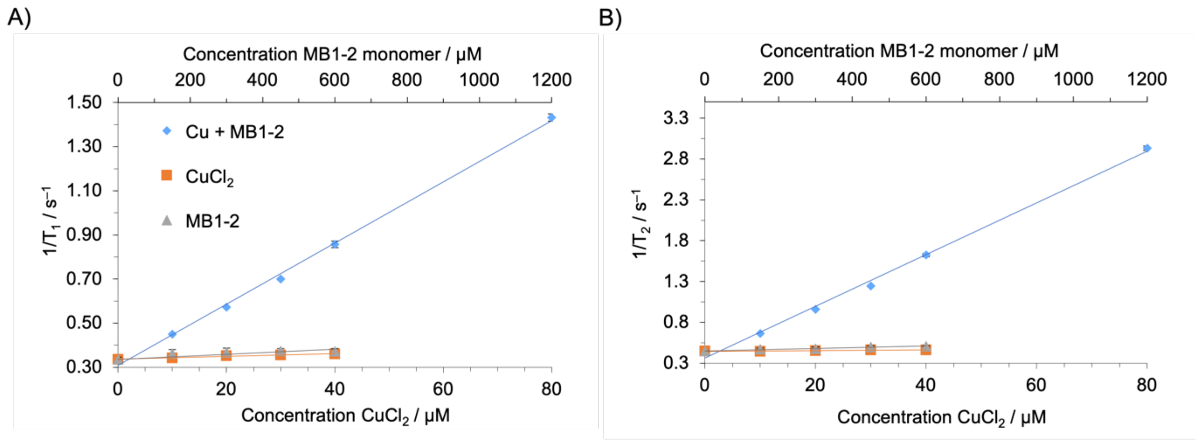

Figure S34: 60 MHz A) T_1 and B) T_2 ^{1}H magnetic resonance relaxivity plots, showing the reciprocal of ^{1}H magnetic resonance relaxation times as a function of concentration for apo MB1-2 (grey triangle), and Cu(MB1-2)$_3$ (blue diamond). Cu(MB1-2)$_3$ samples prepared for Cu(II) in the presence of 5 equivalents MB1-2 peptide trimer. Samples recorded at 293 K, in the presence of 100 mM HEPES buffer pH 7.0 and on a 60 MHz NMR spectrometer. Error bars determined from standard deviation error of three independent repeat experiments.

38. Figure S35:

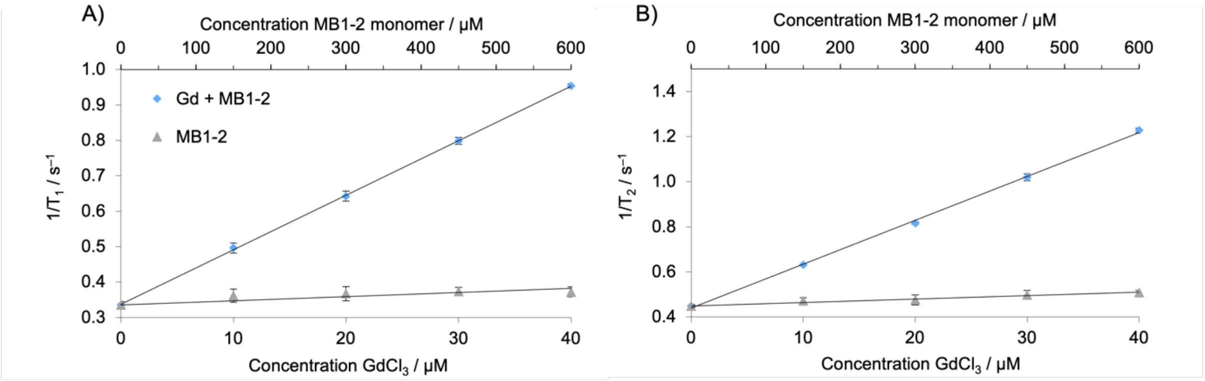

Figure S35: A) T_1 and B) T_2 ^{1}H magnetic resonance relaxivity plots, showing the reciprocal of ^{1}H magnetic resonance relaxation times, as a function of concentration for apo MB1-2 (grey triangle) and $Gd(MB1\text{-}2)_3$ (blue diamond). $Gd(MB1\text{-}2)_3$ samples prepared for Gd(III) in the presence of 5 equivalents MB1-2 peptide trimer. Samples recorded at 293 K, in the presence of 100 mM HEPES buffer pH 7.0 and on a 60 MHz NMR spectrometer. Error bars determined from standard deviation error of three independent repeat experiments.

39. Figure S36:

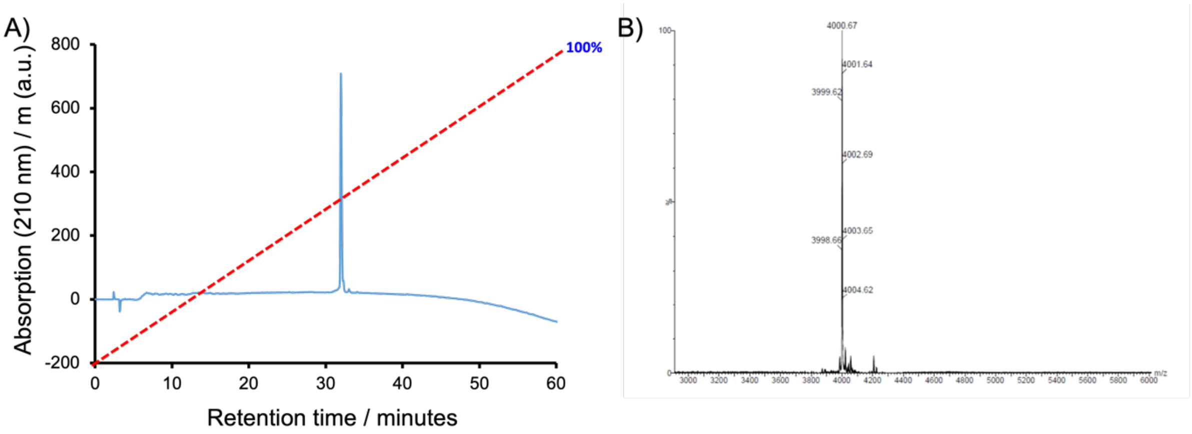

Figure S36. A) Analytical reverse phase C18-HPLC chromatograph of purified MB1C using H_2O/MeCN gradient (0 – 100% MeCN over 60 minutes) in the presence of 0.1% TFA. B) MALDI mass spectrum of purified MB1C.

40. Figure S37:

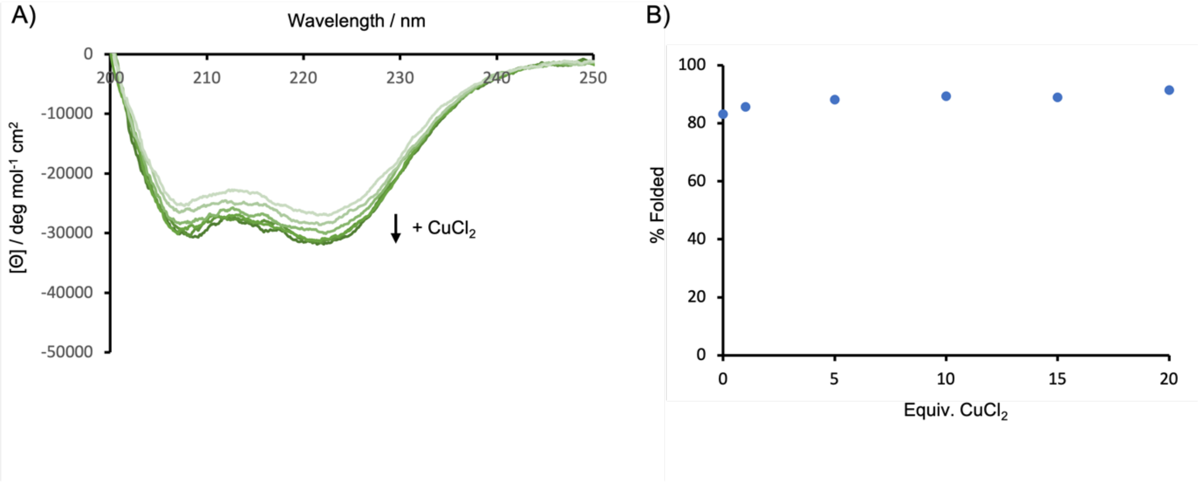

Figure S37. $CuCl_2$ (0-200 µM) titration into 31 µM MB1C peptide monomer in 10 mM HEPES buffer pH 7.0, monitored by CD (A). The plot of % folded, based on the molar ellipticity at 222 nm, as a function of $CuCl_2$ concentration, is shown in panel (B).

41. Figure S38:

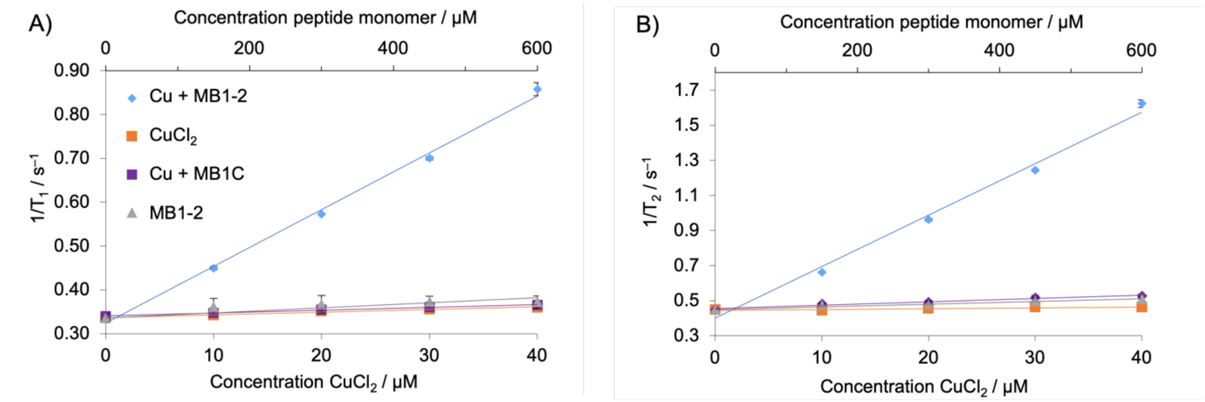

Figure S38. A) T_1 and B) T_2 ^{1}H magnetic resonance relaxivity plots, showing the reciprocal of ^{1}H magnetic resonance relaxation times as a function of concentration, for $CuCl_2$ in the presence of 5 equiv. MB1C (purple squares) or MB1-2 (blue diamonds) peptide trimer. Data is also shown for apo MB1-2 (grey triangle) and $CuCl_2$ (orange squares). Samples recorded at 293 K, in the presence of 100 mM HEPES buffer pH 7.0 and on a 60 MHz NMR spectrometer. Error bars determined from standard deviation error of three independent repeat experiments.

42. References:

1. H. E. Mash, Y. P. Chin, L. Sigg, R. Hari, H. Xue, Complexation of copper by zwitterionic aminosulfonic (good) buffers. *Anal Chem* **75**, 671-677 (2003).
2. C.-Q. Xiao, Q. Huang, Y. Zhang, H.-Q. Zhang, L. Lai, Binding thermodynamics of divalent metal ions to several biological buffers. *Thermochim. Acta* **691**, 178721 (2020).
3. M. R. Berwick *et al.*, Location dependent coordination chemistry and MRI relaxivity, in de novo designed lanthanide coiled coils. *Chem Sci* **7**, 2207-2216 (2016).
4. W. Chan, P. White, *Fmoc solid phase peptide synthesis: a practical approach* (OUP Oxford, 1999), vol. 222.
5. E. Kaiser, R. L. Colescott, C. D. Bossinger, P. I. Cook, Color test for detection of free terminal amino groups in the solid-phase synthesis of peptides. *Anal Biochem* **34**, 595-598 (1970).
6. O. Schlenker, K. Rittinger, Structures of dimeric GIT1 and trimeric beta-PIX and implications for GIT-PIX complex assembly. *J Mol Biol* **386**, 280-289 (2009).
7. K. N. Beasley *et al.*, "Chapter Twenty-One - Computer Modeling of Spin Labels: NASNOX, PRONOX, and ALLNOX" in Methods Enzymol., P. Z. Qin, K. Warncke, Eds. (Academic Press, 2015), vol. 563, pp. 569-593.
8. https://ihlab.hsc.usc.edu/allnox/ (
9. M. Mirdita *et al.*, ColabFold: making protein folding accessible to all. *Nature Methods* **19**, 679-682 (2022).
10. S. L. Dürr, A. Levy, U. Rothlisberger, Accurate prediction of transition metal ion location via deep learning. *bioRxiv*, 2022.2008.2022.504853 (2022).
11. A. Barge, G. Cravotto, E. Gianolio, F. Fedeli, How to determine free Gd and free ligand in solution of Gd chelates. A technical note. *Contrast Media Mol. Imaging* **1**, 184-188 (2006).
12. J. K. Myers, C. N. Pace, J. M. Scholtz, A direct comparison of helix propensity in proteins and peptides. *Proc Natl Acad Sci U S A* **94**, 2833-2837 (1997).
13. S. Diaz-Moreno *et al.*, The Spectroscopy Village at Diamond Light Source. *J Synchrotron Radiat* **25**, 998-1009 (2018).
14. S. Hayama *et al.*, The scanning four-bounce monochromator for beamline I20 at the Diamond Light Source. *J Synchrotron Radiat* **25**, 1556-1564 (2018).
15. G. Dennis *et al.*, First results using the new DLS Xspress4 digital pulse processor with monolithic segmented HPGe detectors on XAS beamlines. *AIP Conf. Proc.* **2054**, 060065 (2019).
16. B. Ravel, M. Newville, ATHENA, ARTEMIS, HEPHAESTUS: data analysis for X-ray absorption spectroscopy using IFEFFIT. *J Synchrotron Radiat* **12**, 537-541 (2005).
17. J. J. Rehr, R. C. Albers, Theoretical approaches to x-ray absorption fine structure. *Rev. Mod. Phys.* **72**, 621-654 (2000).
18. C. G. Hoogstraten, R. D. Britt, Water counting: quantitating the hydration level of paramagnetic metal ions bound to nucleotides and nucleic acids. *RNA* **8**, 252-260 (2002).
19. B. E. Bode *et al.*, Counting the monomers in nanometer-sized oligomers by pulsed electron-electron double resonance. *J Am Chem Soc* **129**, 6736-6745 (2007).

20. D. Hilger *et al.*, Assessing oligomerization of membrane proteins by four-pulse DEER: pH-dependent dimerization of NhaA Na+/H+ antiporter of E. coli. *Biophys J* **89**, 1328-1338 (2005).
21. A. M. Bowen *et al.*, Exploiting orientation-selective DEER: determining molecular structure in systems containing Cu(II) centres. *Phys Chem Chem Phys* **18**, 5981-5994 (2016).
22. G. Jeschke *et al.*, DeerAnalysis2006—a comprehensive software package for analyzing pulsed ELDOR data. *Appl. Magn. Reson.* **30**, 473-498 (2006).
23. K. Keller *et al.*, Computing distance distributions from dipolar evolution data with overtones: RIDME spectroscopy with Gd(iii)-based spin labels. *Phys Chem Chem Phys* **19**, 17856-17876 (2017).
24. D. Abdullin, O. Schiemann, Pulsed Dipolar EPR Spectroscopy and Metal Ions: Methodology and Biological Applications. *Chempluschem* **85**, 353-372 (2020).
25. K. Gekko, S. N. Timasheff, Mechanism of protein stabilization by glycerol: preferential hydration in glycerol-water mixtures. *Biochemistry* **20**, 4667-4676 (1981).
26. K. Gekko, S. N. Timasheff, Thermodynamic and kinetic examination of protein stabilization by glycerol. *Biochemistry* **20**, 4677-4686 (1981).
27. E. Durr, I. Jelesarov, Thermodynamic analysis of cavity creating mutations in an engineered leucine zipper and energetics of glycerol-induced coiled coil stabilization. *Biochemistry* **39**, 4472-4482 (2000).
28. L. N. Slope, O. J. Daubney, H. Campbell, S. A. White, A. F. A. Peacock, Location-Dependent Lanthanide Selectivity Engineered into Structurally Characterized Designed Coiled Coils. *Angewandte Chemie International Edition* **60**, 24473-24477 (2021).
29. E. Garribba, G. Micera, The Determination of the Geometry of Cu(II) Complexes: An EPR Spectroscopy Experiment. *J. Chem. Educ.* **83**, 1229 (2006).
30. J. Peisach, W. E Blumberg, Structural implications derived from the analysis of EPR spectra of natural and artificial copper proteins. *Arch. Biochem. Biophys.* **165**, 691-708 (1975).
31. P. Kuzmic, Program DYNAFIT for the analysis of enzyme kinetic data: application to HIV proteinase. *Anal Biochem* **237**, 260-273 (1996).
